# 5,10,15,20‐Tetrakis(pentafluorophenyl)porphyrin as a Functional Platform for Peptide Stapling and Multicyclisation

**DOI:** 10.1002/chem.202301410

**Published:** 2023-09-06

**Authors:** Paolo Dognini, Talhat Chaudhry, Giulia Scagnetti, Michele Assante, George S. M. Hanson, Kehinde Ross, Francesca Giuntini, Christopher R. Coxon

**Affiliations:** ^1^ School of Pharmacy and Biomolecular Sciences Liverpool John Moores University Byrom Street Campus L3 3AF Liverpool UK; ^2^ EaStCHEM School of Chemistry The University of Edinburgh Joseph Black Building, David Brewster Road EH9 3FJ Edinburgh UK

**Keywords:** aromatic substitution, fluorine, peptides, peptide multicyclisation, peptide stapling, porphyrins, porphyrins strapping

## Abstract

Polyfluorinated aromatic reagents readily react with thiolates via nucleophilic aromatic substitution (S_*N*_Ar) and provide excellent scaffolds for peptide cyclisation. Here we report a robust and versatile platform for peptide stapling and multicyclisation templated by 5,10,15,20‐tetrakis(pentafluorophenyl)porphyrin, opening the door to the next generation of functional scaffolds for 3D peptide architectures. We demonstrate that stapling and multicyclisation occurs with a range of non‐protected peptides under peptide‐compatible conditions, exhibiting chemoselectivity and wide‐applicability. Peptides containing two cysteine residues are readily stapled, and the remaining perfluoroaryl groups permit the introduction of a second peptide in a modular fashion to access bicyclic peptides. Similarly, peptides with more than two cysteine residues can afford multicyclic products containing up to three peptide ‘loops’. Finally, we demonstrate that a porphyrin‐templated stapled peptide containing the Skin Penetrating and Cell Entering (SPACE) peptide affords a skin cell penetrating conjugate with intrinsic fluorescence.

## Graphical abstract

We introduce 5,10,15,20‐tetrakis(pentafluorophenyl)porphyrin as a widely applicable scaffold for the stapling and multicyclisation of cysteine‐containing peptides. A robust and peptide‐compatible Nucleophilic Aromatic Substitution between aromatic fluorides and cysteine thiols was performed with a library of peptides, leading to different cyclic species – one of which was biologically tested – and offering relevant synthetic insights.

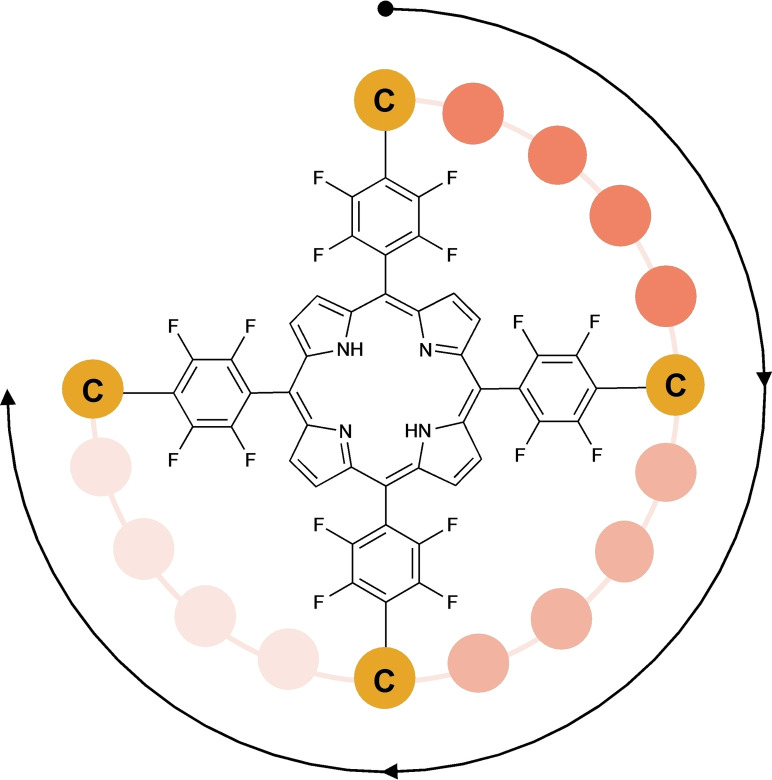

## Introduction

Peptide cyclisation strategies have received significant attention in recent years due to the widely recognised advantages of cyclic peptides for biomedical applications.[^1^, ^2^, ^3^, ^4^] Compared to linear analogues, cyclisation typically confers greater biological stability whilst also exhibiting better defined conformational and structural robustness, resulting in improved interactions with biological targets.[^5^, ^6^] Cyclic peptides are found frequently in natural products and represent around half of regulatory approved pharmaceutical peptides on the market.[^7^, ^8^]

**Figure 1:**
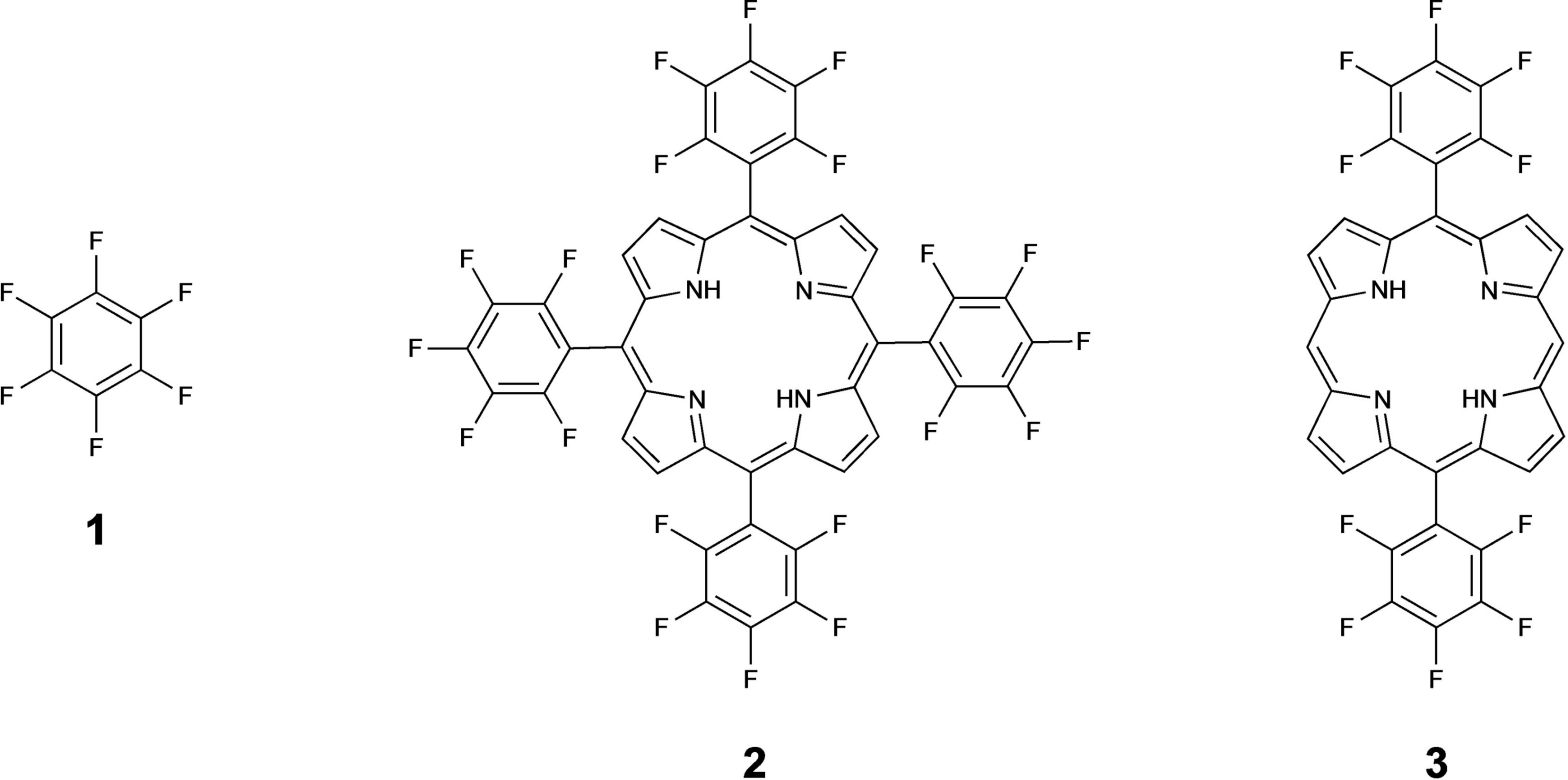
Chemical structure of hexafluorobenzene (**1**), 5,10,15,20‐tetrakis (pentafluorophenyl)porphyrin (**2**) and 5,15‐bis(pentafluorophenyl)porphyrin (**3**).

Important milestones in the chemistry of side‐chain to side‐chain peptide cyclisation were the introduction of peptide stapling by olefin metathesis by Miller and Grubbs in 1995^[9]^ and, 10 years later, the Chemical LInkage of Peptides onto Scaffolds (CLIPS) technology developed by Timmerman, which affords multicyclic peptide architectures.^[10]^ During the last decade, a growing number of alternative macrocyclisation approaches have been developed. Among these, cysteine crosslinking using polyfluoroaromatics such as hexafluorobenzene (**1**, Figure 1), originally reported by Pentelute, is particularly relevant to this work.^[11]^ Current scaffolds employed for peptide cyclisation are mostly small molecules that perform a purely structural role. However, recent trends show that the integration of a functional platform within a two‐component cyclic architecture can impart an additional desired character to the conjugate (e.g., luminescence, photo‐responsiveness or catalytical activity). This is the next step forward for the development of next generation peptide‐based multifunctional molecules.[^12^, ^13^, ^14^, ^15^, ^16^, ^17^, ^18^]

We have previously optimised the thiol‐fluoride substitution model reaction between N‐acetylcysteine and **1**, using ^19^F NMR and Design of Experiments (DOE), and translated this into a rapid (<1 min) peptide stapling method.^[19]^ Subsequently, polyfluorinated porphyrins were chosen to explore the use of novel and more complex scaffolds for conjugating peptides and templating their multicyclisation.

Porphyrins are widely available in nature and have attracted much interest from the scientific community for their versatile properties. The distinctive tetrapyrrole ring confers chemical stability, ability to coordinate a variety of metal ions with potential for catalytic activities, oxygen‐sensitivity, and light‐dependent properties such as fluorescence, generation of singlet oxygen, reactive oxygen species (ROS) or heat.[^20^, ^21^, ^22^] Peptide‐porphyrin (multivalent)conjugates – usually obtained via a single point of attachment between each peptide and the porphyrin[^23^, ^24^, ^25^] – are reported to be effective oxygen sensors, fluorescent probes, agents for photodynamic therapy (PDT) and photothermal therapy (PTT), drug delivery tools as well as self‐assembling light‐responsive materials.[^26^, ^27^]

In complex systems such as heme‐containing enzymes, the three‐dimensional architecture and microenvironment surrounding the porphyrin ring are pivotal for the stability and reactivity of the construct and can influence its photophysical and photochemical properties. As a result, starting with the pioneering works of Lehn^[28]^ and Sauvage^[29]^, a lot of effort has been dedicated to the synthesis of ‘strapped’ porphyrins, with the aim of mimicking tetrapyrrolic cofactors within a protein architecture. These systems are usually constructed using carbon‐based bridges (aliphatic or aromatic) that connect *ortho*/*meta* positions of asymmetrically substituted *meso*‐phenyl groups,[^30^, ^31^] but the possibility to employ straps of different chemical natures will be transformative within this field. Specifically, the use of peptides for porphyrin strapping would allow access to a new chemical space.

Peptide‐porphyrin cyclic architectures are interesting macromolecules that could have potential applications as natural enzyme mimics, photo‐functional systems, catalysts, sensors, probes, diagnostic agents, molecular cages as reported for conjugates.[^26^, ^27^] Besides having biological activity, it has been shown that cyclic peptides can also mimic epitopes, reactive sites, or cavities of natural proteins.^[32]^ Therefore, combining bioactive peptides and photoactive porphyrins can provide new chemical tools with diverse properties.

Karpishin and co‐workers cyclised a 22‐residue α‐helical peptide over the face of a metalloporphyrin via *meta* iminodiacetate groups on porphyrin 5 and 15 *meso*‐phenyls, which formed tridentate complexes with copper(II) ions, and allowed further coordination with histidine residues within the peptide.^[33]^ Concurrently, Geier and Sasaki obtained a covalent cyclic product from the reaction of an α‐helical peptide, presenting two cysteines with *i, i+11* spacing, with tetraphenylporphyrin via a *meta* bromoacetamide linker.[^34^, ^35^] Despite this promising field, the cyclisation reaction between porphyrins and unprotected peptides of different lengths, amino acid sequences and properties has been explored very little.

The synthetically accessible and commercially available 5,10,15,20‐tetrakis(pentafluorophenyl)porphyrin (**2**, Figure 1) contains four equivalent reactive sites for thiol‐fluoride substitution and can act as a platform for peptide cyclisation through reaction with two or more cysteine residues. Here, we explore the reactions between a range of cysteine containing peptides with polyfluorinated porphyrins, demonstrating a novel approach for accessing stapled and multicyclic peptide systems that integrate a tetrapyrrole ring.

## Results and Discussion

Preliminary experiments were performed to explore the conjugation of polyfluorinated porphyrin **2** with N‐acetylcysteine or a model peptide containing one cysteine (CAAAK). This confirmed that regioselective substitution at only the *para‐*position of *meso‐*pentafluorophenyl groups is more efficient (faster and higher conversion) using a base/solvent combination of Cs_2_CO_3_ and DMSO rather than DIPEA and DMF (see Supporting Information), and this is generally in agreement with our prior results using **1**.^[19]^ Therefore, Cs_2_CO_3_/DMSO were selected as a starting condition to develop a conjugation strategy for a fully‐deprotected cysteine containing peptide in solution.

As part of an effort to design skin‐penetrating porphyrins for photodynamic applications, the Skin Penetrating and Cell Entering (SPACE) peptide (**4** in Table 1)[^36^, ^37^] was employed for conjugation with porphyrin **2**. This peptide contains two cysteines located with relative *i, i+8* spacing, and the initial model reaction between peptide **4** and porphyrin **2** afforded a monocyclic peptide (a single peptide macrocycle around the porphyrin). Although S_N_Ar and thiol‐fluoride substitutions have already been exploited for the decoration of fluorinated porphyrins with different small molecules,[^23^, ^38^, ^39^, ^40^, ^41^, ^42^] this is one of the few examples involving cysteine thiolates for peptide‐porphyrin conjugation.^[24]^

**Table 1:** Reactivity of di‐cystine containing peptides with **2**. Conversion % represents a crude yield calculated from HPLC chromatograms (see Supporting Information).

Peptide	Sequence	Cys−Cys Spacing	S−S Distance [Å]	Monocyclic^[a]^	Bicyclic	Dimer^[b]^	Other
**4**	A **C** TGS TQHQ **C** G	*i*, *i+8*	22.59	46 % (**19**)	4 %	10 %	40 %
**5**	A **C** ATG STQHQ A **C** G	*i*, *i+10*	28.08	35 % (**20**)	1 %	12 %	52 %
**6**	A **C** ATG STQHQ **C** G	*i*, *i+9*	26.27	36 % (**21**)	4 %	10 %	50 %
**7**	A **C** TST QHQ **C** G	*i*, *i+7*	21.29	50 % (**22**)	4 %	6 %	40 %
**8**	A **C** STQ HQ **C** G	*i*, *i+6*	19.94	56 % (**23**)	8 %	3 %	33 %
**9**	A **C** TQH Q **C** G	*i*, *i+5*	14.81	65 % (**24**)	14 %	4 %	17 %
**10**	A **C** QHQ **C** G	*i*, *i+4*	14.67	65 % (**25**)	6 %	4 %	25 %
**11**	A **C** HQ **C** G	*i*, *i+3*	10.31	65 % (**26**)	22 %	2 %	11 %
**12**	A **C** A **C** G	*i*, *i+2*	8.23	6 % (**27**)	46 %	1 %	47 %
**13**	A **C** H **C** G	*i*, *i+2*	7.97	43 % (**28**)	17 %	5 %	35 %
**14**	A **CC** G	*i*, *i+1*	6.83	–	–	–	100 %
**15**	A **C** THG QTQS **C** G	*i*, *i+8*	24.49	47 % (**29**)	3 %	7 %	43 %
**16**	N **C** VVG YIGER **C** Q	*i*, *i+9*	25.27	36 % (**30**)	3 %	14 %	47 %
**17**	**C** KAPE TAL **C**	*i*, *i+8*	22.16	44 % (**31**)	4 %	2 %	50 %
**18**	AGYLL GKINL KA **C** AA LAKK **C** L^[c]^	*i*, *i+7*	N.A.	72 % (**32**)	2 %	6 %	20 %

[a] The number in parentheses is the stapled product compound number; [b] Porphyrin dimers bear two chromophores, and therefore, the relative abundance could be overestimated compared to cyclic peptides with only one porphyrin ring; [c] Purified peptide.

**Figure 2:**
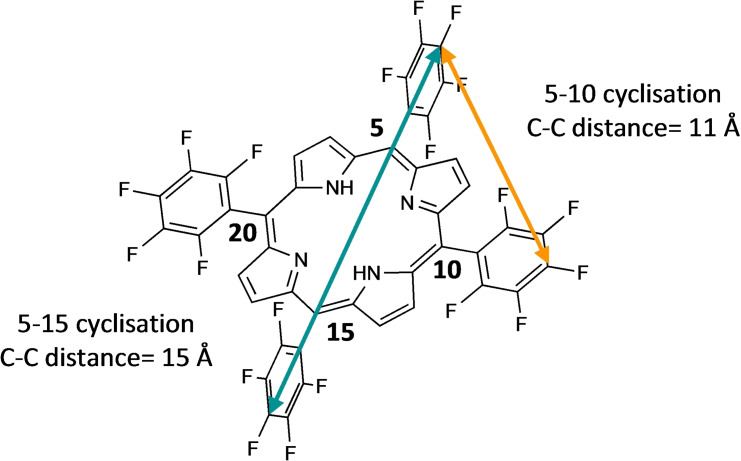
5–15 cyclisation (teal) and 5–10 cyclisation (tangerine) geometry for the reaction of di‐cysteine containing peptides on **2**.

The cyclisation reaction between a peptide displaying two cysteine residues and porphyrin **2** potentially leads to two products: one resulting from the substitution of the *para*‐fluorides on the *meso*‐aryl rings in positions 5 and 15 (hereafter referred to as 5–15 cyclisation product) and the other resulting from the related reaction on the rings in 5–10 *meso* positions (hereafter referred to as 5–10 cyclisation product, Figure 2).

Computational modelling of porphyrin **2** using the semiempirical XTB method[^43^, ^44^] (details in Experimental section) estimated a distance of around 15 Å and 11 Å between the 4‐position carbon atoms of pentafluorophenyl groups in the 5–15 and 5–10 positions, respectively. Using the same approach, we estimated the inter‐thiol (S−S) distance within the SPACE peptide to be approximately 23 Å, which was predicted to allow both 5–10 and 5–15 cyclisation to occur. Therefore, we undertook this study to identify the minimal inter‐cysteine distance that would direct the cyclisation to occur preferentially at the 5–10 positions. A controlled 5–10 cyclisation would be preferred because this would allow discrete control of the regioselectivity during peptide stapling and multicyclisation, and this geometry may be achieved by selection of the appropriate distance between the thiols.

Starting from SPACE peptide **4**, we designed a library of peptides containing pairs of cysteines and explored different distances between thiols to investigate the effect of the inter‐cysteine distance on the cyclisation (Table 1). To understand the optimal inter‐thiol length for 5–10 regioselectivity, we decreased the inter‐thiol distance down to *i, i+1* (peptides **7**–**14**) and we also increased this length up to *i, i+10* (peptides **5** and **6**) to verify eventual reactivity changes with longer peptides. The SPACE peptide control (**15**) – corresponding to the scrambled amino acid sequence of peptide **4** – was included for subsequent cellular uptake studies. Further example peptides comprise the cyclic region of the 53‐mer human epidermal growth factor (hEGF 32–43, **16**),^[45]^ the apamin‐derived peptide MiniAp‐3 (**17**)^[46]^ and the M13 peptide (**18**), an analogue of the peptide transportan‐10.[^47^, ^48^] The inclusion of these diverse peptide sequences allowed us to evaluate whether this reaction is sequence specific, to prove the selectivity of the thiolate‐fluoride substitution compared to nucleophiles commonly present in natural amino acids, and to assess its compatibility with residues such as proline. Moreover, the cyclisation of brain‐targeting peptides such as MiniAp‐3 and M13 with a polyfluorinated porphyrin may be particularly interesting, given the evidence that perfluoroarene‐based peptide macrocycles can have enhanced blood‐brain barrier penetration.[^47^, ^48^]

With further computational calculations on the whole peptide library, we estimated that cysteine spacings between *i, i+4* and *i, i+5* in unstructured peptides would be long enough to bridge the shorter distance of 11 Å for 5–10 cyclisation. The calculated sulfur‐sulfur distances are also listed in Table 1.

**Scheme 1:**
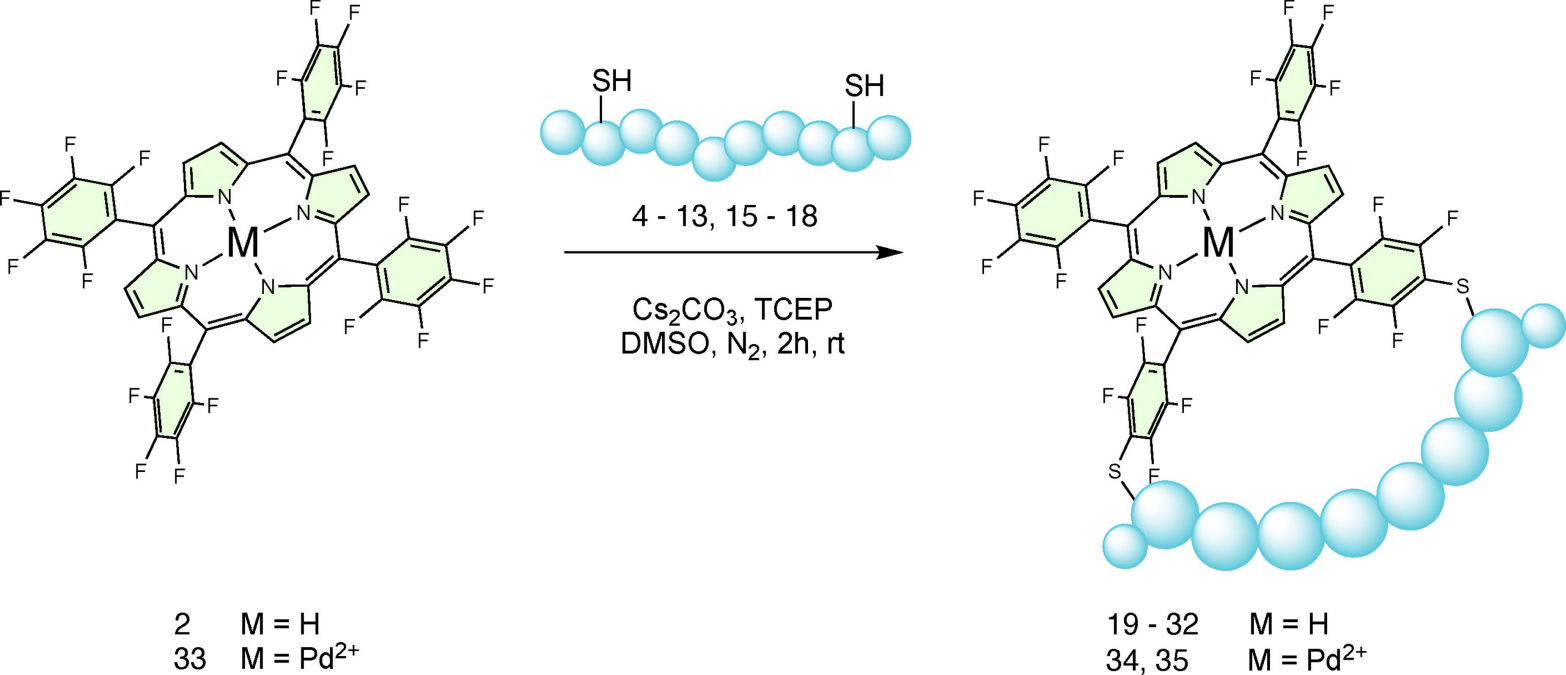
Reaction scheme for the cyclisation of di‐cysteine containing peptides on to **2**.

Stapled products (**19**–**32**) were formed in the reaction between **2** and each of the peptides, aside from the shortest inter‐thiol distance of *i, i+1* (**14**), indicating that the method is versatile, the reactions are not sequence‐specific, and the conditions are compatible with unprotected side chains (Scheme 1). For all peptides, one main peak – expected to be the 5–10 macrocyclisation product, was observed by HPLC/LC–MS. However, for peptides with longer inter thiol distances such as peptide **4** (*i, i+8)*, **7** (*i, i+7*) and **6** (*i, i+9*), a second peak with low peak area but with the same *m/z* ratio was also encountered. This can be ascribed to the presumably less favoured 5–15 regioisomer, confirming that the careful choice of the inter‐cysteine distance is pivotal to direct 5–10 stapling.

Reactions were carried out at room temperature, in the minimum volume of DMSO that dissolved all the reagents, under a nitrogen atmosphere and in the presence of the base Cs_2_CO_3_ and the non‐thiol reducing agent tris(2‐carboxyethyl) phosphine (TCEP) to limit the formation of oxidation products, including inter‐ and intramolecular peptide disulfides.

Time‐course experiments using equimolar ratios of porphyrin **2** and peptide **4**, showed that the stapled product **19** was readily obtained and no starting peptide **4** was observed by HPLC (215 nm) and LC–MS analysis after 3 h (Figure S9–10). In a control experiment, performed under the same reaction conditions but lacking porphyrin **2**, no significant degradation of peptide **4** (except for some disulfide form) was observed by HPLC/LC–MS after 3 h (Figure S8). This indicated complete consumption of starting peptide **4** and its conversion to porphyrin‐derived species.

The proportion of stapled products formed over the population of other peptide‐porphyrin species within the reaction crude mixtures is reported as % conversion in Table 1, and represents the crude yield measured by HPLC. Bicyclic peptides (two cyclic peptides on the same porphyrin) and porphyrin dimers (two porphyrin conjugated to the same peptide) were also tentatively identified along with unidentified side products. The chromatograms of reaction crude mixtures – in which the abovementioned products were labelled – are provided in the Supporting Information.

We next demonstrated that metal‐containing porphyrins, such as palladium 5,10,15,20‐tetrakis(pentafluorophenyl) porphyrin (**33**), successfully underwent stapling affording products **34** and **35** without any noticeable changes in the reactivity or outcomes compared with the metal‐free porphyrin (Scheme 1).

Notably, all the reactions proceeded well using crude peptides (except for peptide **18**), which again underlined the versatility and robustness of this cyclisation methodology as a rapid way to make peptide macrocycle libraries. Overall, these results indicate that the polyfluorinated porphyrin is a widely applicable and exploitable scaffold for peptide macrocyclisation.

Unreacted pentafluorophenyl groups exhibit three ^19^F NMR resonances at −136, −151 and −161 ppm with an integration pattern of 2 : 1 : 2. After the cyclisation, ^19^F NMR analysis (Figure S13–18) confirmed the substitution at two pentafluorophenyl groups affording two signals between −135 and −140 ppm, corresponding to *ortho* and *meta* fluorine atom pairs of the *para*‐disubstituted tetrafluorobenzene. However, these exhibit a complex multiplicity that can be ascribed to a loss of symmetry and hindered rotation of the fluorinated rings after peptide stapling. Although we calculated that longer inter‐thiol distances can afford the 5–15 cyclisation, the ^19^F NMR spectra obtained for both the peptides with shorter (e.g., *i, i+5* of peptide **9**) and longer (e.g., *i, i+8* of peptide **4**) inter‐thiol spacing appeared highly similar and there was no clear presence of different regioisomeric products.

**Scheme 2:**
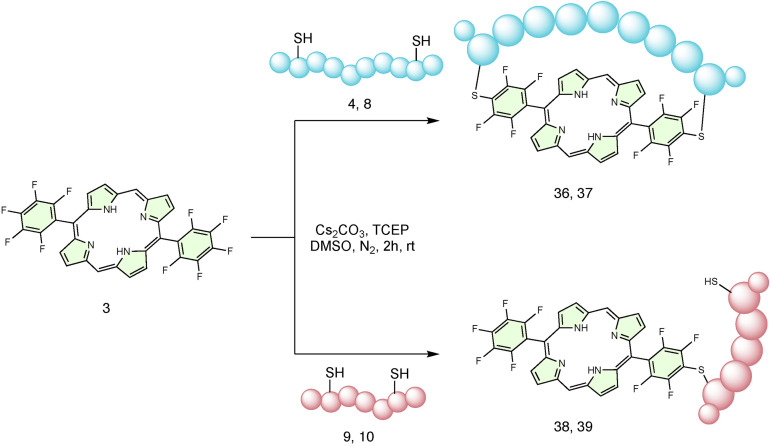
Reaction scheme for the cyclisation of di‐cysteine containing peptides on to **3**.

To gain further insight into how the number of inter‐thiol residues could be related to 5–10 cyclisation products, the cyclisation of key peptides with 5,15‐bis(pentafluorophenyl) porphyrin (**3**) was performed (Scheme 2). Compared with porphyrin **2**, this porphyrin lacks the two *meso*‐pentafluorophenyl groups in position 10 and 20 and can only afford the cyclic product with 5–15 geometry. As indicated in Table 2, cyclic products (**36** and **37**) were observed for peptides **4** (SPACE peptide) and **8**.

**Table 2:** Reactivity of di‐cysteine containing peptides with **3**. Conversion % represents a crude yield calculated from HPLC chromatograms (see Supporting Information).

Peptide	Sequence	Cys−Cys Spacing	S−S Distance [Å]	Monocyclic (compound no.)	Conjugate (compound no.)	Other
**4**	A **C** TGS TQHQ **C** G	*i*, *i+8*	22.59	55 % (**36**)	10 %	35 %
**8**	A **C** STQ HQ **C** G	*i*, *i+6*	19.94	10 % (**37**)	40 %	50 %
**9**	A **C** TQH Q **C** G	*i*, *i+5*	14.81	–	50 % (**38**)	50 %
**10**	A **C** QHQ **C** G	*i*, *i+4*	14.67	–	60 % (**39**)	40 %

The formation of product **36** (from peptide **4** (*i, i+8*) and porphyrin **3**) is comparable with previous examples of macrocyclisation, but the rate of formation was noticably slower, requiring 5 h for the reaction to reach completion. The reactivity of peptide **8** (*i, i+6*) towards porphyrin **3** appeared poorer, and product **37** was only obtained in a small amount even after a prolonged reaction time. This could be ascribed to the peptide hardly reaching the 5–15 position. Meanwhile, peptides **9** (*i, i+5*) and **10** (*i, i+4*), which were previously successfully cyclised around porphyrin **2**, did not afford cyclic species and only produced mono‐conjugation products (**38** and **39**).

Overall, these results suggested that *i, i+2* is the minimal inter‐thiol distance that allows 5–10 cyclisation while *i, i+6* is the minimal inter‐thiol distance that could also allow 5–15 cyclisation. Whilst the 5–10 cyclic product might be sterically and statistically preferred for inter‐thiol distances greater than *i, i+5*, further investigation is necessary to unequivocally identify the regioselectivity between 5–10 and 5–15 geometries and will be part of future experiments given that the purpose of this work was to achieve 5–10 cyclisation.

**Scheme 3:**
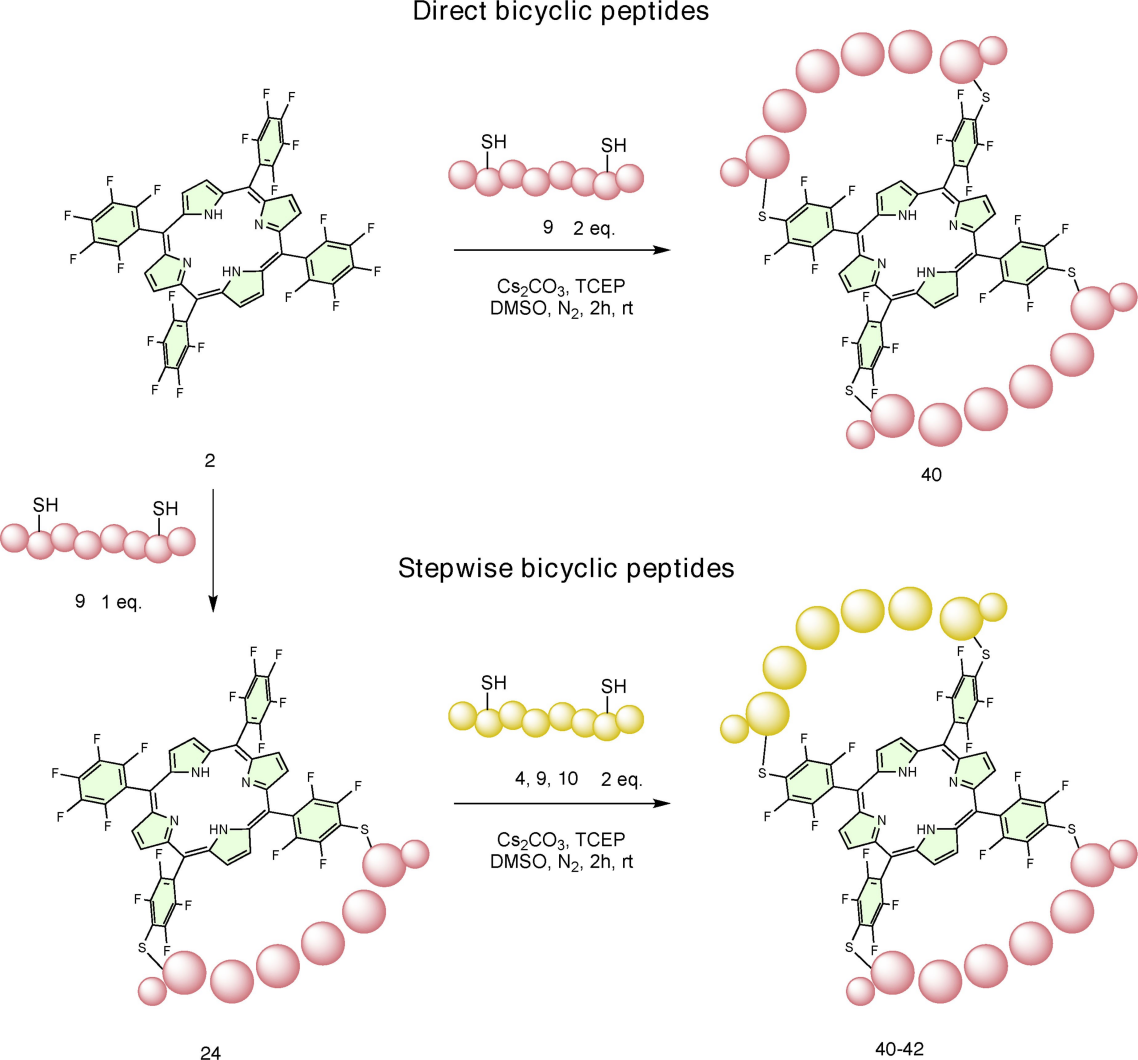
Reaction scheme for the synthesis of bicyclic peptides.

Following the successful installation and cyclisation of a single di‐cysteine containing peptide on porphyrin **2**, the possibility of performing multiple cyclisations was also explored (Scheme 3). When porphyrin **2** was treated with a two‐fold excess of peptide **9** (*i, i+5*), the direct conversion to a doubly stapled peptide (**40**), in which two molecules of the same peptide cyclised onto the porphyrin, was observed in higher percentage compared to experiments with the opposite ratio (Figure S11–12). This clearly indicated that the remaining *para*‐fluorine atoms are not deactivated by the initial substitutions.

Encouraged by the evidence that the four pentafluorophenyl rings can all engage in thiol‐fluoride substitution reactions, we also treated the crude monocyclic peptide **24** with more of the *i, i+5* peptide (**9**), and complete conversion to the corresponding heterobicyclic peptide product **40** was again obtained (Scheme 3). As a demonstration of the stepwise modularity of these systems, monocyclic peptide **24** was also treated with an excess of the *i, i+8* peptide (**4)** and *i, i+4* peptide (**10**) to obtain hetero‐bicyclic peptides **41** and **42**, respectively (Scheme 3). Thus, the reaction on monocyclic peptides can be performed with any other thiol (small molecule or peptide) to further functionalise the porphyrin scaffold. Likewise, with this modular approach, two different peptide sequences can be mixed and matched in a two‐step multicyclisation strategy to obtain multifunctional species and maintaining regiocontrol over the cyclisation, which has never been reported in the literature.

**Scheme 4:**
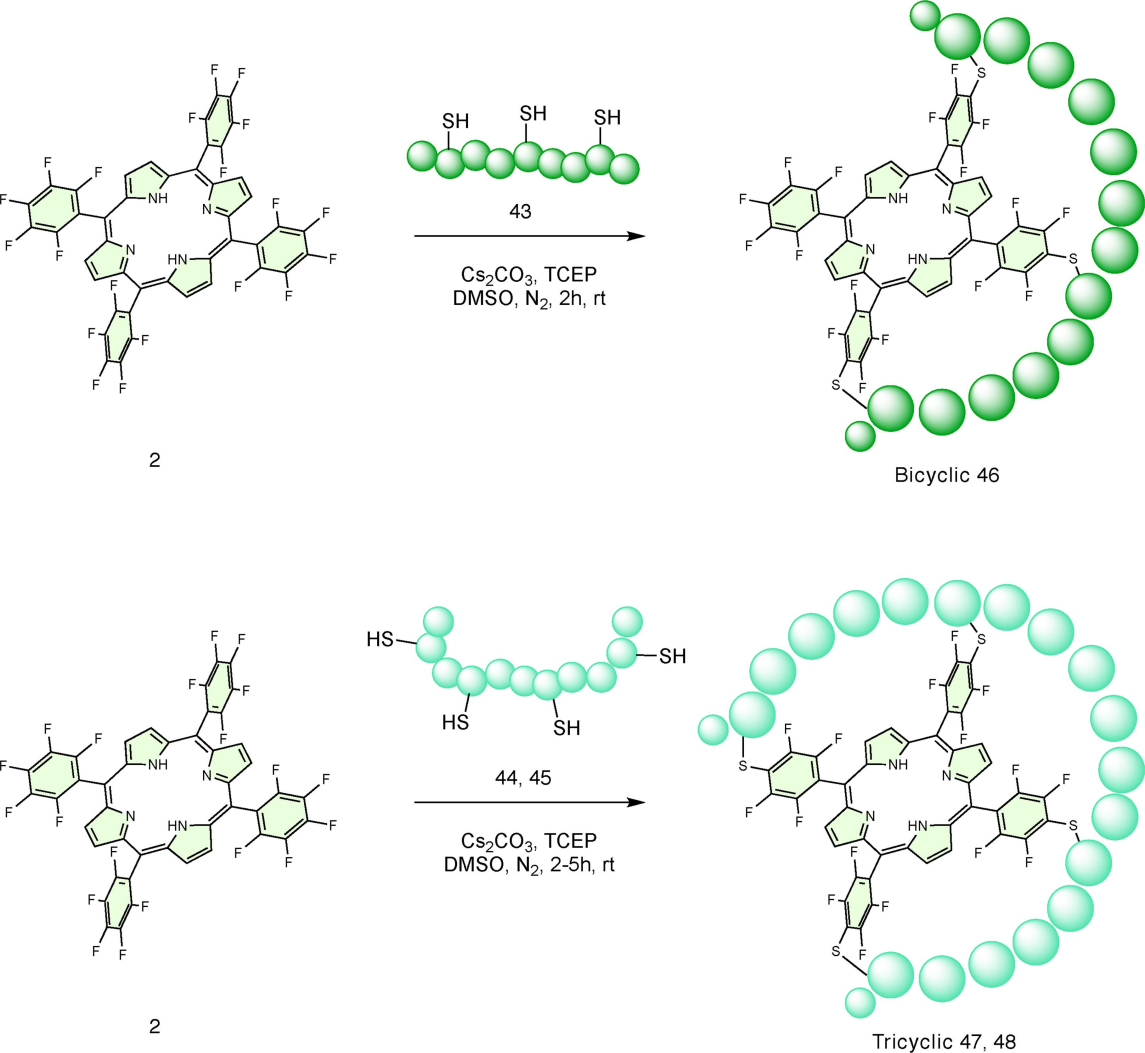
Reaction *scheme* for the one‐step synthesis of bicyclic and tricyclic peptides.

To explore the applicability of this cyclisation approach to peptides containing more than two cysteine residues, longer peptides **43**–**45** were synthesised (Table 3). As an inter‐thiol distance of *i*, *i+5* previously provided fast and clean conversion to 5–10 cyclic products, the CTQHQC motif was selected as a prototype repeating unit for multicyclisation. The bicyclic peptide (**46**, Scheme 4) was successfully obtained in one‐step from porphyrin **2** and peptide **43**, following the procedure previously described. Similarly, the tricyclic product (**47**, Scheme 4) was also synthesised using peptide **44**. Despite an extended reaction time (5 h), tricyclic product **47** formed as part of a mixture of regioisomers, side products and some incomplete cyclisation products. Peptide **45**, obtained using the *i, i+6* motif instead, afforded a higher percentage of the corresponding tricyclic product **48**, clearly because a greater distance between cysteine residues afforded greater flexibility, and therefore, complete multicyclisation. These results allow one step access to multicyclic peptide systems from peptides with multiple cysteine residues and are key findings for the future development of porphyrin‐templated multicyclic peptides. This is also further confirmation of the flexibility and level of control acquired over this chemical process.

**Table 3:** Reactivity of tri‐ and tetra‐cysteine containing peptides with **2**. Conversion % represents a crude yield calculated from HPLC chromatograms (see Supporting Information).

Peptide	Sequence	Cys−Cys Spacing	Multicyclic (compound no.)	Other
**43**	A **C** TQH Q **C** TQH Q **C** G	*i*, *i+5*	30 % (**46**)	70 %
**44**	A **C** TQH Q **C** TQH Q **C** TQH Q **C** G	*i*, *i+5*	20 % (**47**)	80 %
**45**	A **C** STQ HQ **C** ST QHQ **C** S TQHQ **C** G	*i*, *i+6*	34 % (**48**)	66 %

Therefore, it is clear that when the inter‐cysteine separation is between one (*i*, *i+2*) and four (*i*, *i+5*) amino acids, the 5–10 cyclic product is obtained. Meanwhile, if this distance is extended to five (*i*, *i+6*) or more residues, the peptide can also react in 5–15 position. After the cyclisation of a single peptide, the polyfluorinated scaffold can be further functionalised, and can be exploited to access bicyclic and tricyclic peptides via a one or two‐step process.

**Figure 3:**
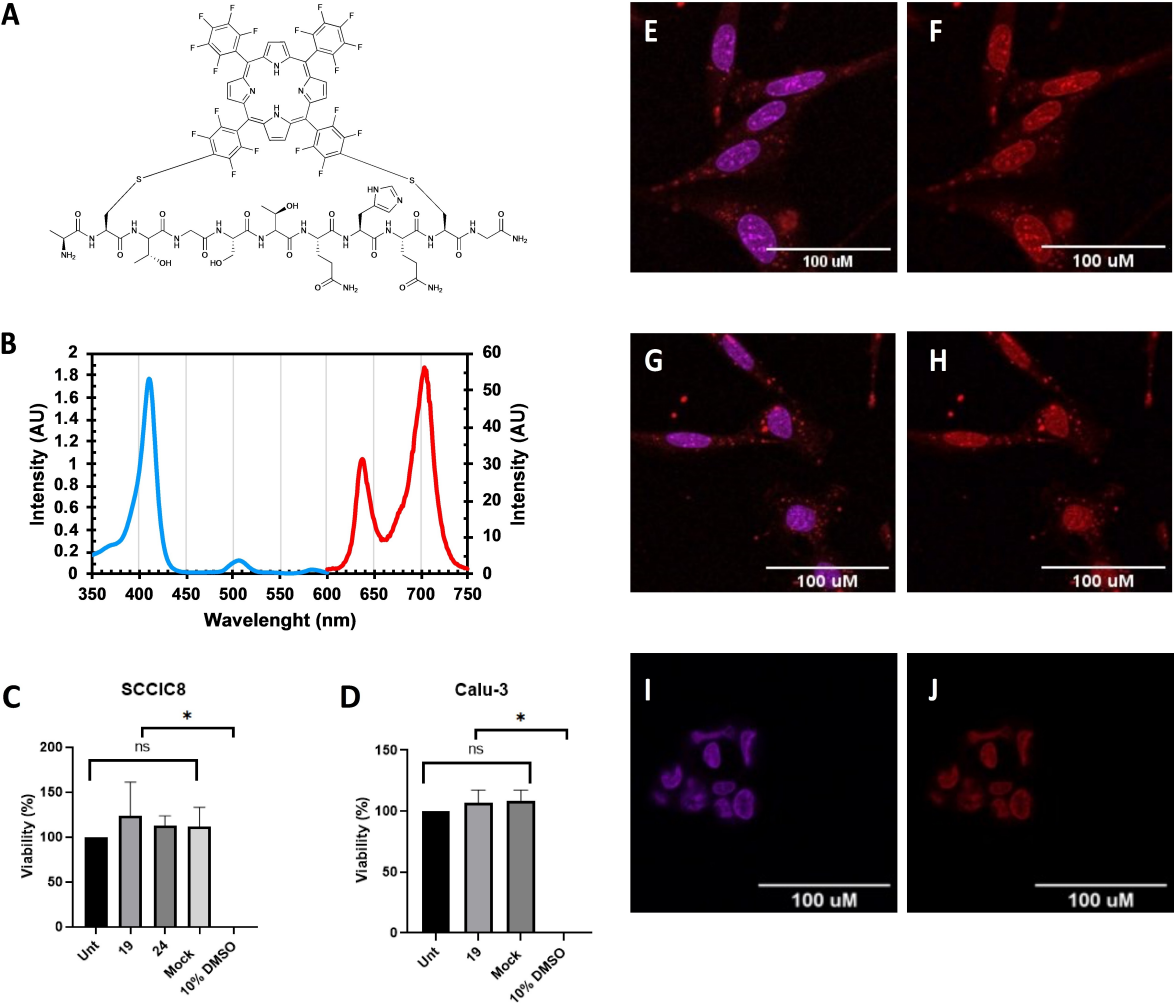
(**A**) Chemical structure of conjugate **19**. (**B**) Absorbance (blue) and emission (red) spectra of **19**. Cell viability (%) of (**C**) SCCIC8 cells and (**D**) Calu 3 cells (Unt=untreated). Intracellular localisation (red fluorescence) of **19** (**E** and **F**) and **29** (**G** and **H**) in SCCIC8 cells. No uptake of **19** in Calu 3 cells (**I** and **J**). Confocal micrographs were acquired with a 63× objective and scale bar is 100 μm. Nuclei are stained with DAPI (blue fluorescence).

Finally, to determine whether the water‐soluble cyclic product **19** (Figure 3A) retained skin cell penetrating properties, as originally reported for the SPACE peptide by Hsu and Chen,[^36^, ^37^] its cellular uptake in squamous cell carcinoma cells (SCCIC8) was tested.^[49]^ Cell imaging by confocal microscopy was facilitated by the intrinsic fluorescence of the porphyrin‐containing cyclic products in the red region (absorption at 545 nm, emission at 600 nm) whose characteristic absorption and emission spectra are shown in Figure 3B. Subsequent analysis of cells after treatment showed significant uptake of cyclic product **19** (Figure 3E and 3F), indicating that the conjugate retained the skin cell penetrating properties of the peptide. The uptake of cyclic product **29** – the cyclised version of the scrambled SPACE peptide control – was not significantly different from cyclic product **19**. This suggests that the uptake was not specifically related to the exact order of the residues (Figure 3G and 3H). Importantly, cyclic product **19** was not taken‐up in to a non‐small‐cell lung cancer cell line (Calu‐3; Figure 3I and 3 J). Thus, it localizes in SCCIC8 cells in a peptide specific manner, and cyclisation with **2** does not preclude peptide uptake. Furthermore, at concentration of 10 μM, **19** did not induce cytotoxicity in SCCIC8 nor Calu‐3 cells (Figure 3C and 3D). Whilst we cannot be certain that this is the 5–10 regioisomer based on our observations, only one stapled product was obtained, and work is ongoing to fully elucidate the selectivity. The ability of the cyclic SPACE‐porphyrin conjugate **19** to permeate skin cells confirmed that the peptide retained its original cell penetrating activity, while porphyrin mediated stapling afforded an intrinsically fluorescent conjugate for microscopy studies.

## Conclusions

Using this robust and chemoselective chemistry, 5,10,15,20‐tetrakis(pentafluorophenyl)porphyrin can be a widely applicable platform for the cyclisation of deprotected cysteine‐containing peptides in solution. This approach is not sequence specific and can be performed with any peptide sequence presenting, naturally or after specific modification, two cysteine at defined distances. This new synthetic approach provides access to a new class of strapped porphyrins, a new platform for peptide stapling and multicyclisation; as well as the ability to combine the functional properties of porphyrins, such as metal‐binding, catalytic and photophysical attributes, with the biomimicry features of peptides.

## Experimental Section

**General information**: All the reagents were purchased from commercial suppliers and used directly as indicated in the appropriate experimental procedures. All Fmoc L‐amino acids with standard side‐chain protecting groups (Fmoc‐Ala‐OH, Fmoc‐Cys(Trt)‐OH, Fmoc‐Gly‐OH, Fmoc‐His(Trt)‐OH, Fmoc‐Leu‐OH, Fmoc‐Gln(Trt)‐OH, Fmoc‐Ser(tBu)‐OH, Fmoc‐Thr(tBu)‐OH, Fmoc‐Tyr(tBu)‐OH, Fmoc‐Asn(Trt)‐OH, Fmoc‐Val‐OH, Fmoc‐Ile‐OH, Fmoc‐Glu(tBu)‐OH, Fmoc‐Arg(Pbf)‐OH, Fmoc‐Lys(Boc)‐OH, Fmoc‐Pro‐OH), Rink Amide Pro‐Tide resin and Oxyma Pure were purchased from CEM UK Ltd (Buckingham, UK). Trifluoroacetic acid (TFA, HPLC grade), *N*,*N*′‐Diisopropylcarbodiimide (DIC), 2,3,4,5,6‐pentafluorobenzaldehyde and silica gel were purchased from Fluorochem UK Ltd (Hadfield, UK). Acetonitrile (MeCN, both HPLC and MS grade), formic acid (MS grade), dimethylformamide (DMF), boron trifluoride etherate (BF_3_ ⋅ Et_2_O), chloranil, petroleum ether, ethyl acetate (EtOAc), methanol (MeOH) and dichloromethane (CH_2_Cl_2_) were purchased from Fisher Scientific UK Ltd (Loughborough, UK). *N*,*N*′‐Diisopropylethylamine (DIPEA), triethylamine (Et_3_N), piperidine, hexafluorobenzene (HFB), triisopropylsilane (TIPS), sodium sulfate anhydrous (Na_2_SO_4_), sodium bicarbonate (NaHCO_3_) dry dimethyl sulfoxide (DMSO), cesium carbonate (Cs_2_CO_3_), sodium hydroxide (NaOH), sodium chloride (NaCl), diethyl ether (Et_2_O), tris(2‐carboxyethyl)phosphine (TCEP), pyridine, deuterated chloroform (CDCl_3_) and pyrrole were purchased from Sigma Aldrich Company Ltd (Gillingham, UK). Pre‐packed RediSep C18 Gold columns (5.5 mg) were purchased from Teledyne ISCO (Lincoln, NE, USA). Gibco Dulbecco's Modified Eagle Medium (DMEM), Minimum Eagle Media (MEM), New Born Calf Serum (NBCS), Fetal Bovine Serum (FBS), and TrypLE Express Enzyme (1×) without phenol red were purchased from Thermofisher UK Ltd (Runcorn, UK). The 4′,6‐diamidino‐2‐phenylindole (DAPI) containing fluoromount G mounting medium was also purchased from Thermofisher UK Ltd. The resazurin sodium salt was purchased from Sigma Aldrich Company Ltd (Gillingham, UK). ^19^F NMR (proton decoupled, 512 scans) spectra were recorded on a Bruker AVA400, operating at a transmitter frequency of 386 MHz. Samples were dissolved in methanol‐*d*_4_ and transferred in high‐resolution NMR tubes. Data analysis was carried out using TopSpin 3.1, Bruker UK Ltd (Coventry, UK).

**Peptide synthesis**: Each linear peptide sequence was prepared using automated Fmoc‐solid phase peptide synthesis methods on a CEM Liberty Blue microwave‐assisted peptide synthesizer. Solid‐phase synthesis was conducted on a 0.1 mmol scale using Rink amide Pro‐Tide resin (179 mg, 0.56 mmol/g loading), employing the required Fmoc‐L‐amino acids (0.2 M in DMF, 5 equiv.), DIC (1 M in DMF; 10 equiv.) as activator, Oxyma Pure (1 M in DMF, 5 equiv.) as racemisation suppressor, and piperidine (20 % v/v in DMF; 4 mL) as deprotection reagent. Standard coupling procedures employed single coupling of each amino acid (2.5 min, 90 °C) and Fmoc‐deprotection (2 min, 90 °C). Racemisation‐prone amino acids bearing heat‐sensitive protecting groups, for example Fmoc‐Cys(Trt)‐OH, were coupled under milder conditions (10 min, 50 °C). Following on‐resin synthesis of the appropriate sequence, the resin was transferred to 10 mL syringes with frits and shrank by washing with Et_2_O. Finally, peptides were cleaved from the resin as the C‐terminal amide by treatment with a cleavage cocktail of TFA, TIPS and H_2_O (8 : 1 : 1 v/v) with shaking at room temperature for 4 h. Peptides were precipitated from cleavage solutions by dropwise addition into cold Et_2_O followed by centrifugation. The resulting pellet was successively suspended in cold Et_2_O, vortexed and centrifuged twice further. The solids obtained after Et_2_O removal and its complete evaporation were dissolved in H_2_O, MeCN or MeOH with 0.1 % trifluoroacetic acid *v*/*v* (or a mixture of these solvents, depending on the solubility) and analysed by HPLC and LC–MS as described below. Peptides were used as crude for successive reactions without further chromatographic purification.

Peptide cyclisation, general method*: The peptide‐porphyrin cyclisation was performed according to the following general procedure. The solid reagents – crude peptide (0.01 mmol, 1 equiv.), porphyrin (or peptide‐porphyrin conjugate) (9.75 mg, 0.01 mmol, 1 equiv.), Cs_2_CO_3_ (26 mg, 0.08 mmol, 8 equiv.), and TCEP (11.5 mg, 0.04 mmol, 4 equiv.) – were combined in a 5 mL glass vial with lid and rubber septum. The reaction atmosphere was conditioned with N_2_ flow for a few minutes before adding dry DMSO (3 mL). After stirring the mixture at room temperature for 2 h, the reaction was quenched by addition of TFA (half DMSO volume). Conjugation products were precipitated from the solution by addition of Et_2_O followed by centrifugation. The resulting pellet was successively suspended in Et_2_O and centrifuged twice further for complete porphyrin removal. The solids obtained after drying in air were dissolved in H_2_O and/or MeCN with 0.1 % trifluoroacetic acid *v*/*v* (or a mixture of these solvents, depending on the solubility) and analysed by HPLC and LC–MS as described below. * Note some exceptions:

- 5 h reaction time was employed for synthesis of peptides **45–47**.
- 5 h reaction time and more dilute reaction (5 mL DMSO/0.01 mmol of **3**) were employed for reactions with **3**.
- 2 equiv. peptide **9** were used to directly obtain bicyclic product **40**.
- 2 equiv. peptide **4**, **9**, **10** were used to obtain bicyclic peptides **40**–**42** by reaction with conjugate **24**.

**Peptide analysis and purification**: LC–MS characterisation of peptides and peptide‐porphyrin conjugates were conducted using a Waters Micromass LCT Premiere Mass Spectrometer equipped with an Agilent Poroshell 120 EC‐C18 column (2.7 μm, 3.0×50 mm) from Agilent Technologies UK Limited (Cheadle, UK), with a binary eluent system comprising H_2_O and MeCN as mobile phases, with 0.1 % formic acid. The method settings were: gradient from 90 % H_2_O to 100 % MeCN over 10 min, 0.6 mL/min, 2 μL injection, 25 °C, detection at 215 nm. Electrospray ionisation mass spectrometry was conducted in positive ion mode (*m*/*z* range: 600–1700) using a cone voltage of 35 V, desolvation temperature of 300 °C and source temperature of 100 °C. HPLC analysis was conducted using an Agilent 1100 Series HPLC with a binary eluent system comprising H_2_O and MeCN as mobile phases, with 0.1 % trifluoroacetic acid. For peptide characterisation, Phenomenex Gemini‐NX C18 column (5 μm, 110 A, 150×4.60 mm) was employed and the method settings were: gradient from 99 % H_2_O to 100 % MeCN over 20 min, 0.8 mL/min, 10 μL injection, 25 °C, detection at 215 nm. For peptide‐porphyrin conjugates characterization, Waters X‐Bridge Peptide BEH C18 column (130 A, 5 μm, 4.6×150 mm) was used and the method settings were: gradient from 90 % H_2_O to 100 % MeCN over 20 min plus 4 min isocratic at 100 % MeCN, 0.8 mL/min, 10 μL injection, 25 °C, detection at 215 nm and 400 nm. Peptide‐porphyrin conjugates for NMR studies were purified on a scale between 0.01 and 0.02 mmol using a Teledyne ISCO Combiflash NextGen System equipped with a 5.5 mg RediSep Rf Gold C18 Column (20–40 μm) and a binary eluent system comprising H_2_O and MeCN. Peptide‐porphyrin conjugates employed for biological assays were purified using an Agilent 1260 prep‐HPLC equipped with Waters X‐Bridge Peptide BEH C18 OBD Prep Column (130 A, 5 μm, 19×100 mm) and a binary eluent system comprising H_2_O and MeCN as mobile phases, with 0.1 % trifluoroacetic acid. The method settings were: gradient from 70 % H_2_O to 100 % MeCN over 20 min, 8.0 mL/min, 10 μL injection, 25 °C, detection at 215 nm and 400 nm.

**Synthesis of 5,10,15,20‐tetrakis(pentafluorophenyl) porphyrin (2)**: In a 3‐neck round bottom flask, DCM (600 mL) was stirred for 5 min under N_2_ flow. 2,3,4,5,6‐pentafluoro benzaldehyde (2.5 g, 12.7 mmol) and freshly distilled pyrrole (1 mL, 14.4 mmol) were transferred into the flask and the mixture stirred under N_2_ at room temperature for 30 min. BF_3_ ⋅ Et_2_O (0.5 mL, 4 mmol) was added, and the reaction heated to 40 °C and protected from light. After 4 h, chloranil (3.5 g, 14 mmol) was added and the reaction heated to reflux overnight and protected from light. After 18 h, the reaction mixture was cooled to room temperature and washed with a saturated solution of NaHCO_3_ (3×200 mL). The organic phase was dried over anhydrous Na_2_SO_4_, filtered, and evaporated to dryness under reduced pressure. The crude product was then loaded on a silica gel column and filtrated using petroleum ether/CH_2_Cl_2_ (6 : 4). Evaporation of the eluate afforded a purple solid, which was crystallised from CH_2_Cl_2_/MeOH to obtain **2** as purple crystals (1.4 mg, 1.44 mmol, 9 %).

**Synthesis of 5,15‐bis(pentafluorophenyl)porphyrin (3)**: 2,3,4,5,6‐pentafluorobenzaldehyde (1.6 g, 8 mmol) was transferred into an oven‐dried round bottom flask and conditioned with argon flow for a few minutes before the addition of freshly distilled pyrrole (111 mL, 1.6 mol). Finally, TFA (100 μL, 0.8 mmol) was added under magnetic stirring. After 10 min, the reaction was quenched with NaOH (75 mL, 0.2 M). 80 mL EtOAc were added to the reaction mixture and the organic phase washed with H_2_O (3×100 mL) and brine (100 mL), dried over Na_2_SO_4_ and evaporated to dryness under reduced pressure. The obtained yellow oil was loaded onto a silica gel column and separated by flash‐chromatography using EtOAc/CH_2_Cl_2_/Et_3_N (8 : 2 : 1). After evaporation of the eluate, the product was crystallised from hexane to obtain (pentafluorophenyl)dipyrromethane as white solid (1.52 g, 4.9 mmol, 60 %). In a 3‐neck round bottom flask, equipped with a condenser, a dropping funnel, an argon inlet adaptor, and a magnetic stirring bar, (pentafluorophenyl) dipyrromethane (480 mg, 1.54 mmol) was dissolved in CH_2_Cl_2_ (420 mL). Trimethylortoformate (12 mL, 110.8 mmol) was added in one portion under Ar_2_ flow. A solution of TFA (4.5 mL, 58.5 mmol) in CH_2_Cl_2_ (150 mL) was added dropwise over 30 min at room temperature and in the dark. The reaction mixture was stirred at room temperature for 4 h before quenching the reaction with pyridine (16 mL, 0.2 mmol). After 19 h, compressed air was bubbled into the reaction mixture for 15 min and the reaction stirred for 4 h more at room temperature, in the air and light. The mixture was then evaporated to dryness and the crude product was purified by silica gel flash chromatography using CH_2_Cl_2_/petroleum ether (7 : 3) as mobile phase. The eluate was evaporated and the product crystallised from CH_2_Cl_2_/MeOH yielding **3** as dark solid (55 mg, 0.085 mmol, 5.5 %).

**Synthesis of Pd 5,10,15,20‐tetrakis(pentafluorophenyl) porphyrin (33)**: In a round bottom flask, equipped with a condenser, a CaCl_2_ guard tube, and a magnetic stirring bar, tetrakis(pentafluorophenyl)porphyrin (**2**, 200 mg, 0.2 mmol) and PdCl_2_(PhCN)_2_ (230 mg, 0.6 mmol) were dissolved in PhCN (420 mL) under inert atmosphere. The reaction mixture was heated to reflux and stirred at room temperature for 24 h. The mixture was then cooled to room temperature and the product was precipitated by addition of cold MeOH. The crude product was purified using silica gel flash chromatography using CH_2_Cl_2_/PE (7 : 3) as mobile phase. The eluate was evaporated, and the product crystallised from CH_2_Cl_2_/MeOH/H_2_O yielding **33** as red crystals (186 mg, 0.173 mmol, 87 %).

**Cell culture and viability studies**: The SCCIC8 and Calu‐3 cells were maintained in DMEM supplemented with 10 % NBCS and MEM supplemented with 10 % FBS and 1 % Penicillin/Streptomycin, respectively. Cells were passaged once they reached 70–80 % confluency. For cell viability experiments, cells were seeded at 10×10^4^ per well of a 96‐well plate and maintained in complete media for 24 h. Subsequently, the cells were treated with **19** and **24** (10 μM), and the respective controls. The cells were incubated for a further 24 h. After this period the treated culture medium was removed, and cells were washed with 200 μL PBS. The cells were treated with resazurin (0.1 mg/mL, 100 μL) and incubated in the dark at 37 °C for 4 h. Finally, the plate was read on a CLARIOstar plate reader (BMG Labtech, Aylesbury, UK), fluorescence was measure at an excitation wavelength of 545 nm and an emission wavelength of 600 nm. Each experiment was performed in triplicate. The experiments were tested for normality and statistical analysis was carried out using one‐way ANOVA with Dunnett's multiple comparisons test; levels of significance: * p<0.05, compared to untreated control. All statistical tests were performed with GraphPad prism 9.

**Fluorescence microscopy and cell uptake studies**: SCCIC8 and Calu‐3 cells were seeded at 10×10^4^ on glass cover slips in a 6‐well plate for 24 h. Subsequently, cells were treated with **19** and **24** (10 μM) and incubated for a further 24 h. Prior to visualisation culture media was removed, cells were washed with PBS, fixed using ice‐cold MeOH for 7 min at −20 °C, after fixation cells were successively washed with PBS. Cells were mounted with 4’,6‐diamidino‐2‐phenylindole (DAPI) containing fluoromount G mounting medium. Images were collected using a Zeiss LSM 710 Confocal microscope using oil immersion objective 63×. Images of the DAPI stained nucleus were captures using excitation wavelength 405 nm (blue laser), emission wavelengths 410–496 nm and laser power 0.8 %. Whilst the cellular uptake of **19** and **24** was captured using excitation wavelength 405 nm (blue laser) and emission wavelengths 565–695 nm and laser power 5 %.

**Computational Chemistry studies**: For each of the 15 peptides, up to ten conformers were generated from a SMILES string with ETDKGv3^[50]^ and implemented in the RDKit python package.^[51]^ ETDKGv3 was not able to generate any conformer for peptide **18**. Each generated conformer was optimized with XTB[^43^, ^44^] with the keyword ‘ohess’ using the default option for accuracy. Generalised Born model with Solvent Accessible Surface Area for non‐polar interaction (GBSA)^[52]^ for DMSO was used as implicit solvent model. The average of the three‐dimensional Euclidean distance was calculated, for each set of conformers, considering the distance between the two sulfur atoms (S) of the peptide on the XTB‐optimised geometry. The porphyrin **2** structure was generated in a similar fashion with ETDKGv3 and optimised to a minimum with XTB using the same options for the peptides. Two distances between the 4‐position carbon of pentafluorophenyl groups were considered: one between adjacent groups (15–20, 20–5, 5–10, 15–10) and one between opposite groups (10–20, 15–5). The average distances were 10.9 Å for the adjacent groups and 15.5 Å for the opposite groups.

## Supporting Information

Author contributions, supplementary experiments and compound characterization can be found in the Supporting Information Additional references cited within the Supporting Information.[^53^, ^54^, ^55^, ^56^]

## Conflict of interest

The authors declare no conflict of interest.

1

## Supporting information

As a service to our authors and readers, this journal provides supporting information supplied by the authors. Such materials are peer reviewed and may be re‐organized for online delivery, but are not copy‐edited or typeset. Technical support issues arising from supporting information (other than missing files) should be addressed to the authors.

Supporting Information

## Data Availability

The data that support the findings of this study are available in the supplementary material of this article.
