# Supplementary material for: 5,10,15,20‐Tetrakis(pentafluorophenyl)porphyrin as a Functional Platform for Peptide Stapling and Multicyclisation
Source: Chemistry. 2023 Sep 6;29(55):e202301410. doi: 10.1002/chem.202301410 (PMC10946732; doi:10.1002/chem.202301410)
Supplement: Supplementary file 1 — Supporting Information [file CHEM-29-0-s001.pdf]

Chemistry–A European Journal

Supporting Information

5,10,15,20-Tetrakis(pentafluorophenyl)porphyrin as a Functional Platform for Peptide Stapling and Multicyclisation

Paolo Dognini, Talhat Chaudhry, Giulia Scagnetti, Michele Assante, George S. M. Hanson, Kehinde Ross, Francesca Giuntini,* and Christopher R. Coxon*

Author Contributions

PD, FG, CRC, KR, and TC contributed to the conceptualisation and design of the study. PD performed the major investigation, involving methodology, synthesis, purification, characterisation, and data curation. He also wrote the main portion of the manuscript, including figures and tables. TC performed biological experiments and wrote the corresponding section of the manuscript. GS supported the biological experiments. MA performed computational calculations and wrote the corresponding section of the manuscript. GSMH executed the ^{19}F NMR analysis. FG, CRC, and KR supervised the work, improved the first draft as well as the final manuscript with organisation of content, figures and tables prior to submission.

Table of Contents

1. Preliminary studies on porphyrin-peptide conjugation 1
2. Peptide stability and reaction time course 4
3. NMR spectra 7
4. Compound characterisation 10
   Porphyrin 10
   Peptides 11
   Porphyrin-Peptides Conjugates 31

1. Preliminary studies on porphyrin-peptide conjugation

Model reactions: N-acetyl cysteine

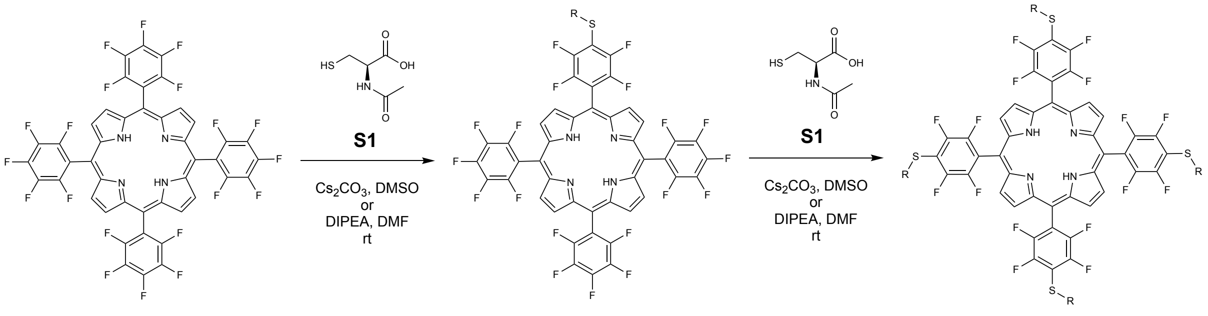

2

R= NAC, **S2**

R= NAC, **S3**

*Figure S1. Reaction scheme of S_NAr between porphyrin **2** and NAC (**S1**) under DMSO/Cs_2CO_3 or DMF/DIPEA conditions.*

The model reaction between 5,10,15,20-tetrakis(pentafluorophenyl)porphyrin (**2**) and N-acetylcysteine (NAC, **S1**) was performed to compare DMSO/Cs_2CO_3 and DMF/DIPEA reaction conditions (Figure S1). These approaches were previously explored using HFB (**1**) and NASC (**S1**).[19] Unreacted porphyrin **2** shows three typical ^{19}F NMR signals with a 2:1:2 pattern (blue in Figure S2). When the monosubstituted product **S2** was formed, two peaks corresponding to the tetrafluorophenyl ring appear at -134 and -139 ppm in addition to the original pattern of unreacted pentafluorophenyl groups (red in Figure S2), while the tetrasubstituted product **S3** contains two pairs of equivalent fluorine nuclei (green in Figure S2). Thiols reacted with the porphyrin under both conditions, but DMSO/Cs_2CO_3 proved to be particularly effective for this chemistry, as the reaction was faster than in DMF/DIPEA. Under

the DMSO/Cs_2CO_3 conditions, the amounts of substituted product formed after 24 h (red in Figure S2) were greater compared with the respective timepoint for DMF/DIPEA (red in Figure S3). With increased time (7 days) and at least 4 equivalents of thiol, complete conversion to tetrasubstituted porphyrin was obtained in DMSO (green in Figure S2), but not in DMF (green in Figure S3).

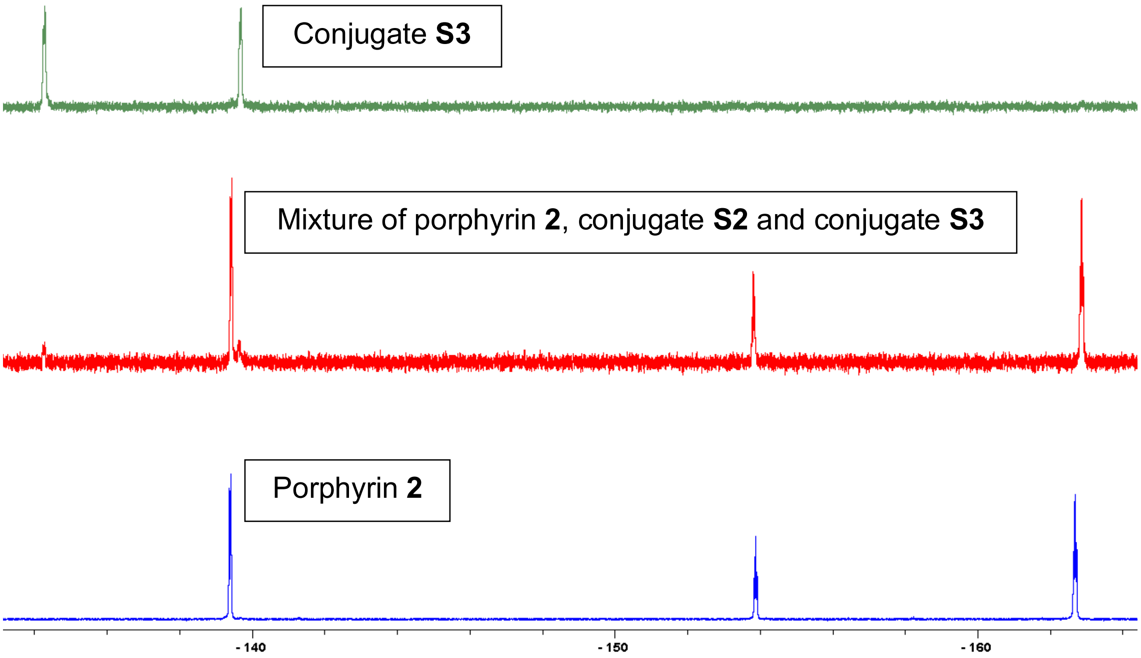

*Figure S2. Monitoring of model reaction between porphyrin **2** and NAC (**S1**) in DMSO with ^{19}F NMR. Spectrum of starting porphyrin in blue, reaction mixture with 1 eq of peptide after 24h in red and reaction mixture with excess of peptide after 7 days in green.*

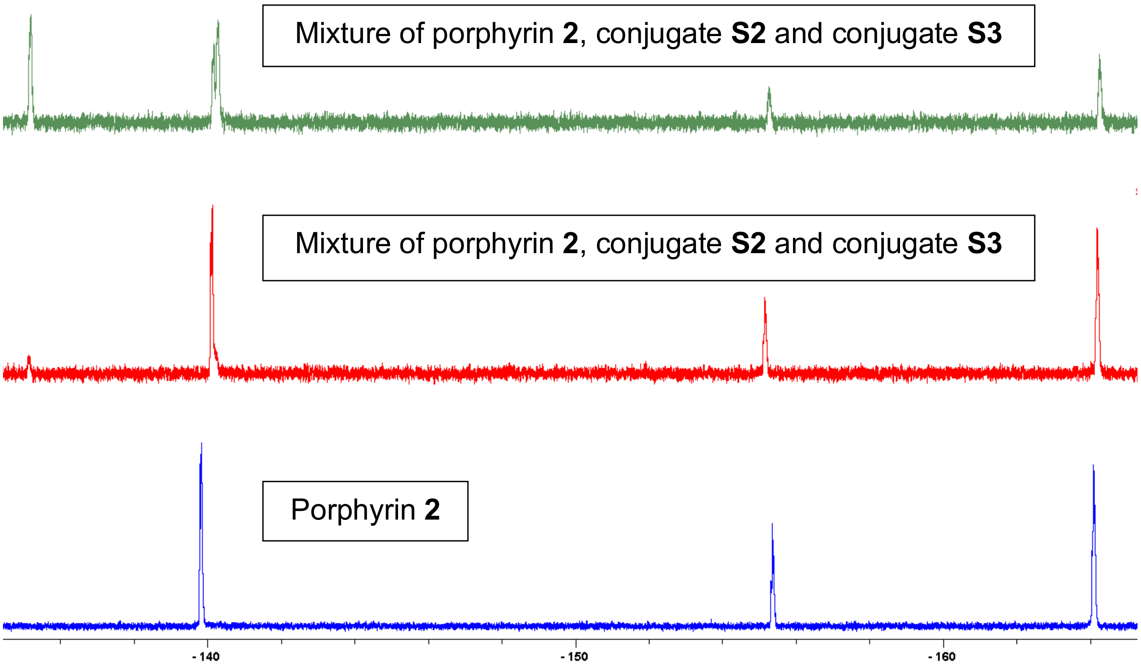

*Figure S3. Monitoring of model reaction between porphyrin **2** and NAC (**S1**) in DMF with ^{19}F NMR. Spectrum of starting porphyrin in blue, reaction mixture with 1 eq of peptide after 24h in red and reaction mixture with excess of peptide after 7 days in green.*

Model reactions: mono-cysteine peptide

The reaction between porphyrin **2** and a single cysteine-containing peptide was also performed (Figure S4), to further compare the difference between these two sets of conditions on a model peptide system, and monitored by HPLC and LC-MS. When 1 eq. of peptide CAAAK (**S4**) was reacted with 2 eq. of porphyrin **2** in DMSO/Cs_2CO_3, double- (**S6**), tri- (**S7**) and tetra-substituted (**S8**) products were formed after 15 min (Figure S5), while in DMF/DIPEA only the mono-substituted (**S5**) product was observed (Figure S6). The double-substituted product (**S6**) was obtained in DMF only after adding an excess of peptide and after 5h reaction time (Figure S7). The DMSO/Cs_2CO_3 condition was, therefore, chosen for peptide-porphyrin cyclisation.

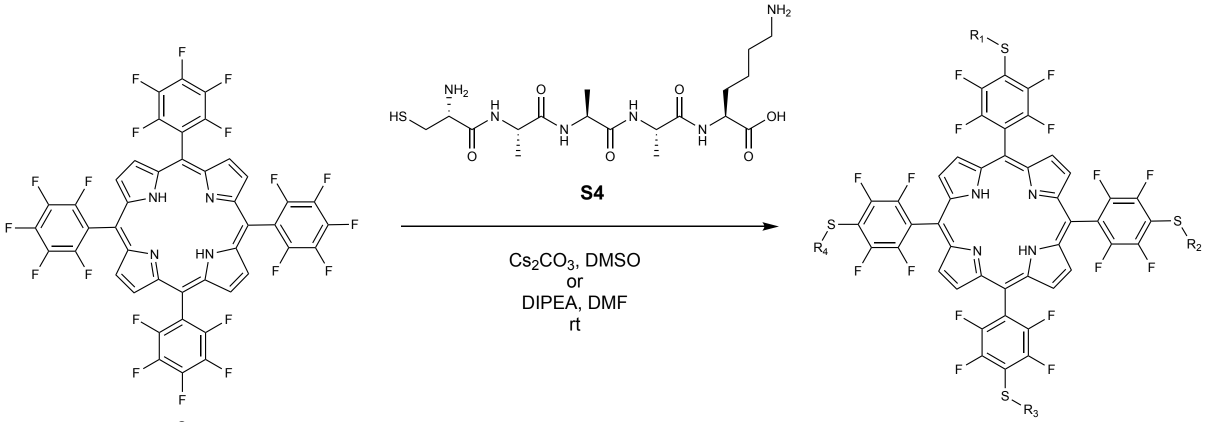

2

R_1= CAAAK, R_2= R_3= R_4= H, **S5**
R_1= R_2= CAAAK, R_3= R_4= H, **S6**
R_1= R_3= CAAAK, R_2= R_4= H, **S7**
R_1= R_2= R_3= CAAAK, R_4= H, **S8**
R_1= R_2= R_3= R_4= CAAAK **S9**

*Figure S4. Reaction scheme of S_NAr between porphyrin **2** and peptide **S4** (CAAK) under DMSO/Cs_2CO_3 or DMF/DIPEA conditions.*

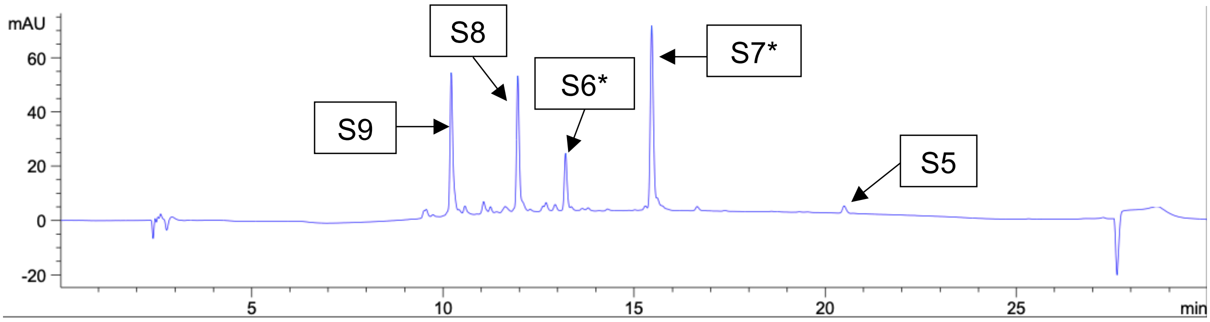

*Figure S5. HPLC chromatogram showing multiple substitution of porphyrin **2** with peptide **4** in DMSO after 15 min. Refer to figure S4 for compound numbers. *No experimental evidence was gathered by the authors to distinguish between **S6** and **S7**, but presumed assignments were based on Wu et al.[25]*

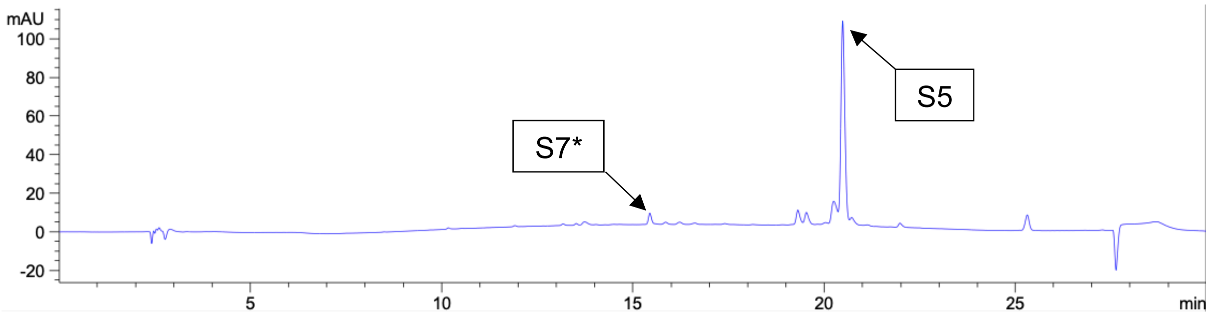

*Figure S6. HPLC chromatogram showing mono substitution of porphyrin **2** with peptide **4** in DMF after 15 min. Refer to figure S4 for compound numbers. *No experimental evidence was gathered by the authors to distinguish between **S6** and **S7**, but presumed assignments were based on Wu et al.[25]*

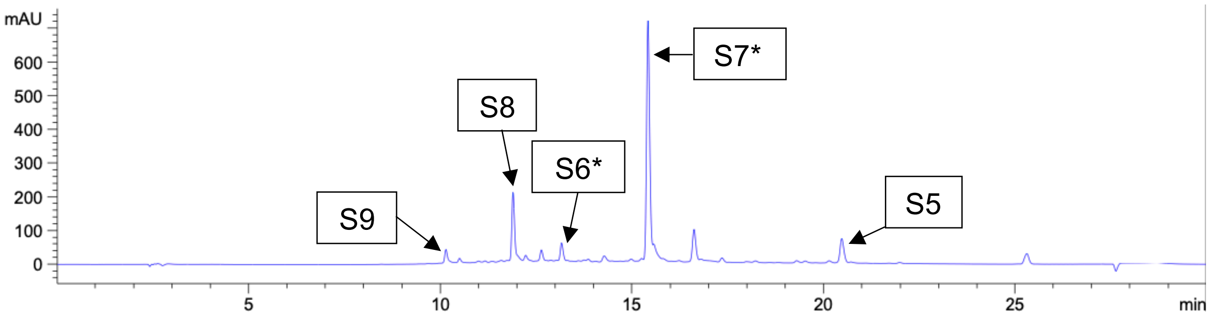

*Figure S7. HPLC chromatogram showing multiple substitution of porphyrin **2** with peptide **4** in DMF, when in excess of peptide and after 5h. Refer to figure S4 for compound numbers. *No experimental evidence was gathered by the authors to distinguish between **S6** and **S7**, but presumed assignments were based on Wu et al.[25]*

2. Peptide stability and reaction time course

The stability of previously purified SPACE peptide (**4**) under reaction conditions (Figure S8) was monitored by sampling a reaction mixture without the porphyrin substrate, obtained following the procedure presented before. The samples were processed and analysed using HPLC (Method C with detection at 215 nm) and LC-MS, as previously described. Probably because of the slow solubilisation of TCEP, the peptide was initially oxidised to its disulfide form. However, the disulfide formation is reversed with time and after 3 hours the peptide is present and mostly unaffected.

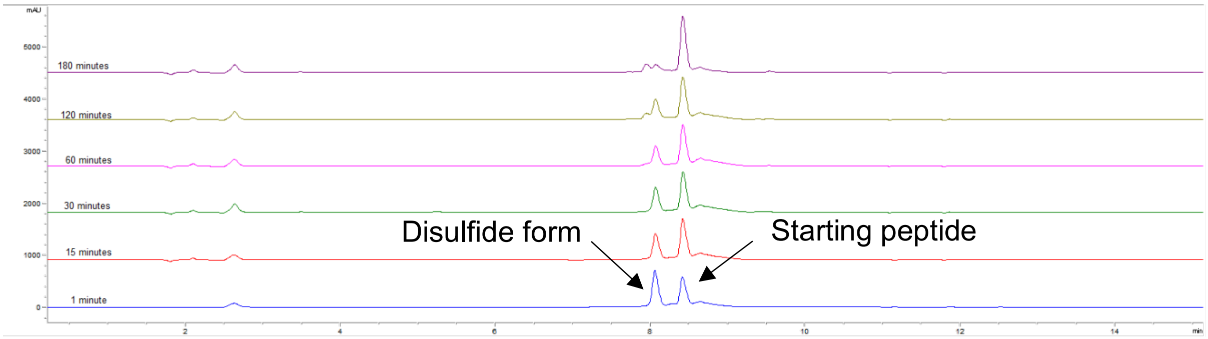

Figure S8. Peptide stability at 215 nm. t1=1 min (blue), t2=15 min (red), t3= 30 min (green), t4= 1 h (magenta), t5= 2 h (olive) and t6= 3 h (purple).

Similarly, the reaction time course was monitored by HPLC (Method C with detection at 215 nm and 400 nm) and LC-MS analysis of samples from a complete reaction mixture obtained following the procedure described earlier. This experiment showed peptide consumption (Figure S9) and porphyrin-derived product formation (Figure S10). When the porphyrin substrate is present, the peptide reacts quickly with main cyclic product formation detectable after 15-30 min. After 2 hours, the amount of peptide left is negligible with no significant changes in crude product composition between the 3 hours timepoint.

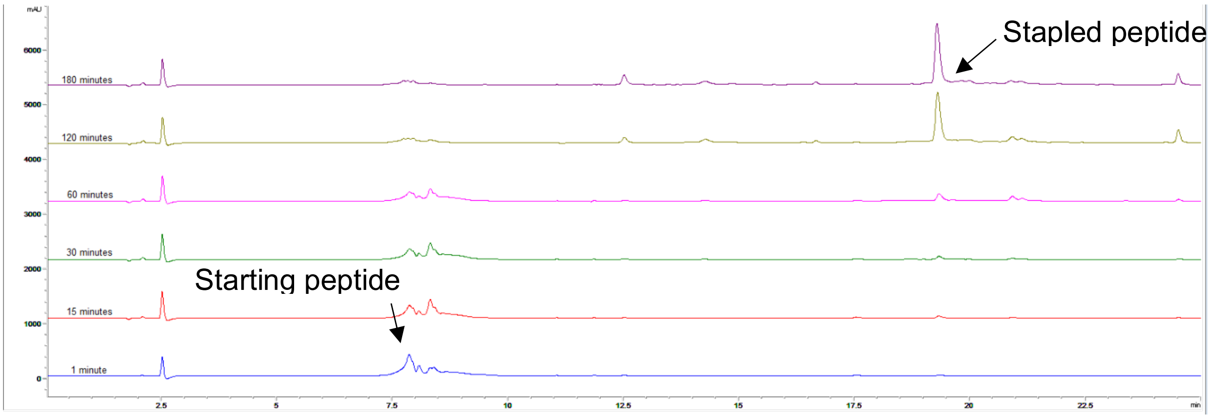

*Figure S9. Reaction time course between porphyrin **2** and peptide **4** (1:1) at 215 nm. t1=1 min (blue), t2=15 min (red), t3= 30 min (green), t4= 1 h (magenta), t5= 2 h (olive) and t6= 3 h (purple).*

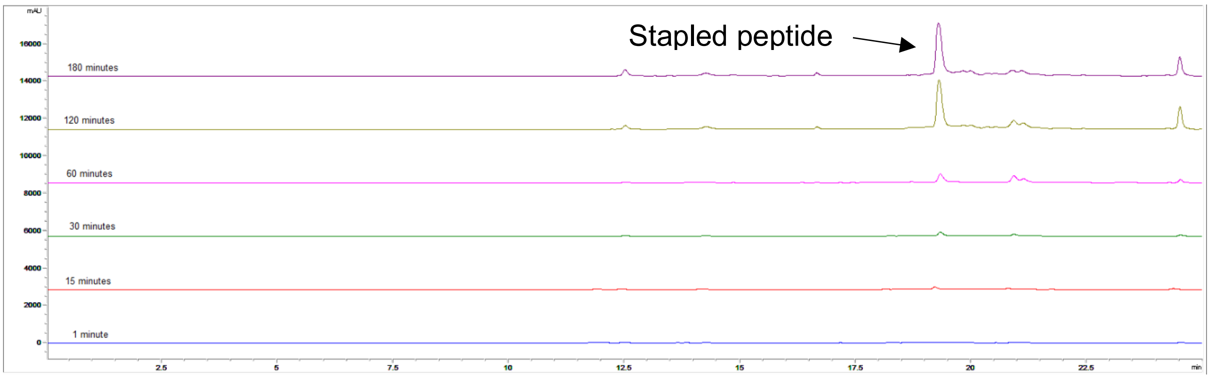

Figure S10. Reaction time course at 400 nm. t1=1 min (blue), t2=15 min (red), t3= 30 min (green), t4= 1 h (magenta), t5= 2 h (olive) and t6= 3 h (purple).

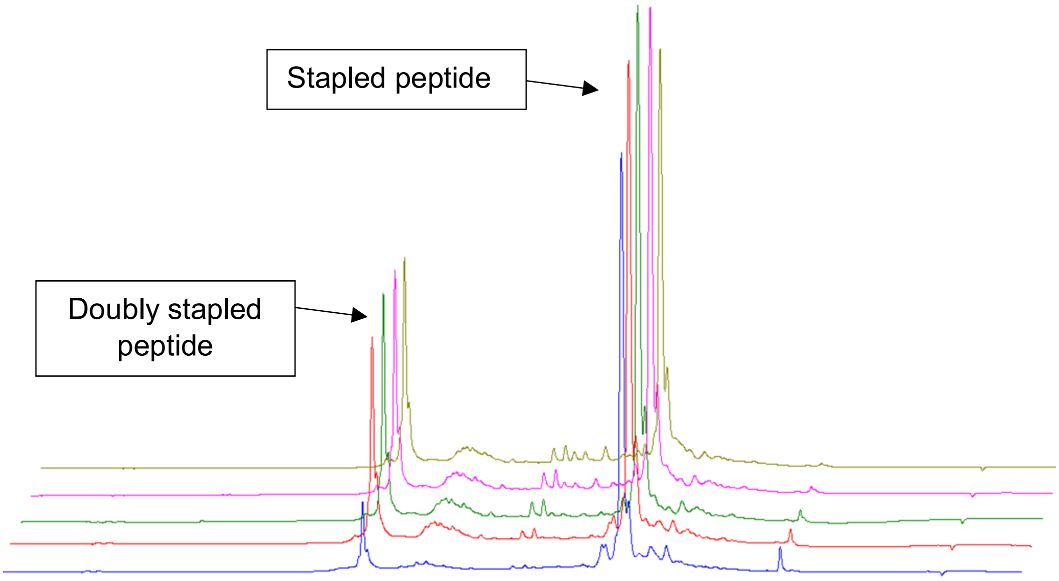

*Figure S11. Reaction time course between porphyrin **2** and peptide **9** (1:2) at 400 nm. t1=1 min (blue), t2=15 min (red), t3= 30 min (green), t4= 1 h (magenta), t5= 2 h (olive) and t6= 3 h (purple).*

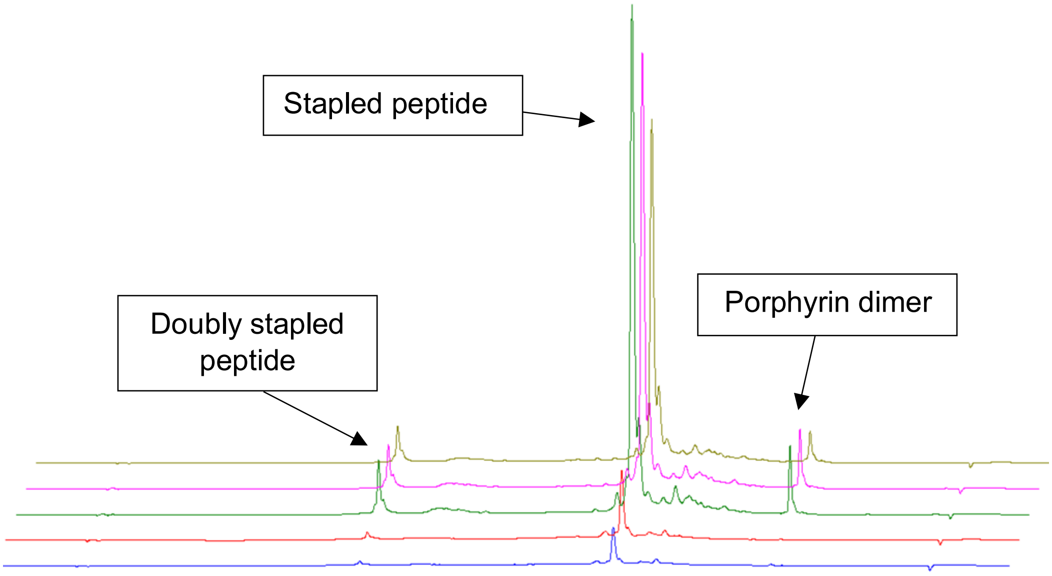

*Figure S12. Reaction time course between porphyrin **2** and peptide **9** (2:1) at 400 nm. t1=1 min (blue), t2=15 min (red), t3= 30 min (green), t4= 1 h (magenta), t5= 2 h (olive) and t6= 3 h (purple).*

3. NMR spectra

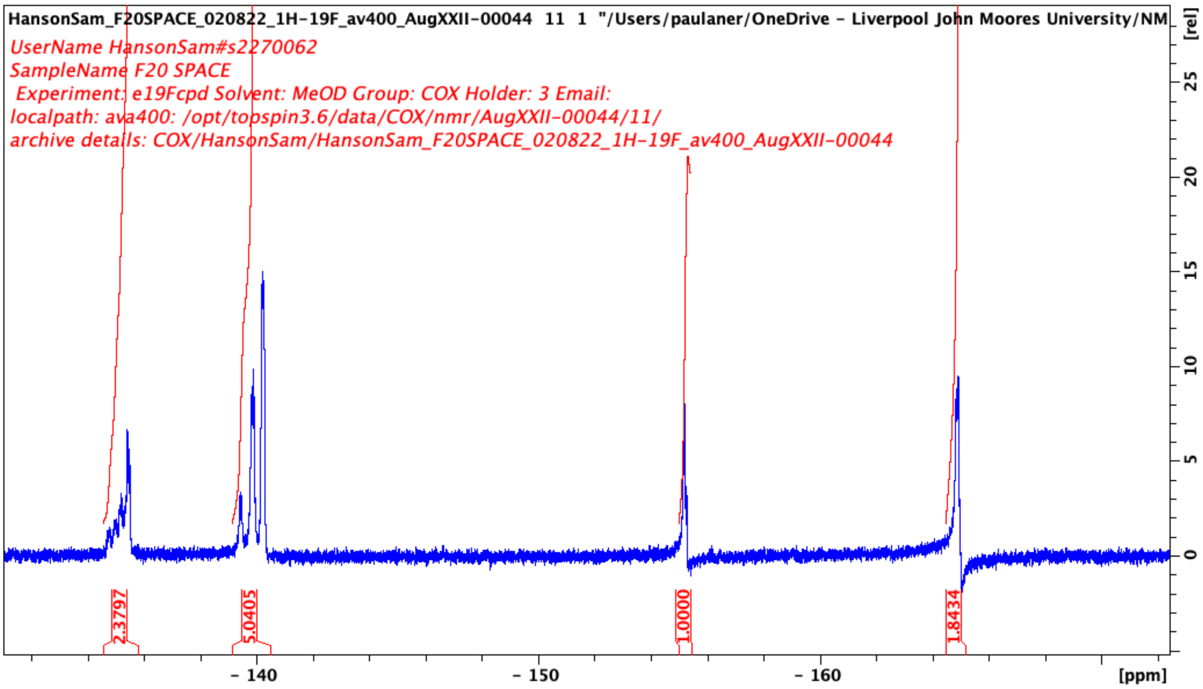

*Figure S13. ^{19}F NMR of stapled product **19**.*

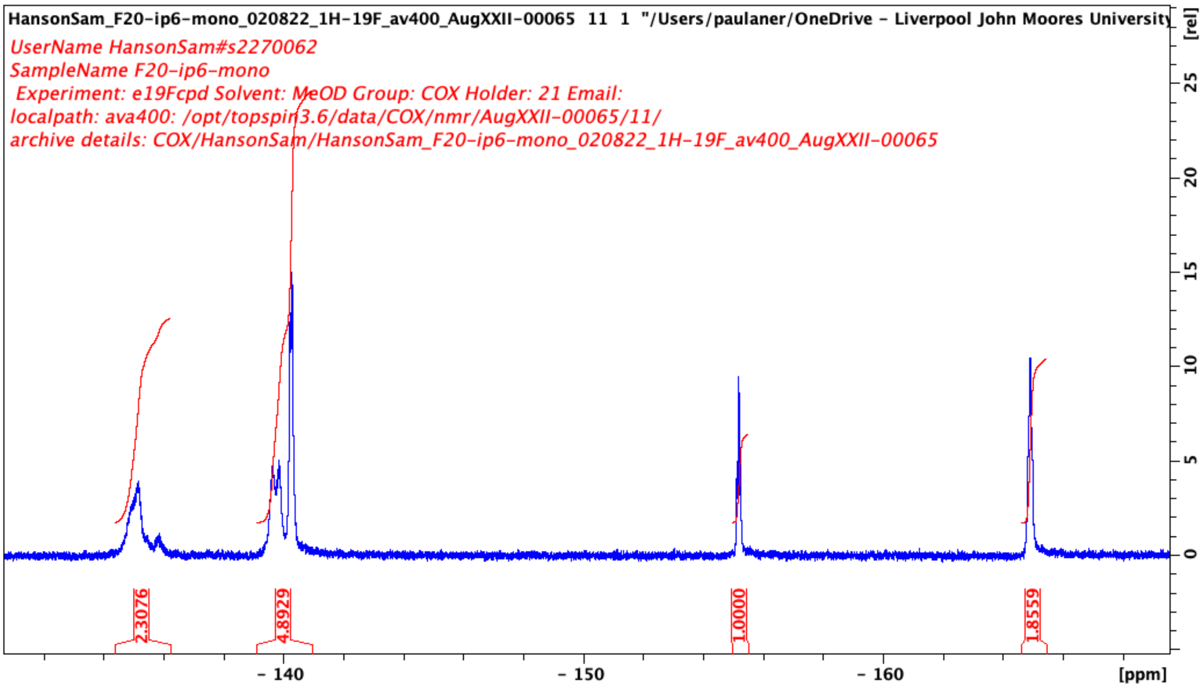

*Figure S14. ^{19}F NMR of stapled product **23**.*

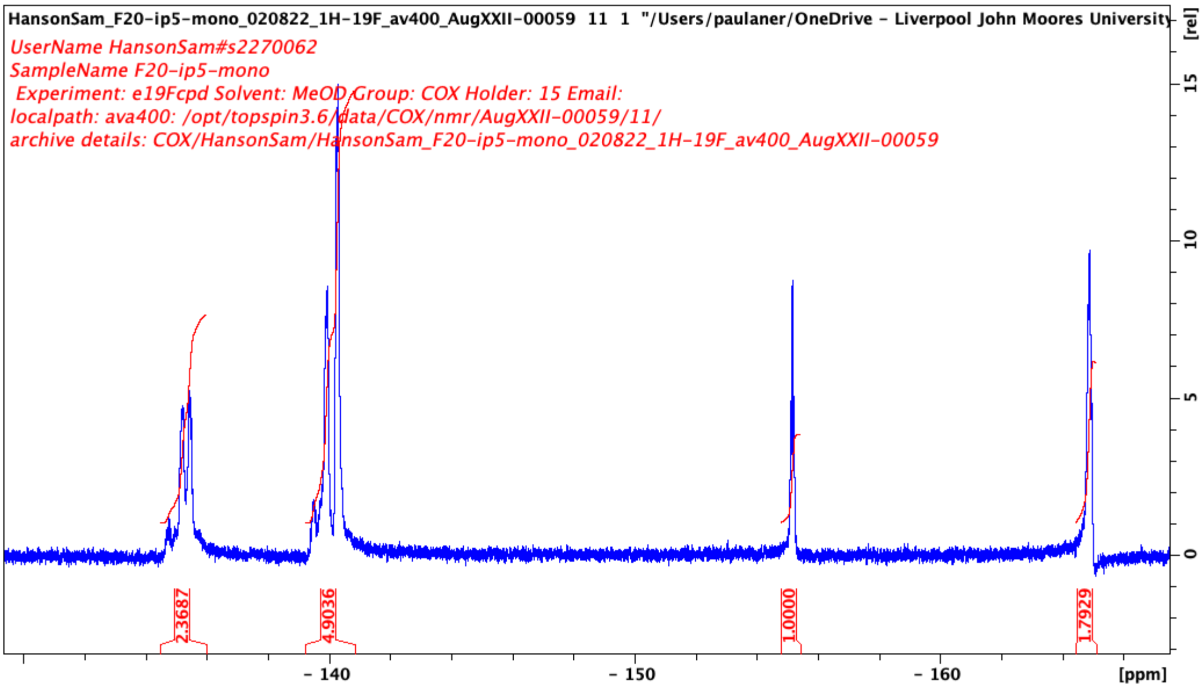

*Figure S15. ^{19}F NMR of stapled product **24**.*

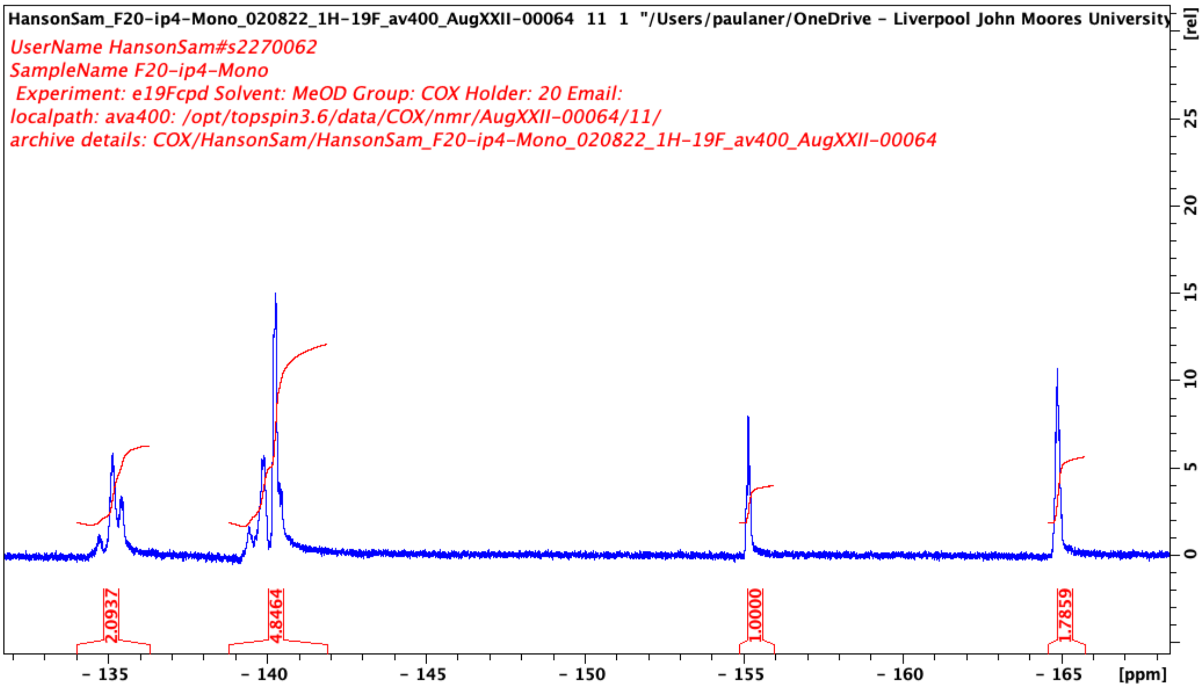

*Figure S16. ^{19}F NMR of stapled product **25**.*

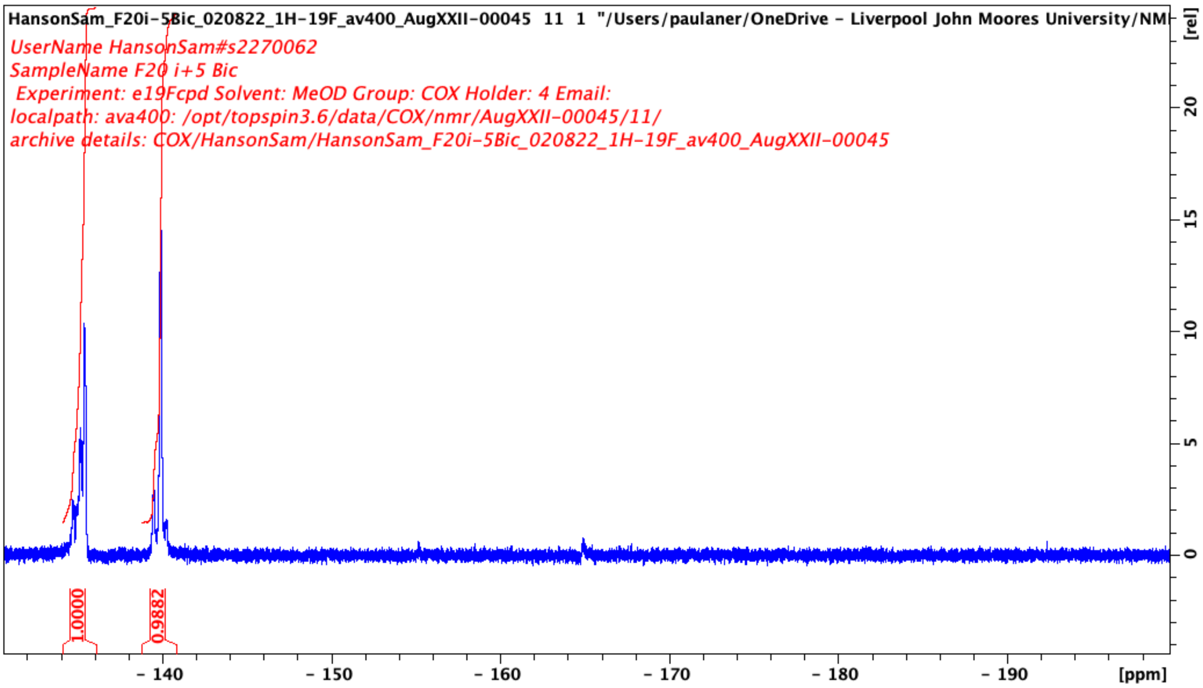

Figure S17. ^{19}F NMR of bicyclic product ***42****.*

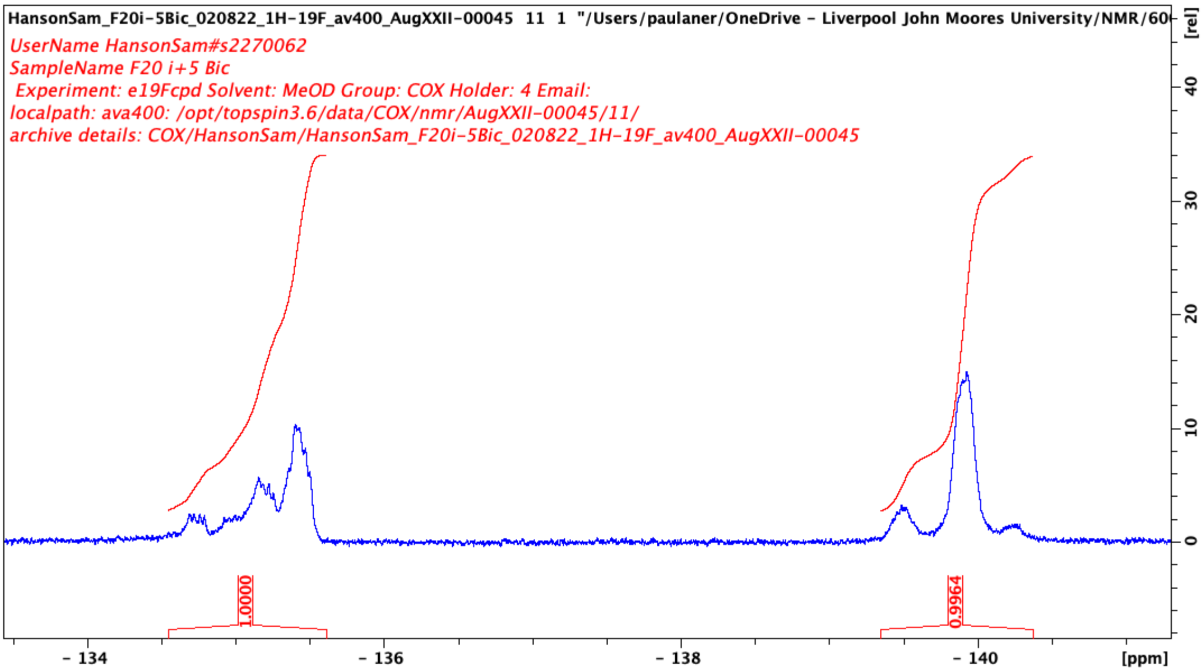

Figure S18. ^{19}F NMR of bicyclic product ***42****.*

4. Compound characterisation

Porphyrins

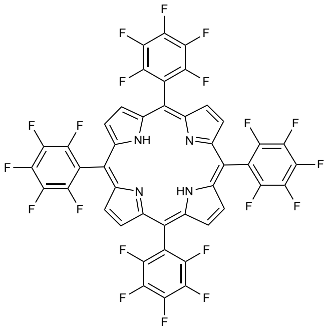

2

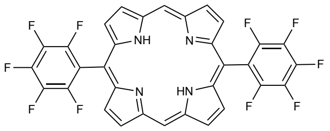

3

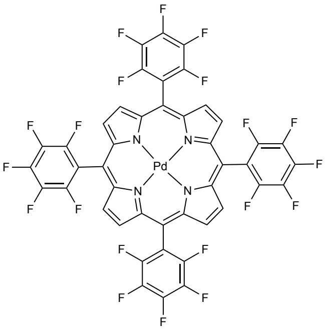

33

Tetrakis(pentafluorophenyl)porphyrin (2): **^{1}H NMR** ($CDCl_3$) δ, ppm: 8.92 (s, 8H, β-H), -2.90 (s, 2H, NH_{int}); **^{13}C NMR** ($CDCl_3$) δ, ppm: 147.36, 145.69, 138.44, 136.76, 115.53, 103.66; **^{19}F NMR** ($CDCl_3$) δ, ppm: -136.55 (dd, J_o = 7 Hz, J_p = 25 Hz, F_o) -151.26 (t, 4F, J_o = 20.5 Hz, F_p), -161.30 (dt, 8F, J_p = 8 Hz, J_o = 23 Hz, F_m); **m.p.** (°C): > 300 **UV-Vis** (MeOH, nm) λ_{max} (Soret): 408; Q bands: 505, 585; **ESI-MS** +v/e (*m/z*): 975.065 $[M+H]^+$. This characterisation is in agreement with previous literature data.[53]

(Pentafluorophenyl)dipyrromethane: **^{1}H NMR** ($CDCl_3$) δ, ppm: 5.89 (s, 1 H), 6.02 (br. s, 2 H), 6.16 (q, J = 3 Hz, 2 H), 6.73 (m, 2 H), 8.14 (br. s, 2 H); **^{13}C NMR** ($CDCl_3$) δ, ppm: 33.09, 107.65, 108.71, 118.10, 128.11; **^{19}F NMR** ($CDCl_3$) δ, ppm: -141.47 (s, 2 F), -155.75 (t, J = 21 Hz, 1 F), -161.20 (dt, J = 7.5, J =21.5, 2 F); **m.p.** (°C): 105 **ESI-MS** +v/e (*m/z*): 242.366 $[M+H]^+$

Bis(pentafluorophenyl)porphyrin (3): **^{1}H NMR** ($CDCl_3$) δ, ppm: 10.39 (s, 2H), 9.49 (d, J = 4.5 Hz, 4H), 9.00 (d, J = 4 Hz, 4H), -3.25 (s, 2H); **^{19}F NMR** ($CDCl_3$) δ, ppm: -136.65 (dd, J_o = 8 Hz, J_p = 24 Hz, F_o) -152.26 (t, 4F, J_o = 21 Hz, F_p), -161.84 (dt, 8F, J_p = 8.5 Hz, J_o = 22 Hz, F_m); **m.p.** (°C): > 300 **UV-Vis** (CH_2Cl_2, nm) λ_{max} (Soret): 403; Q bands: 500, 533, 575, 629; **ESI-MS** +v/e (*m/z*): 643.387 $[M+H]^+$. These characterisations are in agreement with previous literature data. [54,55]

Pd Tetrakis(pentafluorophenyl)porphyrin (33): **^{1}H NMR** ($CDCl_3$) δ, ppm: 8.88 (s, 8H, β-H); **^{13}C NMR** ($CDCl_3$) δ, ppm: 29.7, 105.55, 115.22, 131.26, 141.83; **^{19}F NMR** ($CDCl_3$) δ, ppm: -136.41 (dd, J_o =7.5 Hz, J_p =23.5 Hz, F_o), -151.18 (t, 4F, J_o =21 Hz, F_p), -161.22 (dt, 8F, J_p =7.5 Hz, J_o =22.5 Hz, F_m); **m.p.** (°C): > 300 **UV-Vis** (MeOH, nm) λ_{max} (Soret): 405; Q bands: 518, 551; **ESI-MS** -v/e (*m/z*): 1113.5883 $[M+Cl]^-$. This characterisation is in agreement with previous literature data.[56]

Peptides
Peptide 4

AlaCysThrGlySerThrGlnHisGlnCysGly-NH_2 – Calculated Exact mass: 1090.4284 – Molecular Weight: 1091.1840 – RT: 7.1 min – ESI-MS (*m/z*): 1091.8402 $[M+H]^+$, 546.3800 $[M+2H]^{2+}$

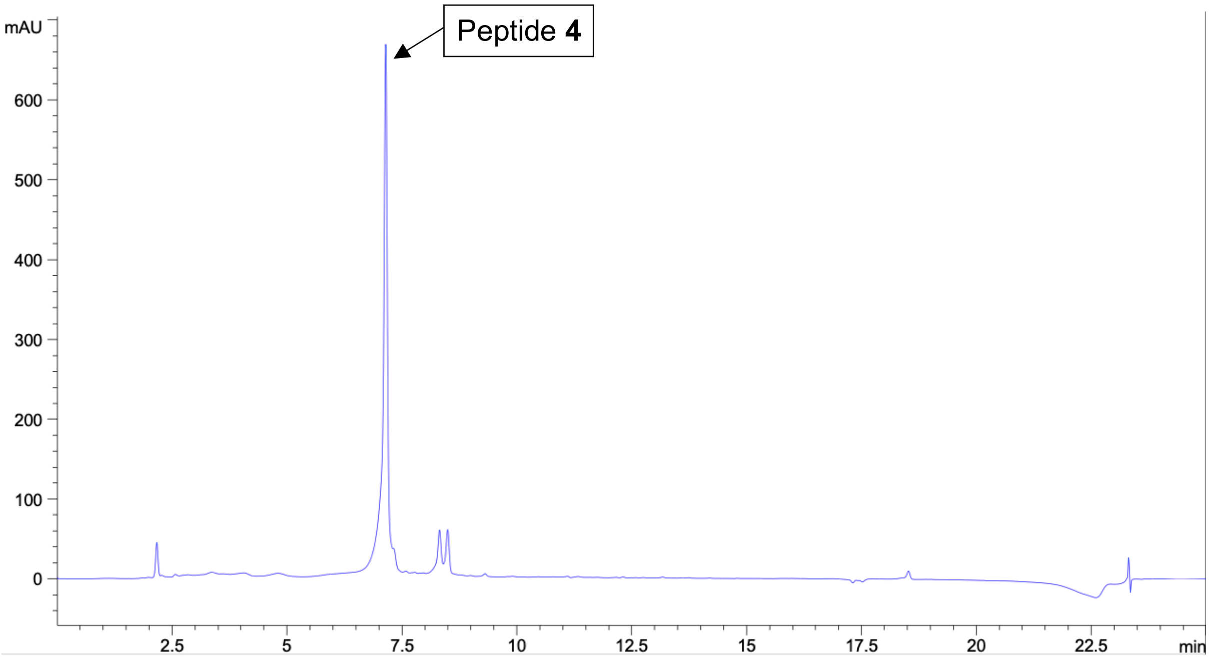

*Figure S19. Analytical HPLC of crude peptide **4**.*

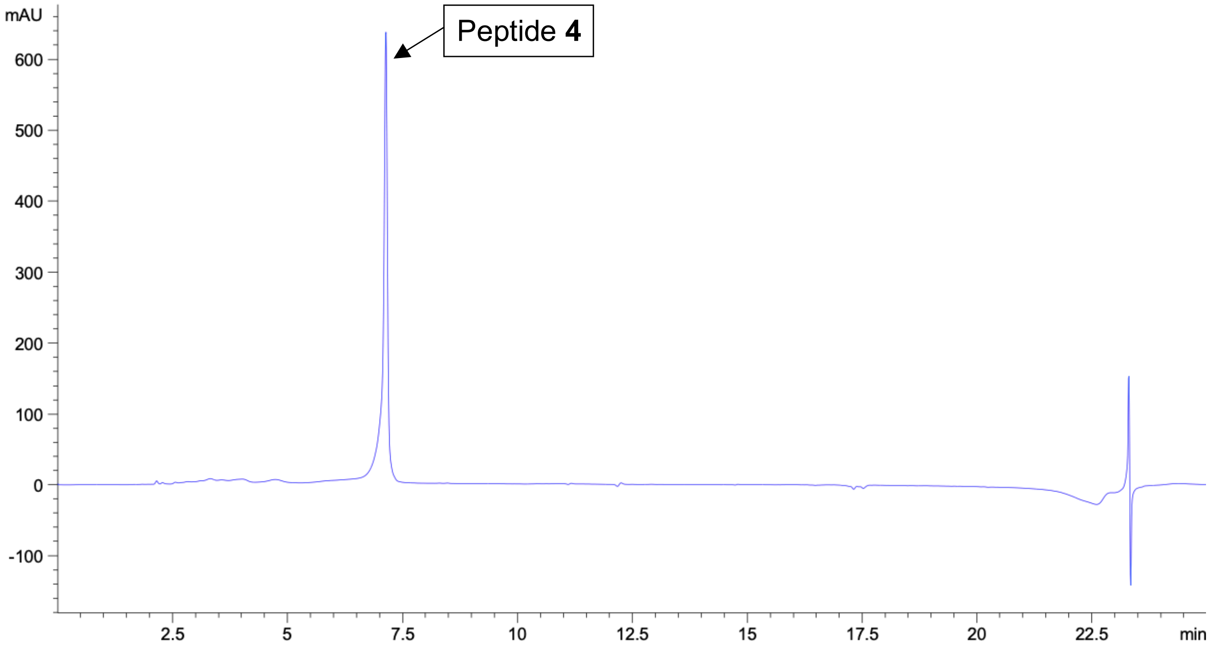

*Figure S20. Analytical HPLC of purified peptide **4**.*

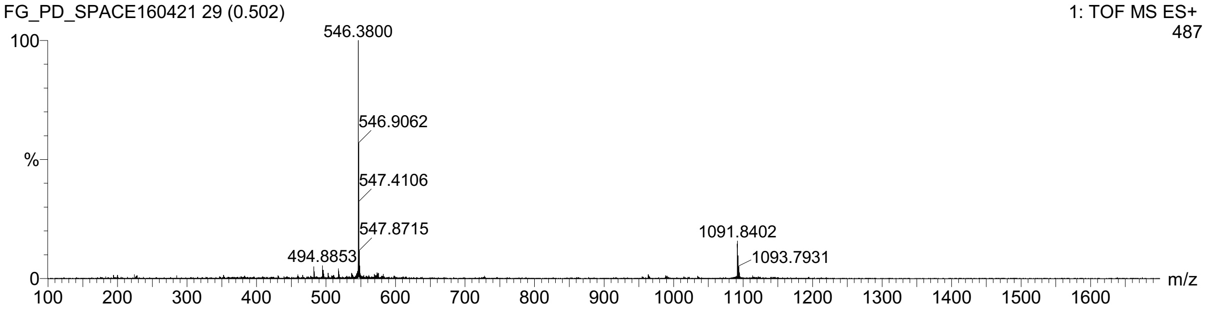

*Figure S21. TIC from TOF MS ES+ analysis of crude peptide **4**.*

Peptide 5

AlaCysAlaThrGlySerThrGlnHisGlnAlaCysGly-NH_2 – Calculated Exact mass: 1232.5026 – Molecular Weight: 1233.3420 – RT: 7.6 min – ESI-MS (*m/z*): 1234.0126 $[M+H]^+$, 617.5123 $[M+2H]^{2+}$, 412.0273 $[M+3H]^{3+}$

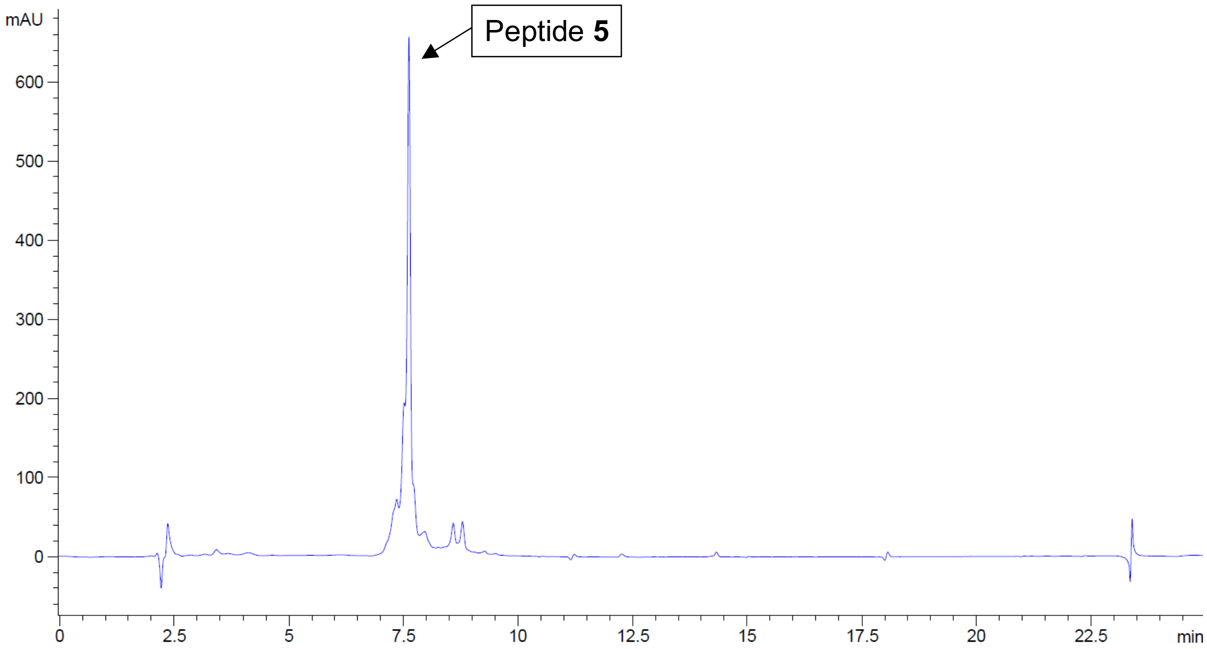

*Figure S22. Analytical HPLC of crude peptide **5**.*

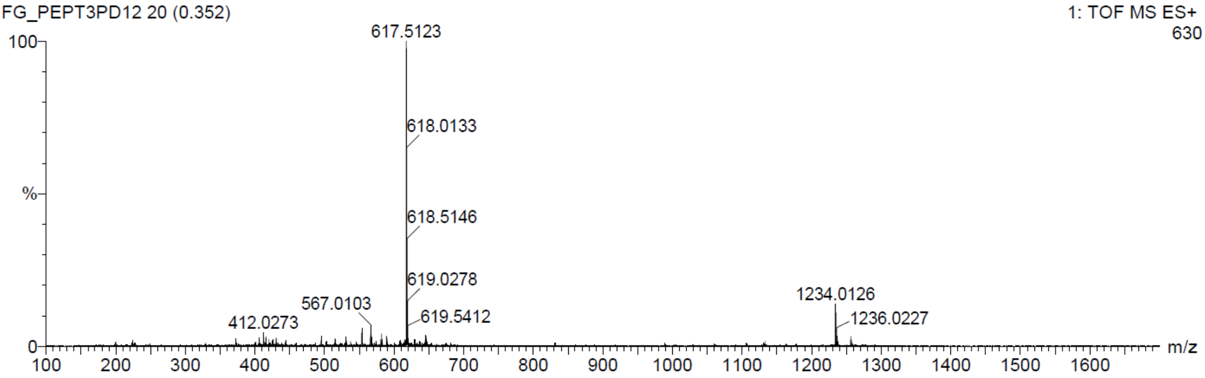

*Figure S23. TIC from TOF MS ES+ analysis of crude peptide **5**.*

Peptide 6

AlaCysAlaThrGlySerThrGlnHisGlnCysGly-NH_2 – Calculated Exact mass: 1161.4655 – Molecular Weight: 1162.2630 – RT: 7.4 min – ESI-MS (*m/z*): 1162.5264 $[M+H]^+$, 581.7865 $[M+2H]^{2+}$

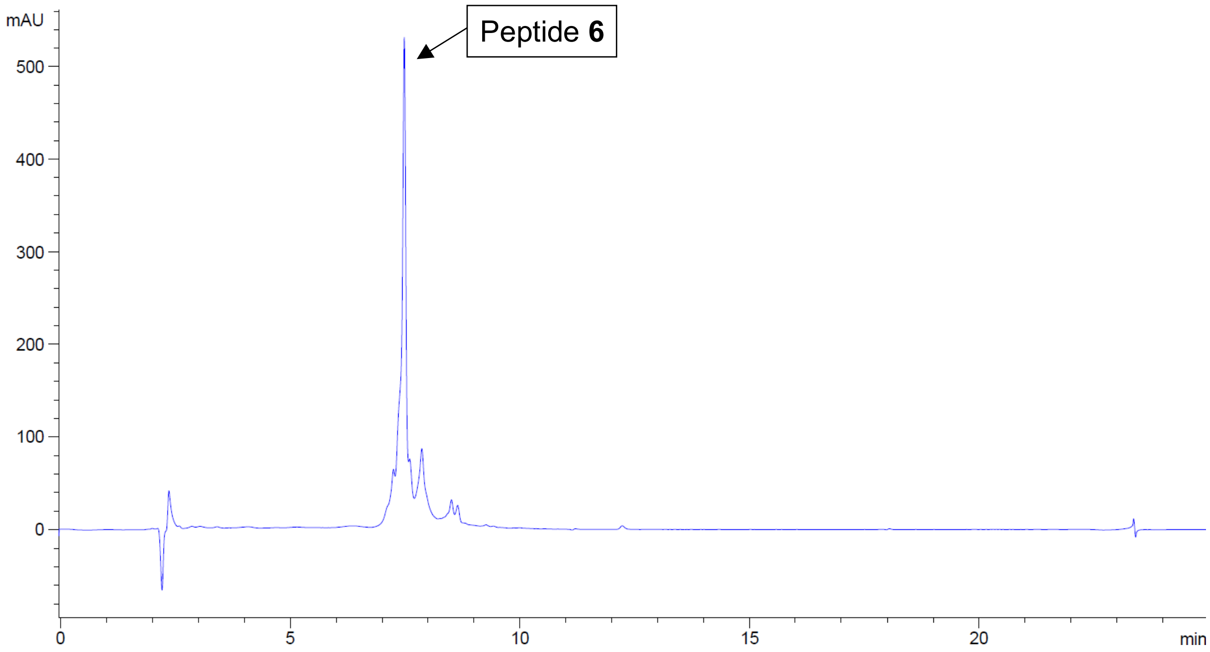

*Figure S24. Analytical HPLC of crude peptide **6**.*

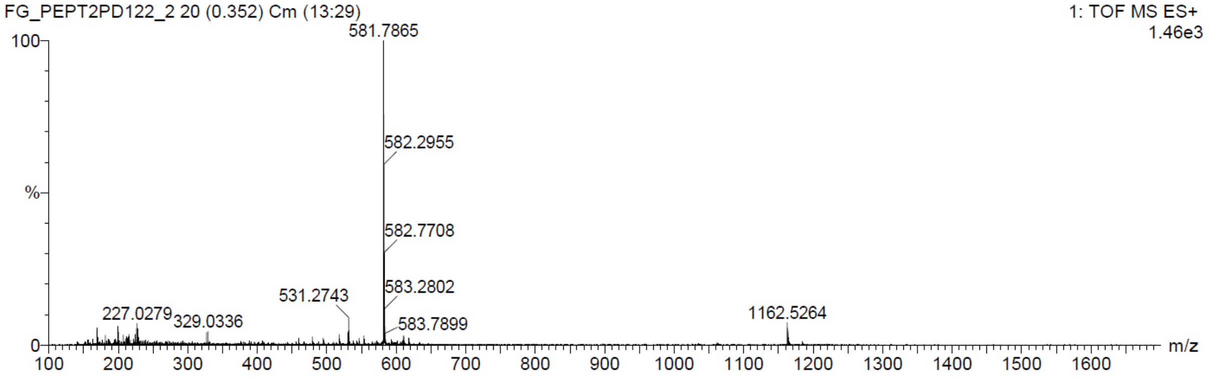

*Figure S25. TIC from TOF MS ES+ analysis of crude peptide **6**.*

Peptide 7

AlaCysThrSerThrGlnHisGlnCysGly-NH_2 – Calculated Exact mass: 1033.4069 – Molecular Weight: 1034.1320 – RT: 7.2 min – ESI-MS (*m/z*): 1034.5149 $[M+H]^+$, 517.7452 $[M+2H]^{2+}$

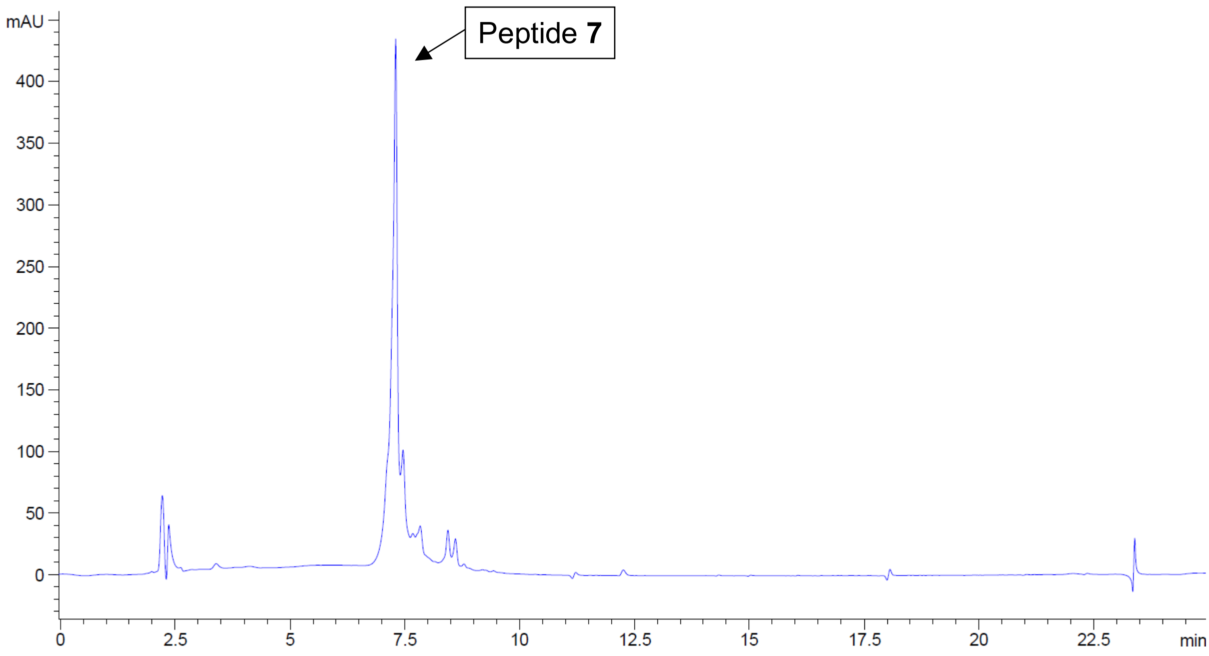

*Figure S26. Analytical HPLC of crude peptide **7**.*

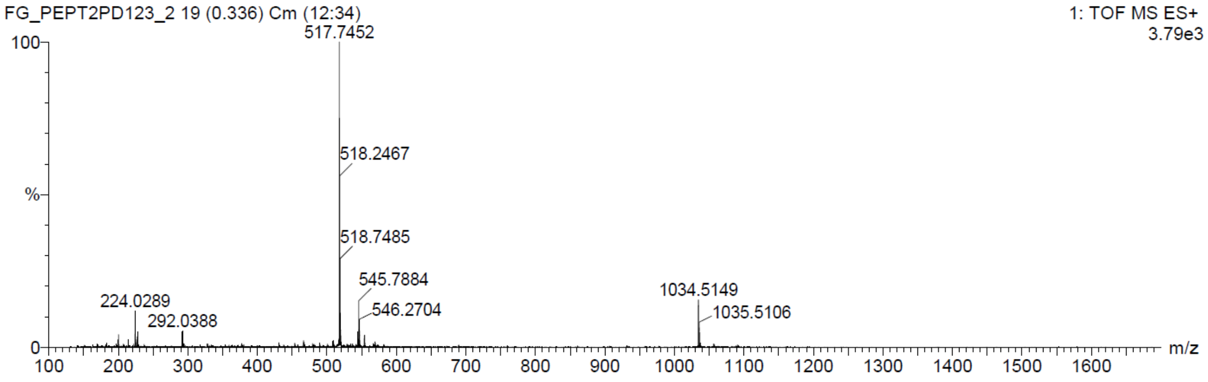

*Figure S27. TIC from TOF MS ES+ analysis of crude peptide **7**.*

Peptide 8

AlaCysSerThrGlnHisGlnCysGly-NH_2 – Calculated Exact mass: 932.3593 – Molecular Weight: 933.0270 – RT: 4.8 min – ESI-MS (*m/z*): 933.6769 $[M+H]^+$, 467.3278 $[M+2H]^{2+}$

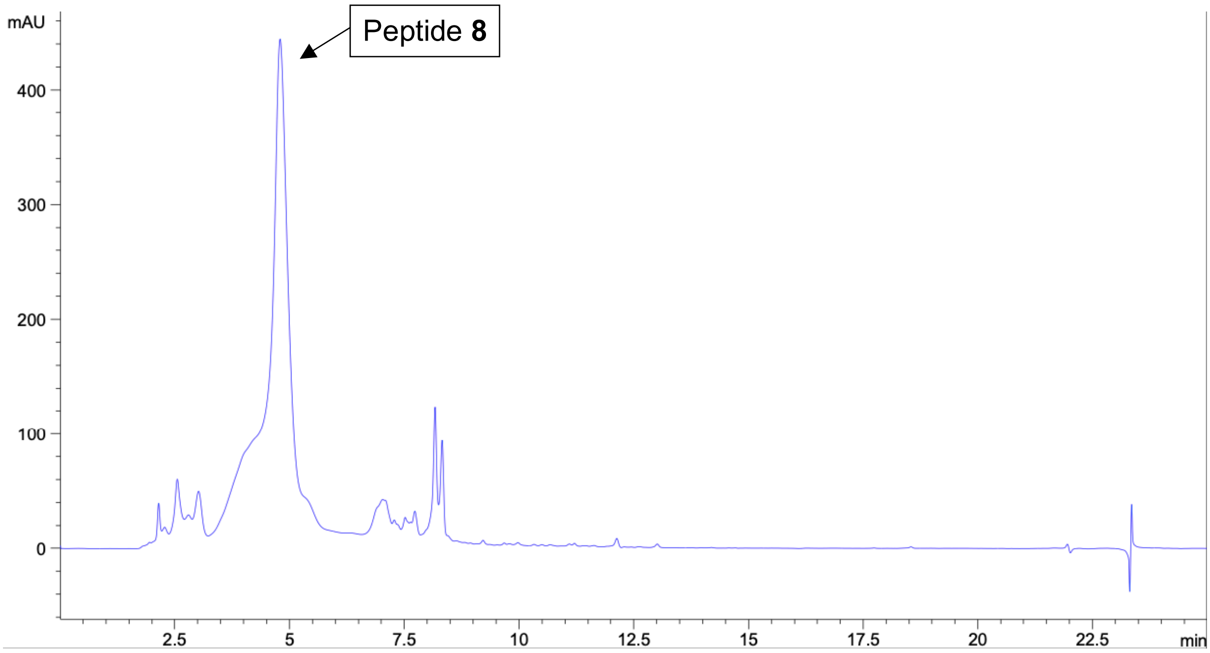

*Figure S28. Analytical HPLC of crude peptide **8**.*

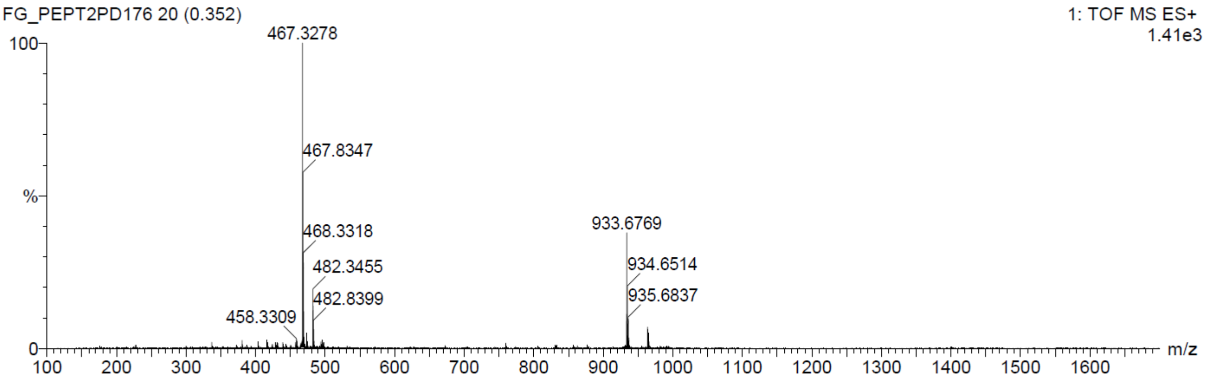

*Figure S29. TIC from TOF MS ES+ analysis of crude peptide **8**.*

Peptide 9

AlaCysThrGlnHisGlnCysGly-NH_2 – Calculated Exact mass: 845.3272 – Molecular Weight: 845.9490 – RT: 4.2 min – ESI-MS (*m/z*): 846.7586 $[M+H]^+$, 423.8545 $[M+2H]^{2+}$

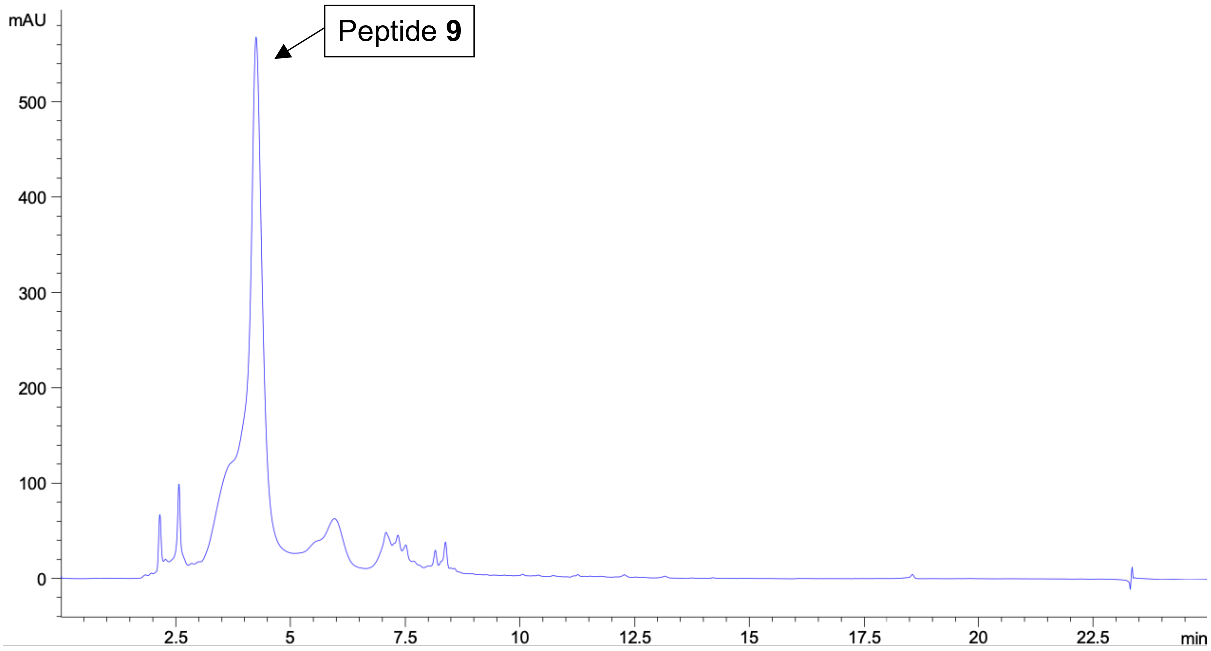

*Figure S30. Analytical HPLC of crude peptide **9**.*

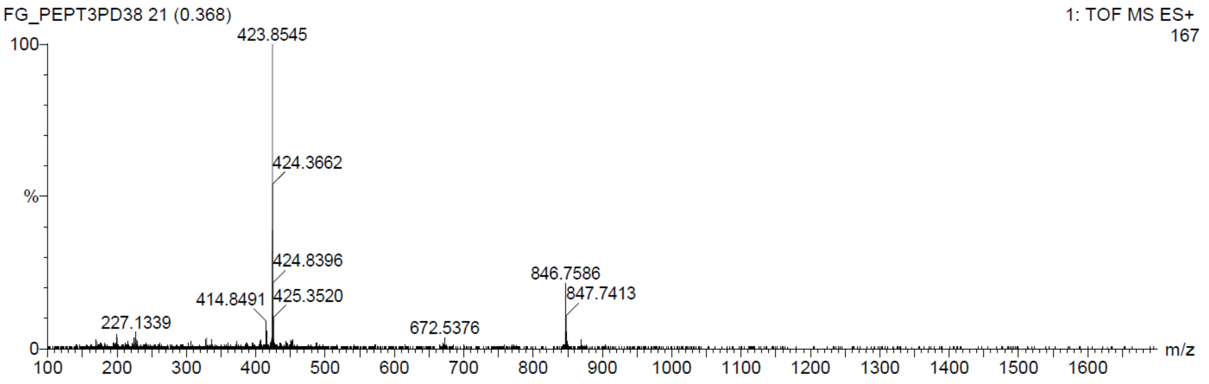

*Figure S31. TIC from TOF MS ES+ analysis of crude peptide **9**.*

Peptide 10

AlaCysGlnHisGlnCysGly-NH_2 – Calculated Exact mass: 744.2796 – Molecular Weight: 744.8440 – RT: 3.1 min – ESI-MS (*m/z*): 745.4860 $[M+H]^+$, 373.2356 $[M+2H]^{2+}$

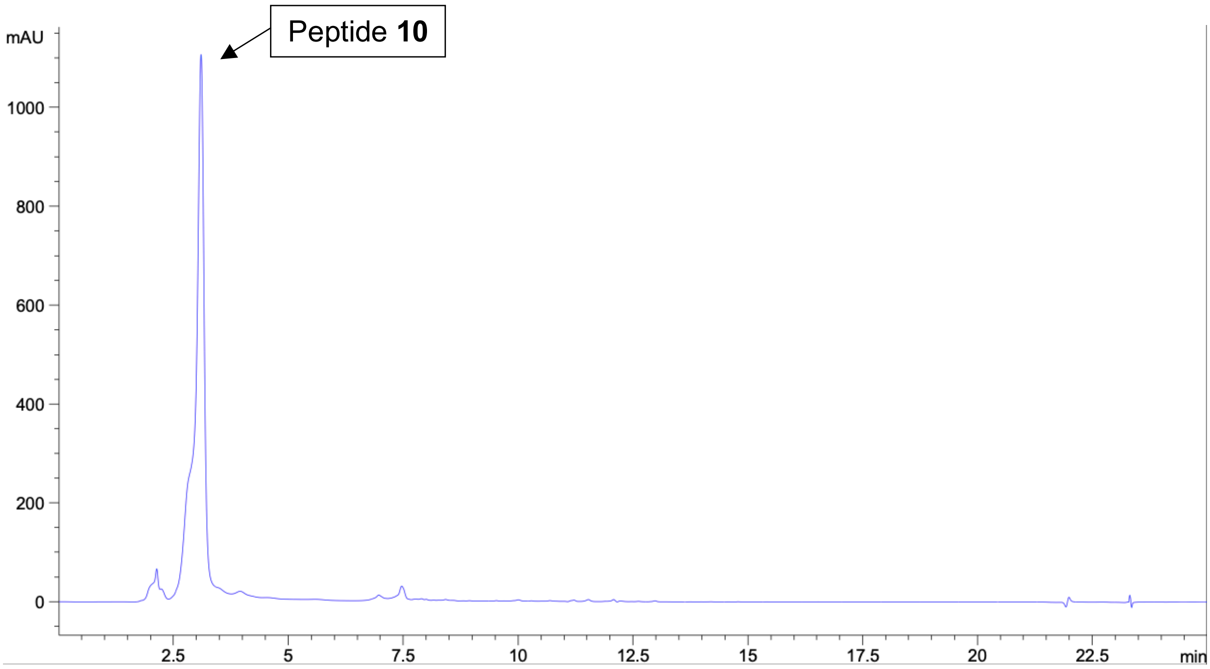

*Figure S32. Analytical HPLC of crude peptide **10**.*

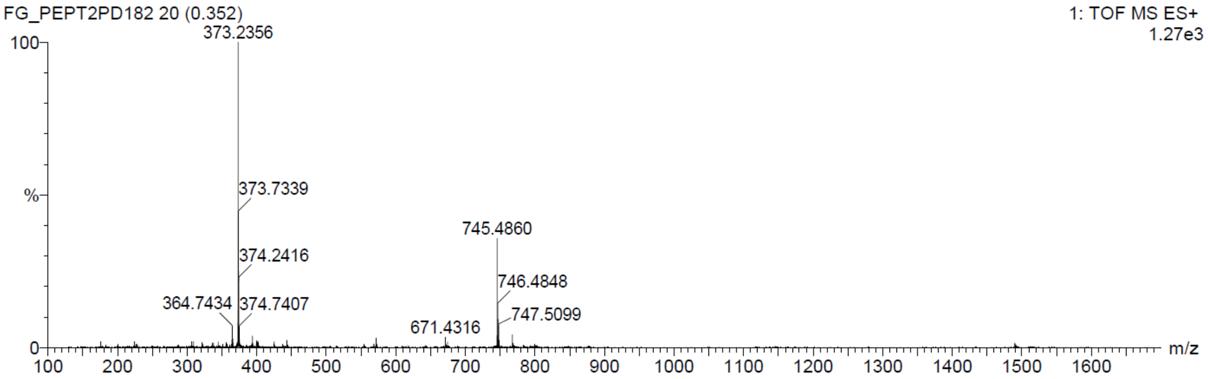

*Figure S33. TIC from TOF MS ES+ analysis of crude peptide **10**.*

Peptide 11

AlaCysHisGlnCysGly-NH_2 – Calculated Exact mass: 616.2210 – Molecular Weight: 616.7130 – RT: 2.9 min – ESI-MS (*m/z*): 1233.9468 $[2M+H]^+$, 617.5123 $[M+H]^+$, 309.2538 $[M+2H]^{2+}$

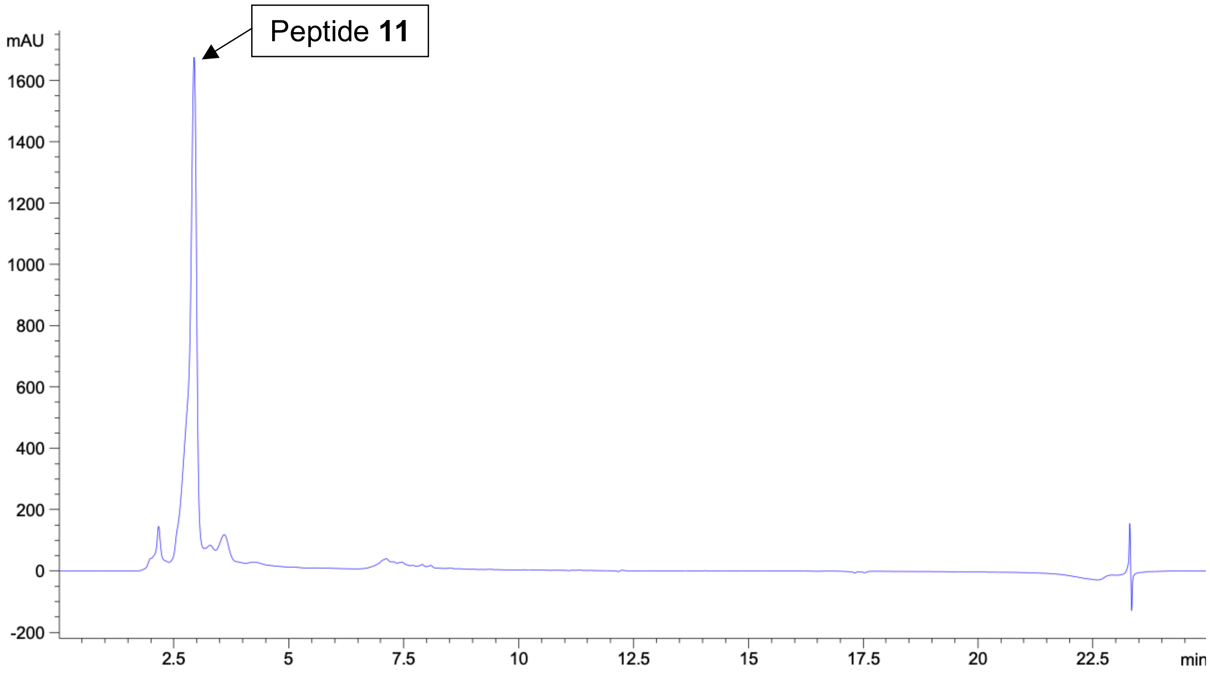

*Figure S34. Analytical HPLC of crude peptide **11**.*

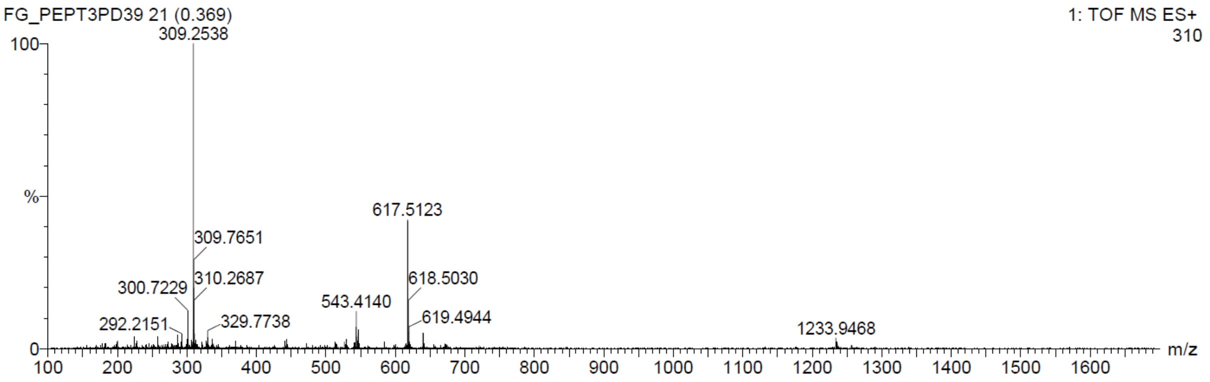

*Figure S35. TIC from TOF MS ES+ analysis of crude peptide **11**.*

Peptide 12

AlaCysAlaCysGly-NH_2 – Calculated Exact mass: 422.1406 – Molecular Weight: 422.5190 – RT: 3.3 min – ESI-MS (*m/z*): 845.3962 $[2M+H]^+$, 423.1778 $[M+H]^+$

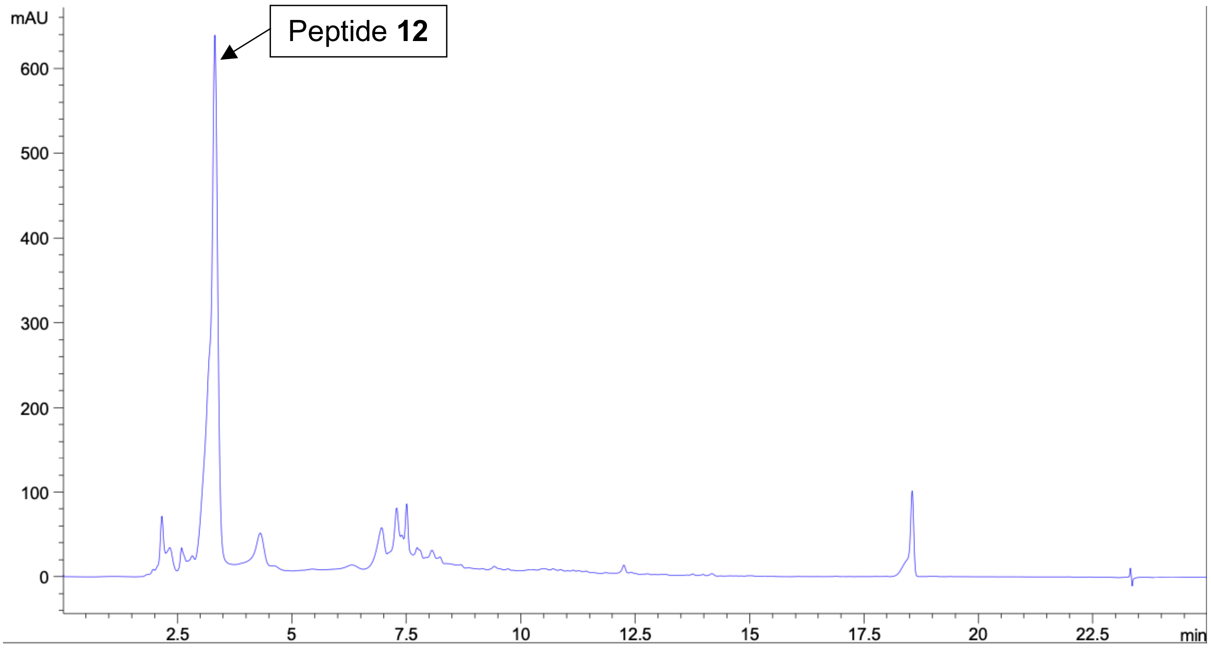

*Figure S36. Analytical HPLC of crude peptide **12**.*

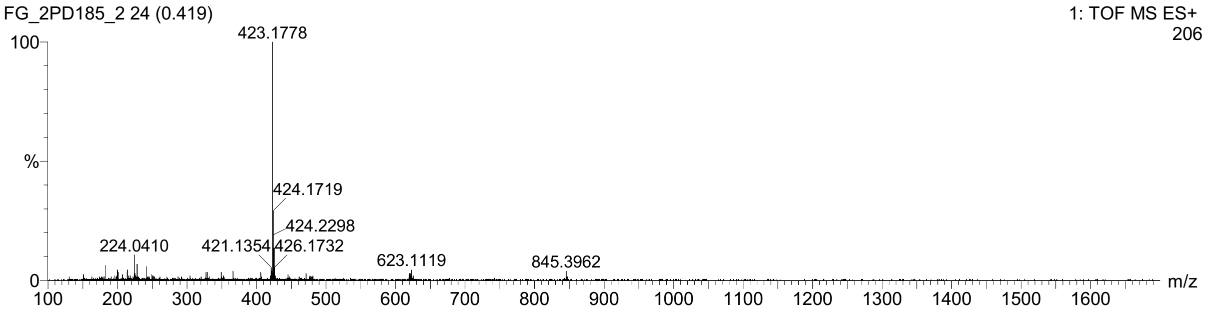

*Figure S37. TIC from TOF MS ES+ analysis of crude peptide **12**.*

Peptide 13

AlaCysHisCysGly-NH_2 – Calculated Exact mass: 488.1624 – Molecular Weight: 488.5820 – RT: 2.7 min – ESI-MS (*m/z*): 489.3728 $[2M+H]^+$

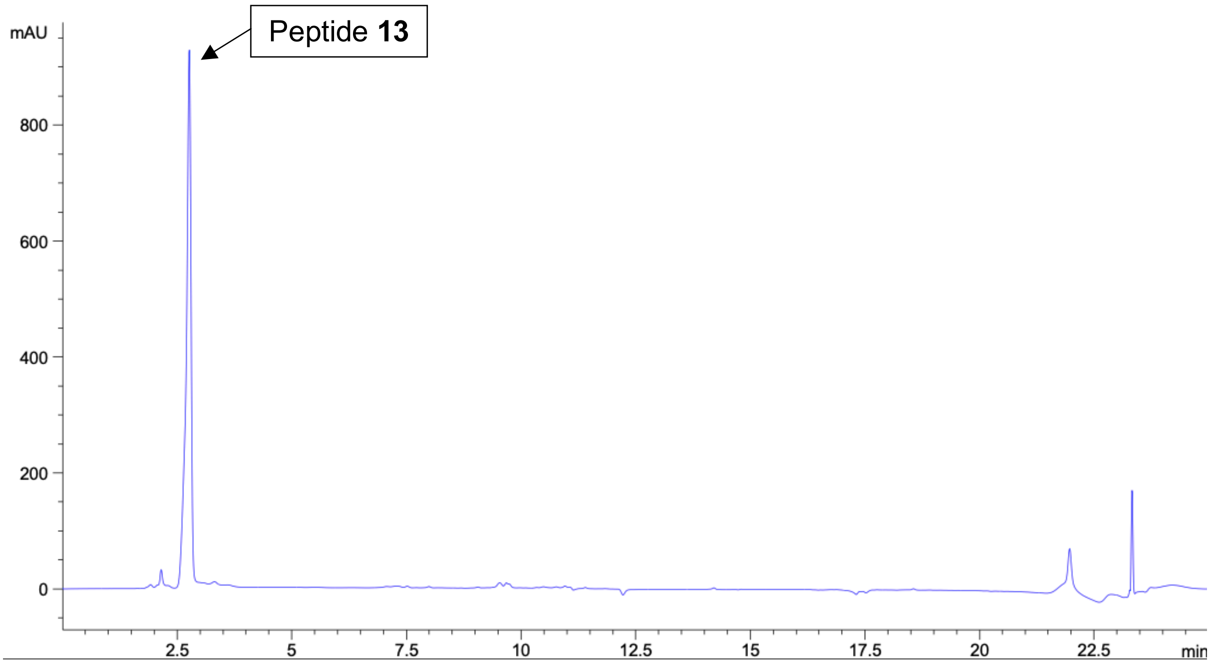

*Figure S38. Analytical HPLC of crude peptide **13**.*

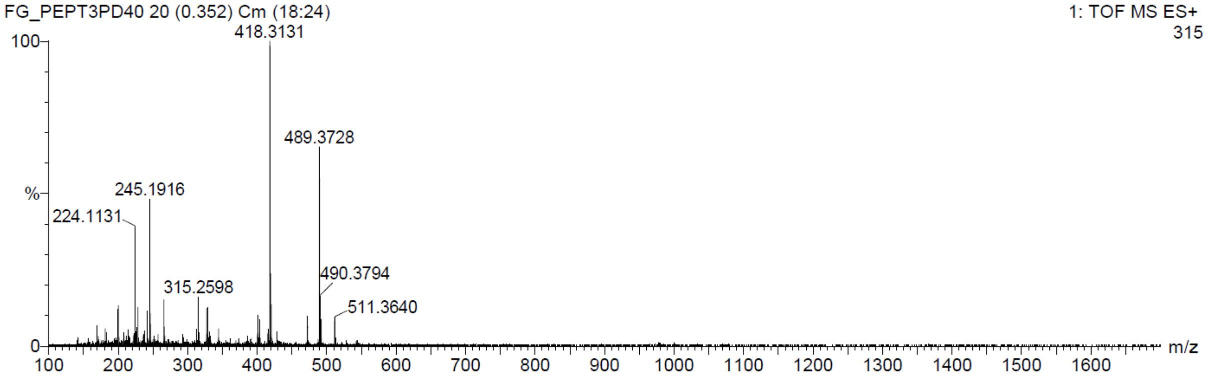

*Figure S39. TIC from TOF MS ES+ analysis of crude peptide **13**.*

Peptide 14

AlaCysCysGly-NH_2 – Calculated Exact mass: 351.1035 – Molecular Weight: 351.4400 – RT: 2.8 min – ESI-MS (*m/z*): 1054.7456 $[3M+H]^+$, 703.5029 $[2M+H]^+$, 352.2367 $[M+H]^+$

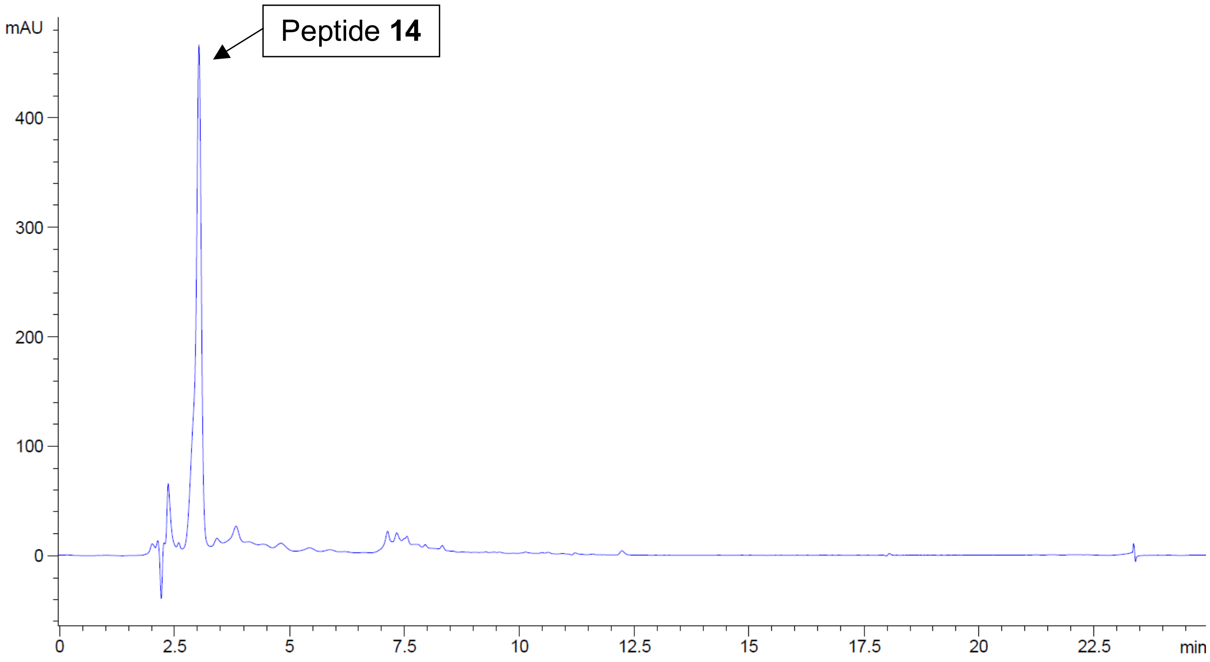

*Figure S40. Analytical HPLC of crude peptide **14**.*

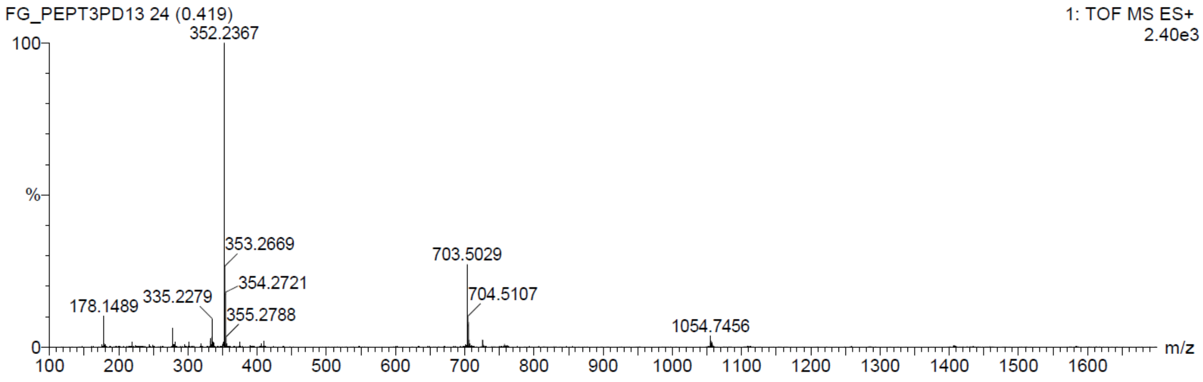

*Figure S41. TIC from TOF MS ES+ analysis of crude peptide **14**.*

Peptide 15

AlaCysThrHisGlyGlnThrGlnSerCysGly-NH_2 – Calculated Exact mass: 1090.4284 – Molecular Weight: 1091.1840 – RT: 7.2 min – ESI-MS (*m/z*): 1091.6542 $[M+H]^+$, 546.3252 $[M+2H]^{2+}$

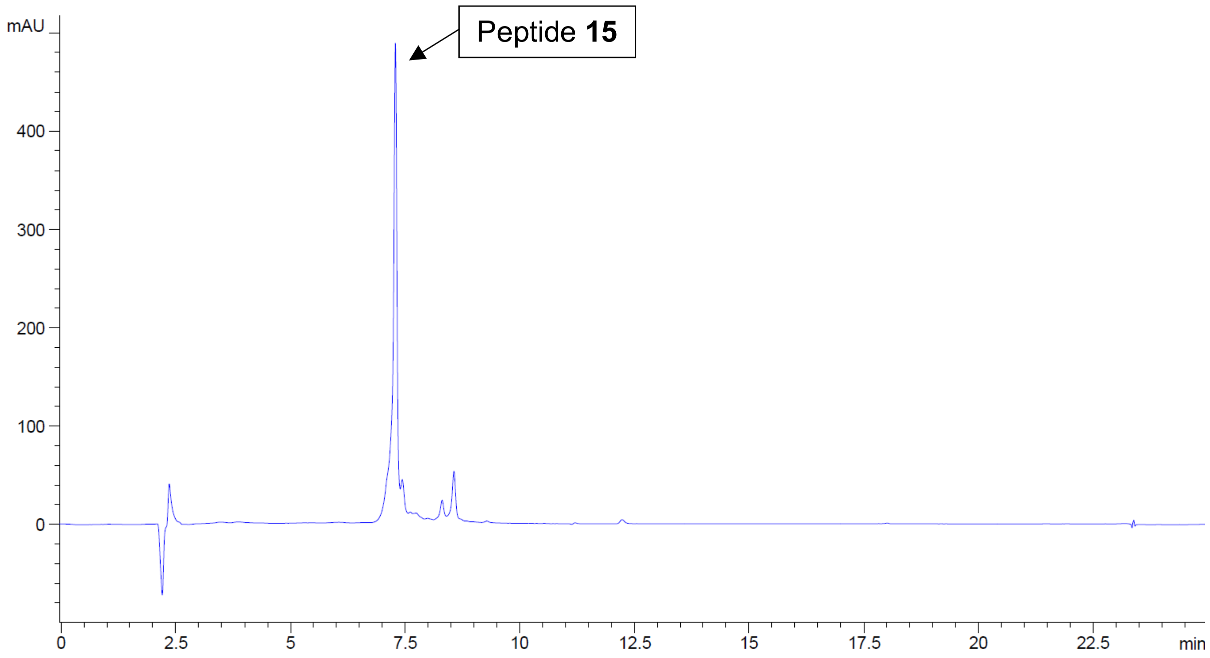

*Figure S42. Analytical HPLC of crude peptide **15**.*

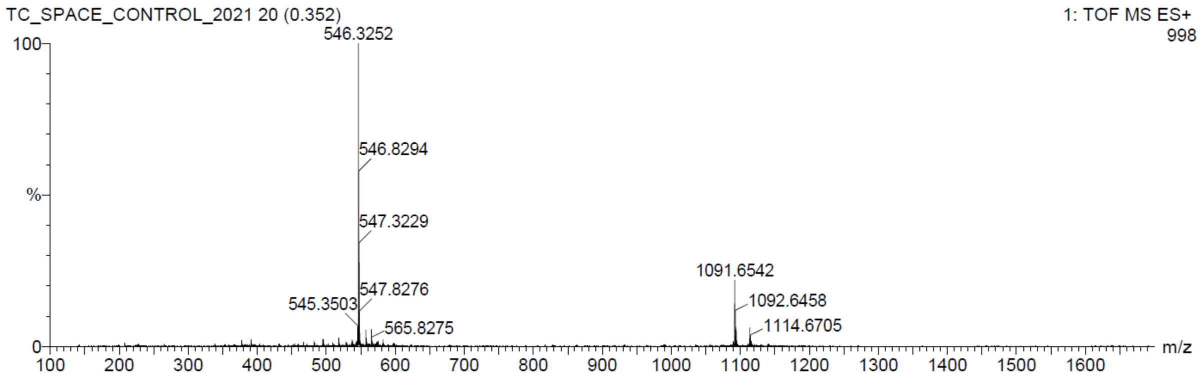

*Figure S43. TIC from TOF MS ES+ analysis of crude peptide **15**.*

Peptide 16

AsnCysValValGlyTyrIleGlyGluArgCysGln-NH_2 – Calculated Exact mass: 1338.6173 – Molecular Weight: 1339.5540 – RT: 10.2 min – ESI-MS (*m/z*): 1340.0433 $[M+H]^+$, 670.4722 $[M+2H]^{2+}$, 447.3465 $[M+3H]^{3+}$

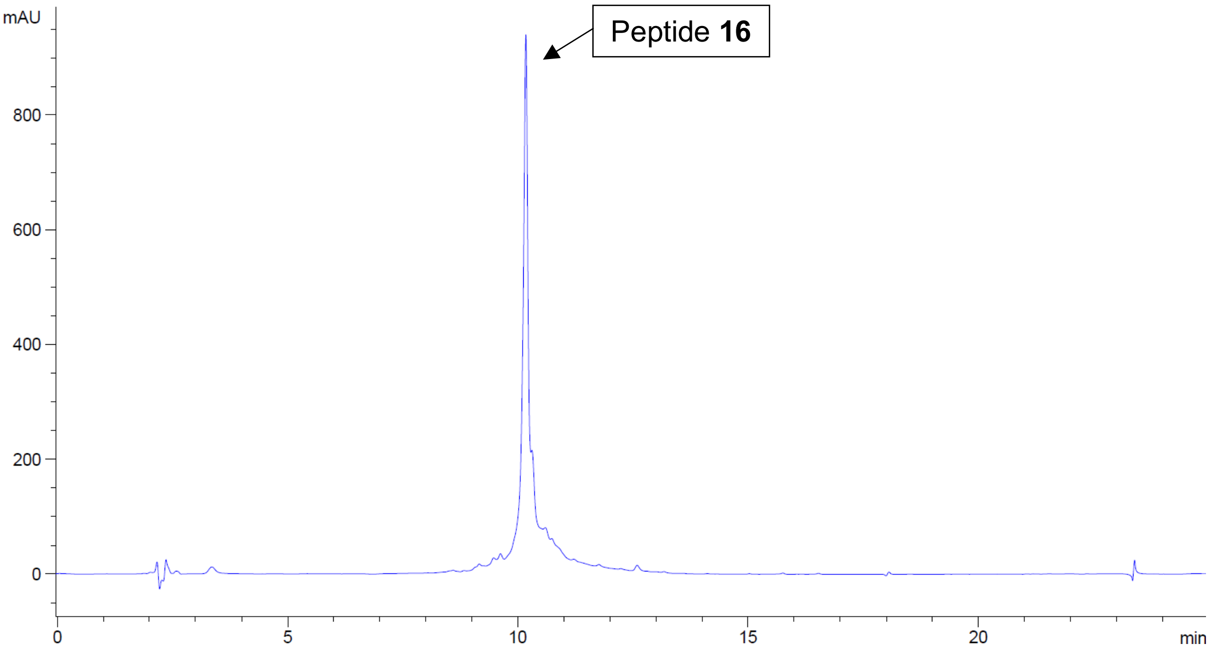

*Figure S44. Analytical HPLC of crude peptide **16**.*

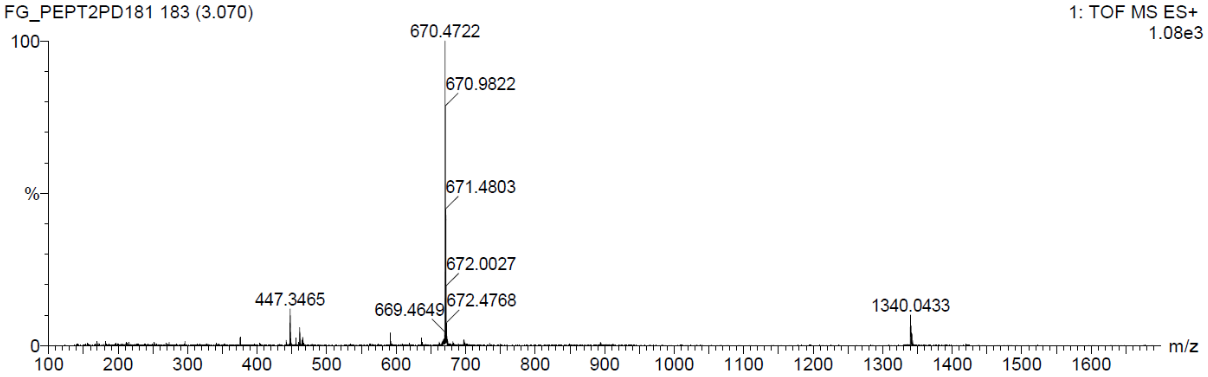

*Figure S45. TIC from TOF MS ES+ analysis of crude peptide **16**.*

Peptide 17

CysLysAlaProGluThrAlaLeuCys-NH_2 – Calculated Exact mass: 933.4412 – Molecular Weight: 934.1390 – RT: 9.3 min - ESI-MS (*m/z*): 934.4890 $[M+H]^+$, 467.7361 $[M+2H]^{2+}$

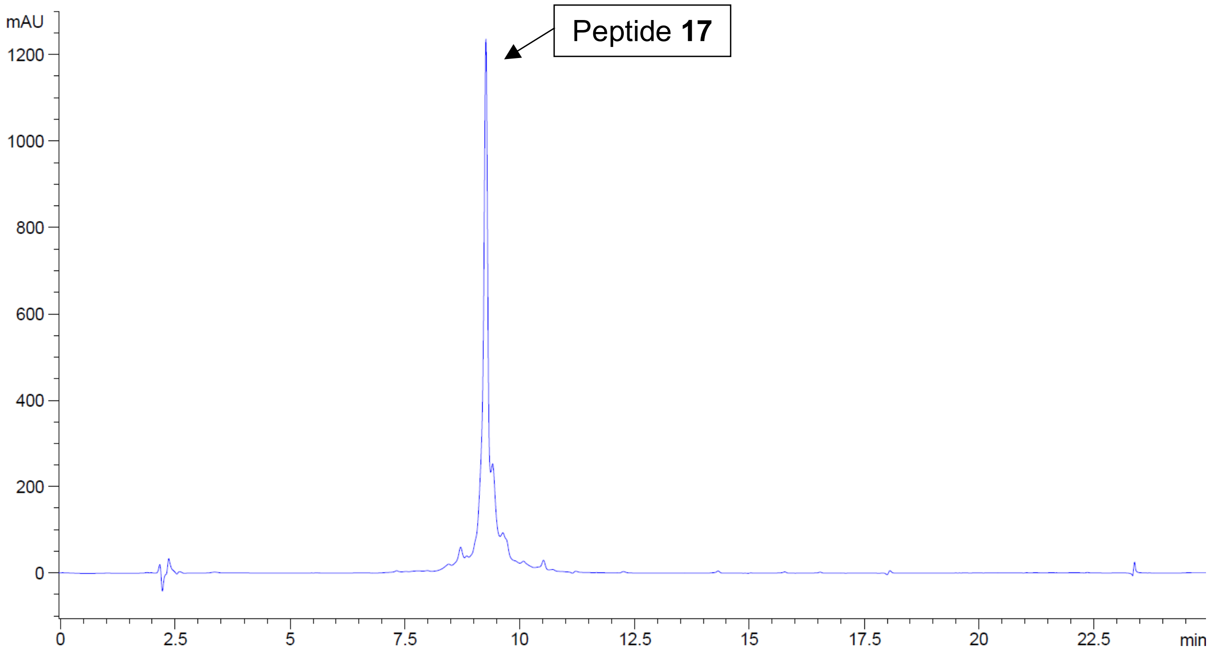

*Figure S46. Analytical HPLC of crude peptide **17**.*

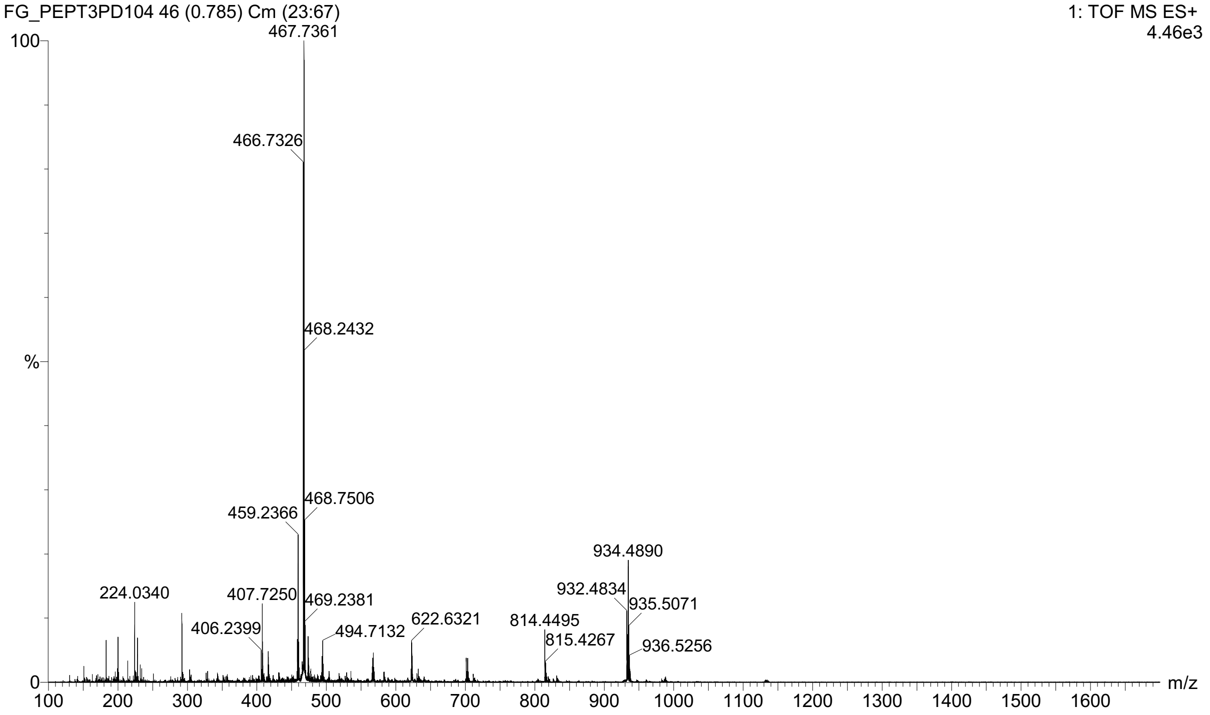

*Figure S47. TIC from TOF MS ES+ analysis of crude peptide **17**.*

Peptide 18

AlaGlyTyrLeuLeuGlyLysIleAsnLeuLysAlaCysAlaAlaLeuAlaLysLysCysLeu-NH_2 – Calculated Exact mass: 2160.2639 – Molecular Weight: 2161.7480 – RT: 12.0 min – ESI-MS (*m/z*): 1081.6011 $[M+2H]^{2+}$, 721.4022 $[M+3H]^{3+}$, 541.3055 $[M+4H]^{4+}$

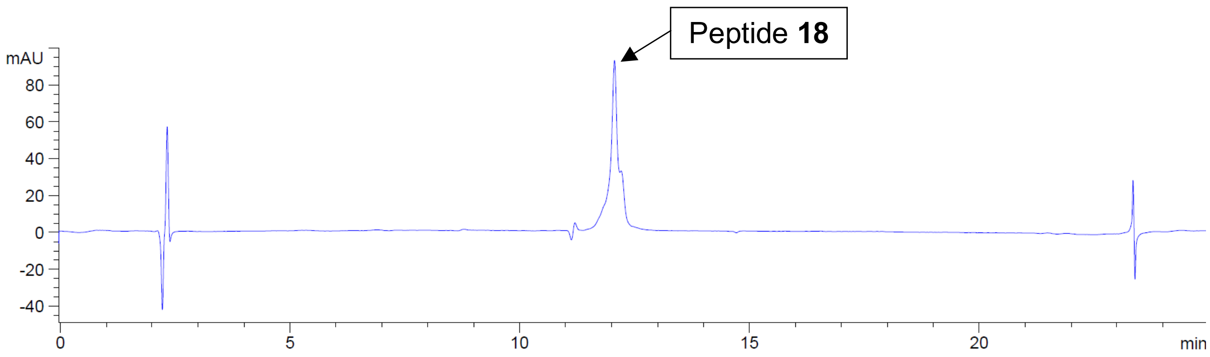

*Figure S48. Analytical HPLC of purified peptide **18**.*

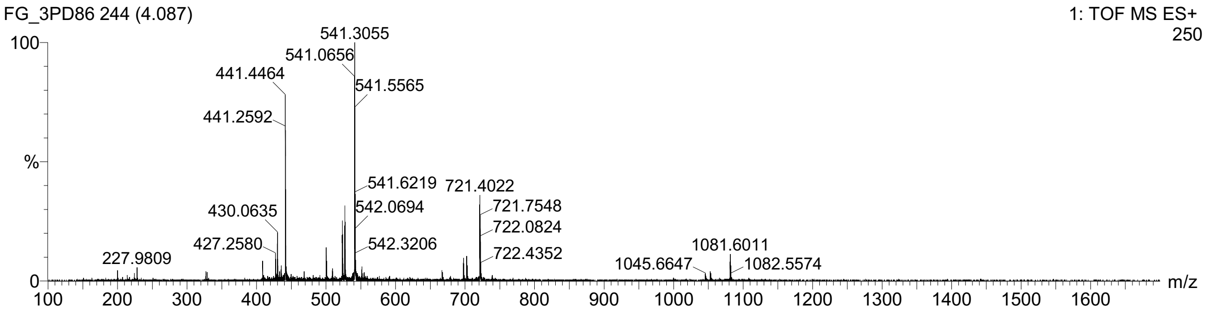

*Figure S49. TIC from TOF MS ES+ analysis of crude peptide **18**.*

Peptide 43

AlaCysThrGlnHisGlnCysThrGlnHisGlnCysGly-NH_2 – Calculated Exact mass: 1442.5602 – Molecular Weight: 1443.5970 – RT: 7.4 min – ESI-MS (*m/z*): 1443.7104 $[M+H]^+$, 722.2967 $[M+2H]^{2+}$, 481.8657 $[M+3H]^{3+}$

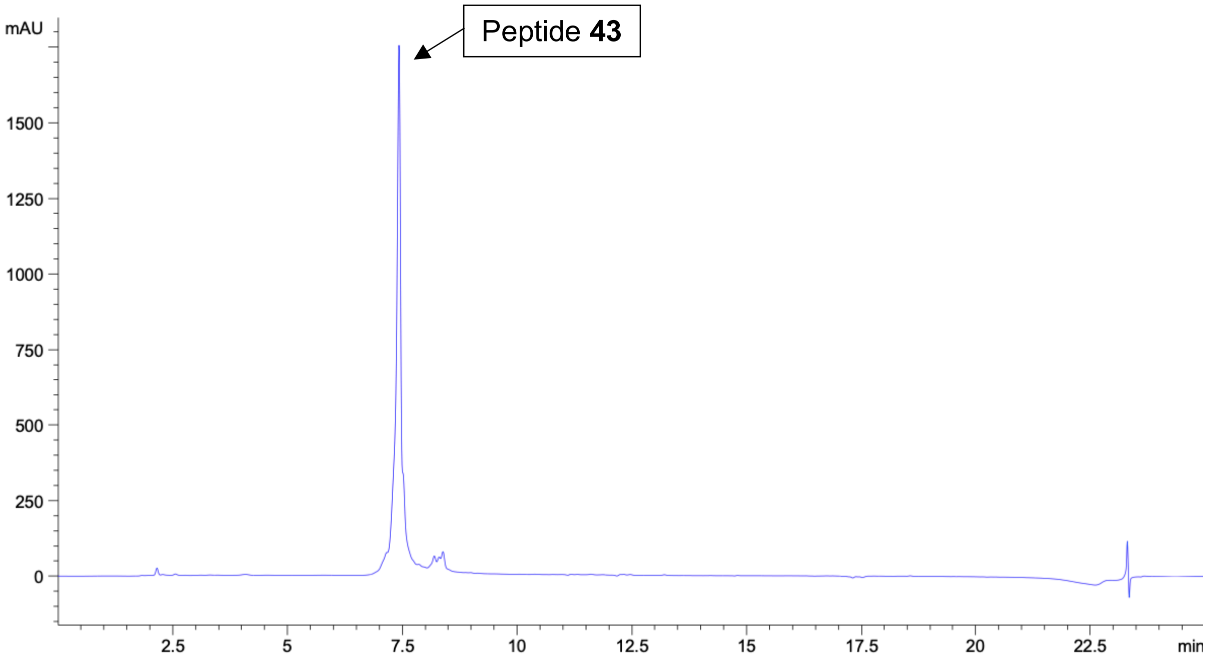

*Figure S50. Analytical HPLC of crude peptide **43**.*

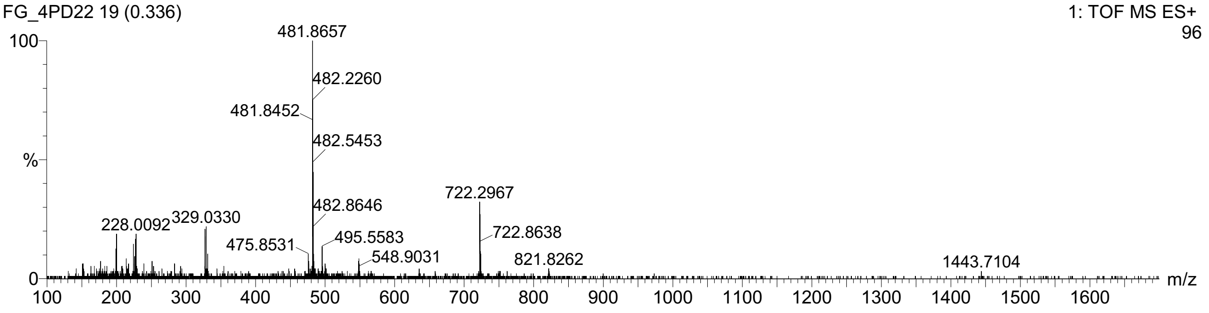

*Figure S51. TIC from TOF MS ES+ analysis of crude peptide **43**.*

Peptide 44

AlaCysThrGlnHisGlnCysThrGlnHisGlnCysThrGlnHisGlnCysGly-NH_2 – Calculated Exact mass: 2039.7931 – Molecular Weight: 2041.2450 – RT: 7.6 min – ESI-MS (*m/z*): 1021.3347 $[M+2H]^{2+}$, 680.9044 $[M+3H]^{3+}$, 511.1588 $[M+4H]^{4+}$

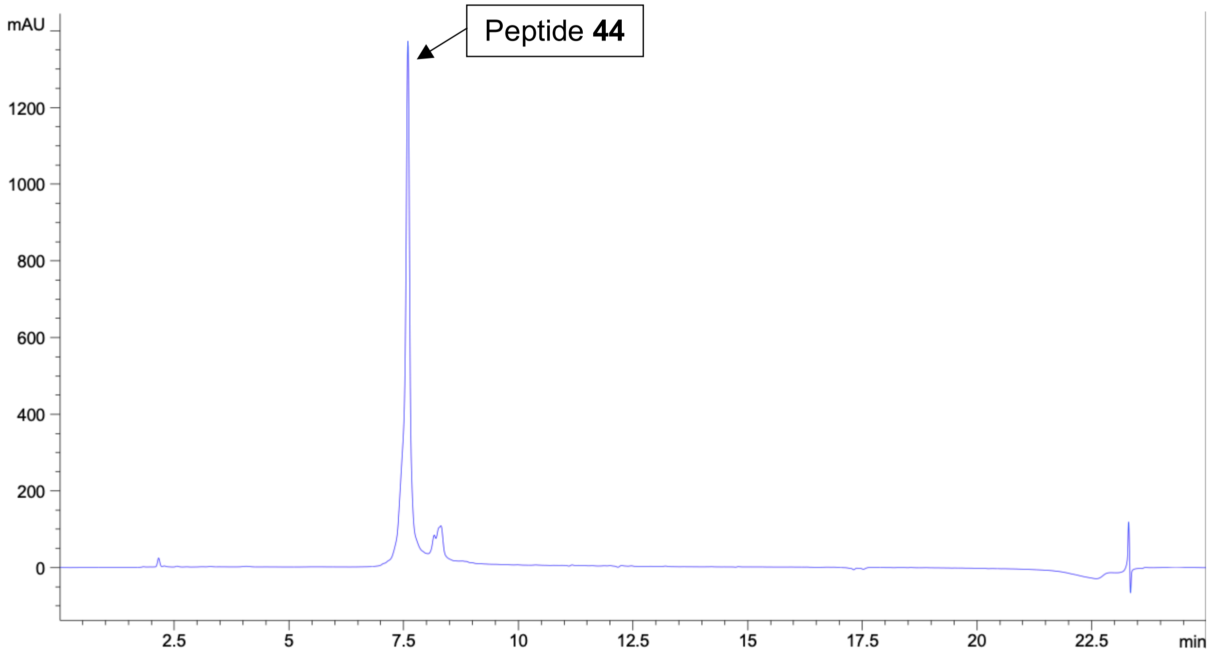

*Figure S52. Analytical HPLC of crude peptide **44**.*

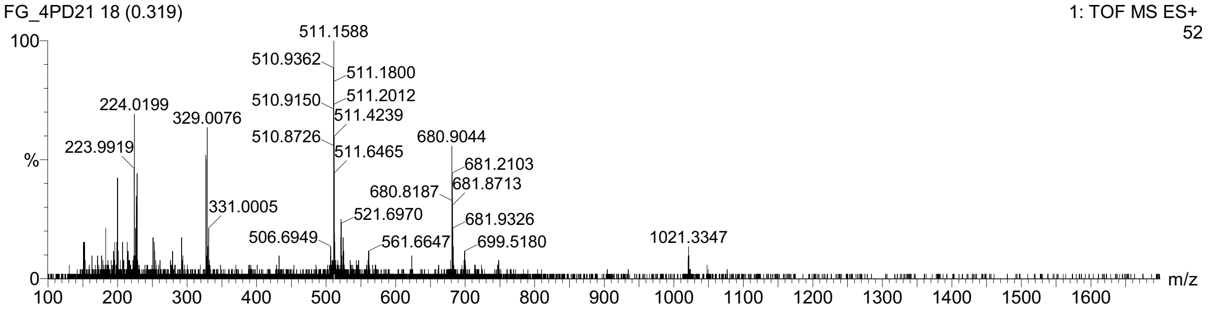

*Figure S53. TIC from TOF MS ES+ analysis of crude peptide **44**.*

Peptide 45

AlaCysSerThrGlnHisGlnCysSerThrGlnHisGlnCysSerThrGlnHisGlnCysGly-NH_2 – Calculated Exact mass: 2300.8892 – Molecular Weight: 2302.4790 – RT: 7.6 min - ESI-MS (*m/z*): 1153.1725 $[M+2H]^{2+}$, 768.7586 $[M+3H]^{3+}$, 576.8235 $[M+4H]^{4+}$

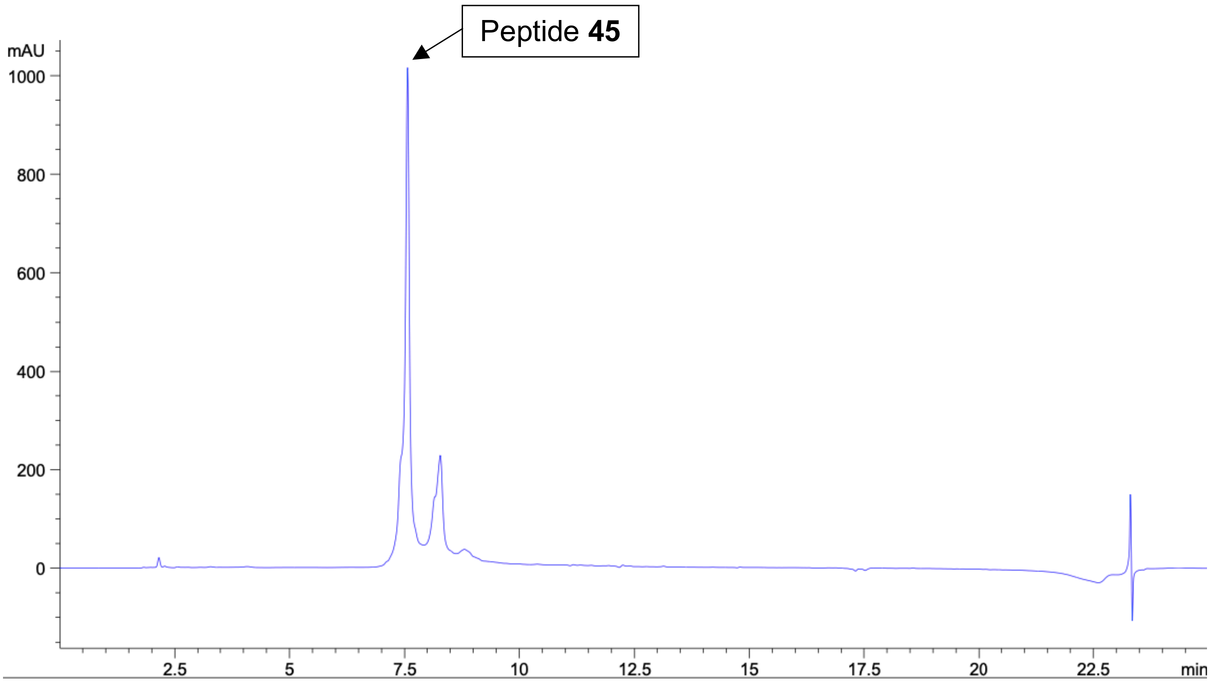

*Figure S54. Analytical HPLC of crude peptide **45**.*

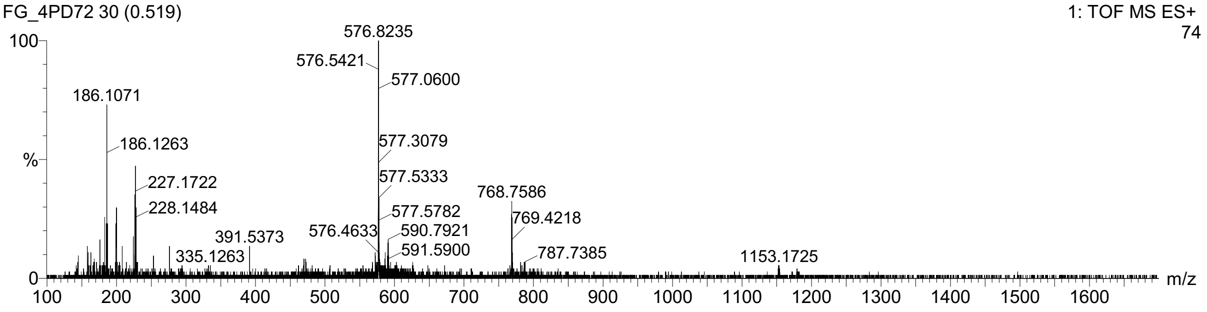

*Figure S55. TIC from TOF MS ES+ analysis of crude peptide **45**.*

Peptide S4

CysAlaAlaAlaLys-OH – Calculated Exact mass: 462.2261 – Molecular Weight: 462.5660 – RT: 2.3 min – ESI-MS (*m/z*): 463.2142 $[M+H]^+$

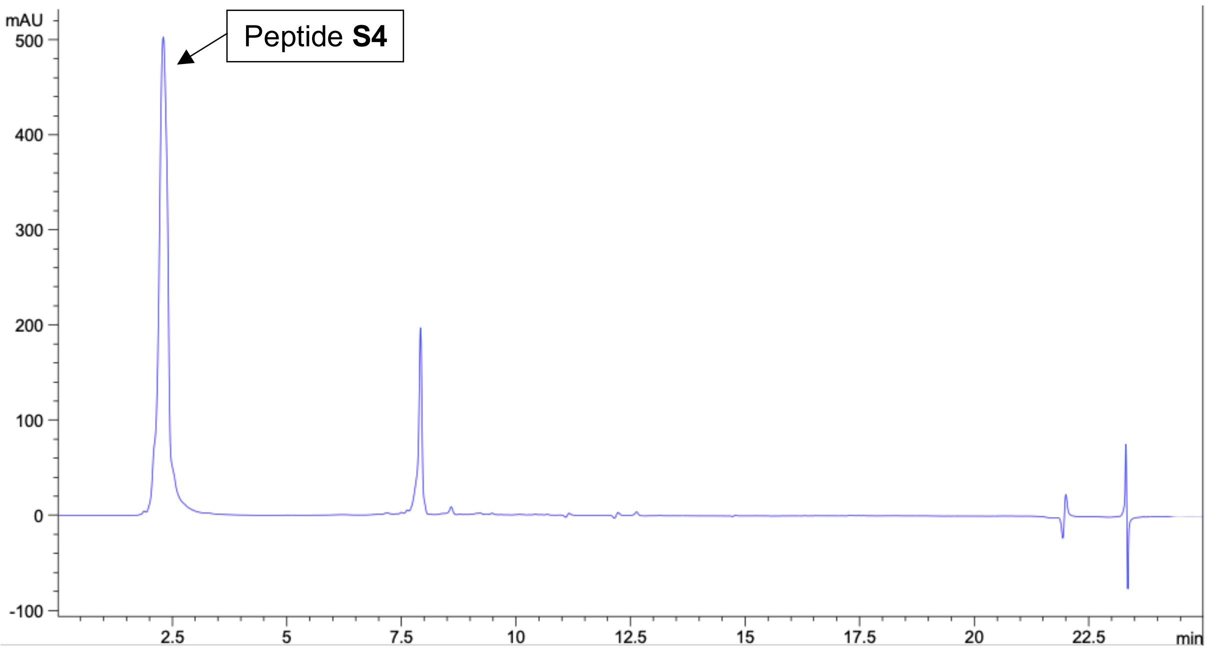

*Figure S56. Analytical HPLC of crude peptide **S4**.*

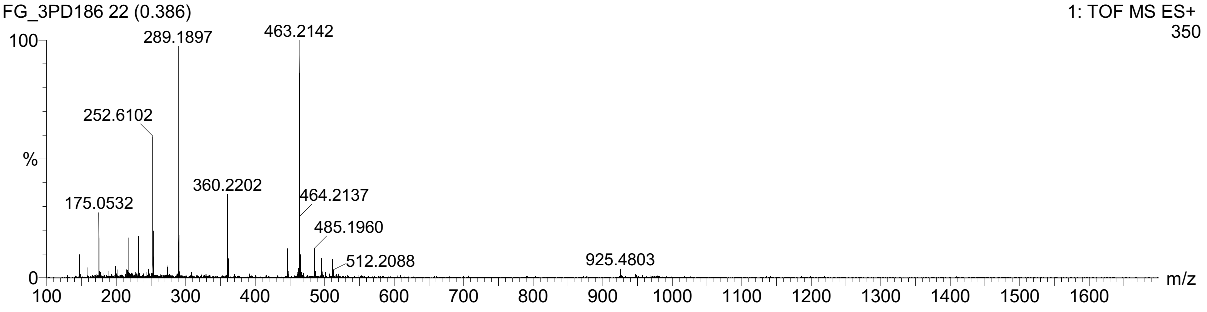

*Figure S57. TIC from TOF MS ES+ analysis of crude peptide **S4**.*

Porphyrin-Peptides Conjugates

Conjugate 19

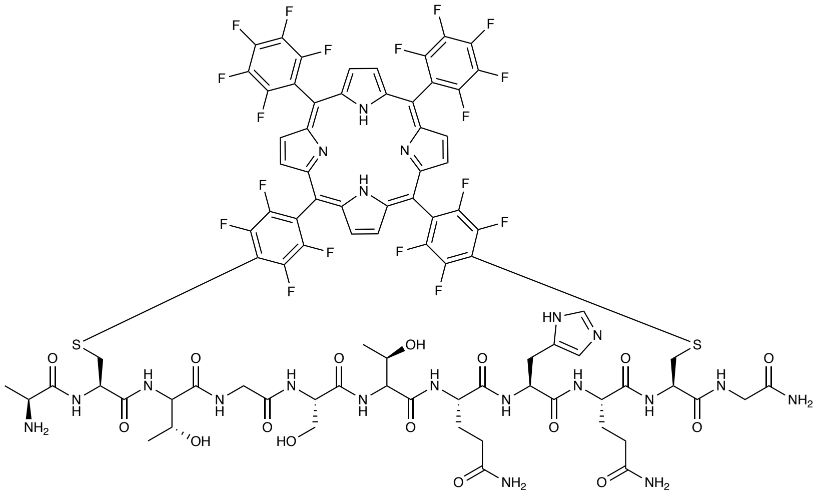

Calculated Exact Mass: 2024.4746 – Molecular Weight: 2025.7313 – RT: 15.9 min – ESI-MS (*m/z*): 1013.8258 $[M+2H]^{2+}$, 689.8533 $[M+H+2Na]^{3+}$, 676.1896 $[M+3H]^{3+}$ – Isolated yield: 18%

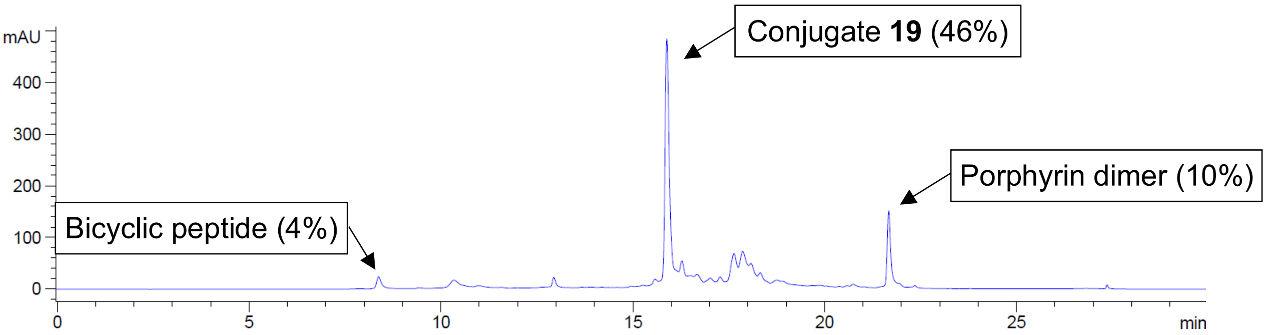

*Figure S58. Analytical HPLC of crude conjugate **19**.*

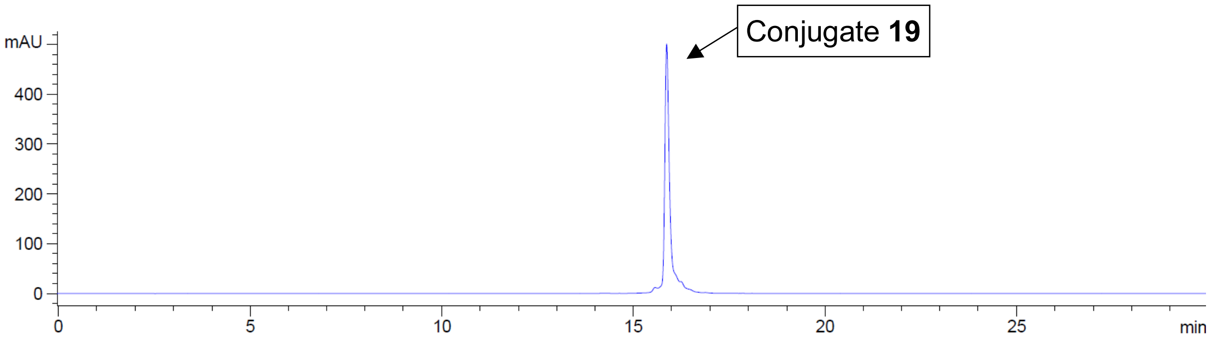

*Figure S59. Analytical HPLC of purified conjugate **19**.*

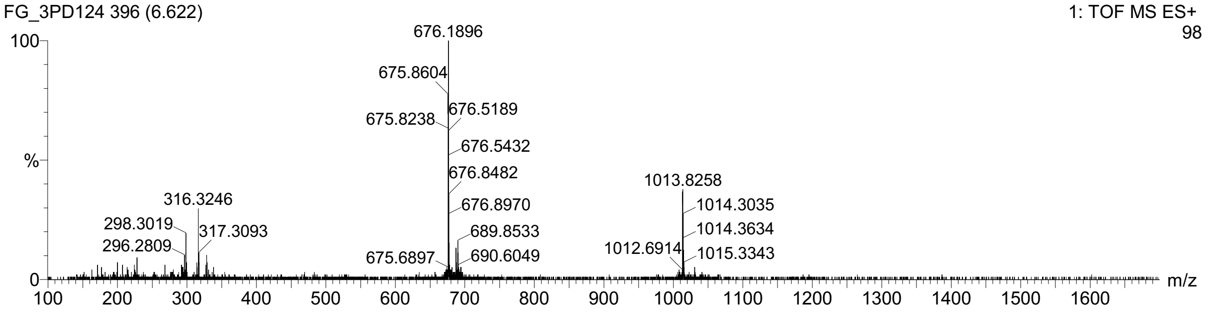

Figure S60. TIC from TOF MS ES+ analysis of crude conjugate ***19***.

Conjugate 20

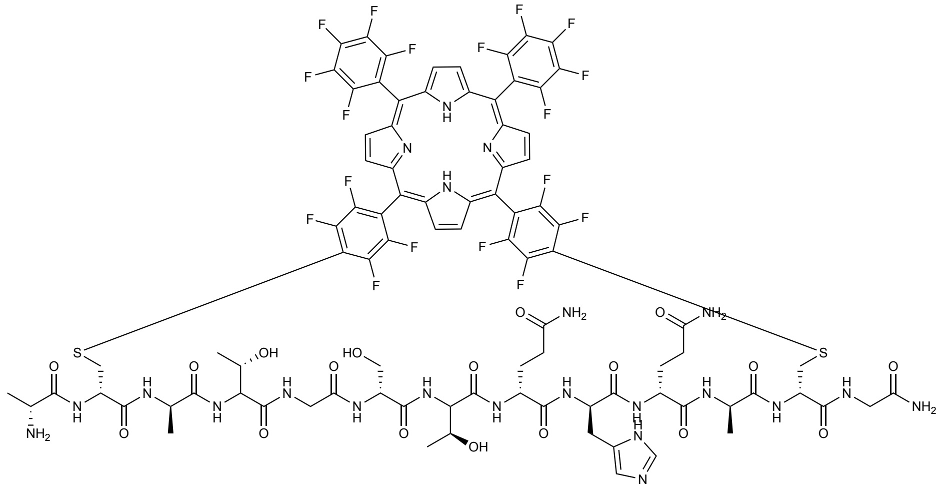

Calculated Exact Mass: 2166.5488 – Molecular Weight: 2167.8893 – RT: 15.9 min – ESI-MS (*m/z*): 1085.0803 $[M+2H]^{2+}$, 723.3773 $[M+3H]^{3+}$

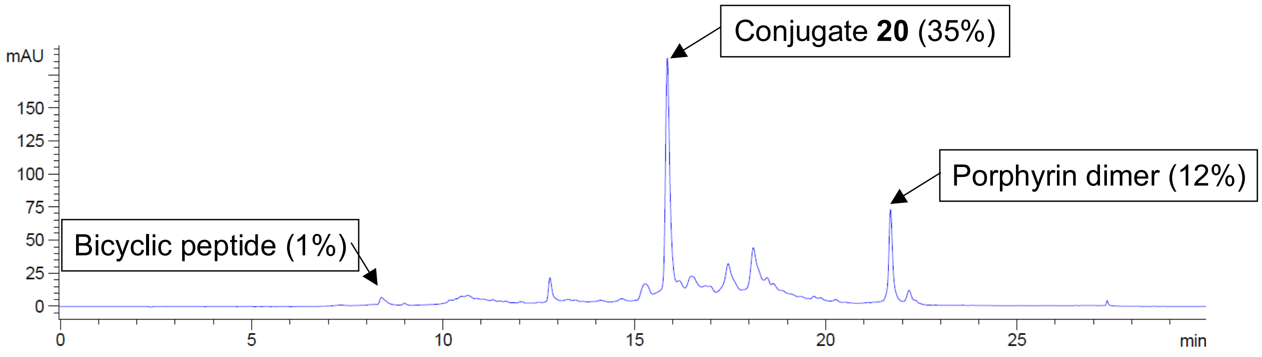

*Figure S61. Analytical HPLC of crude conjugate **20**.*

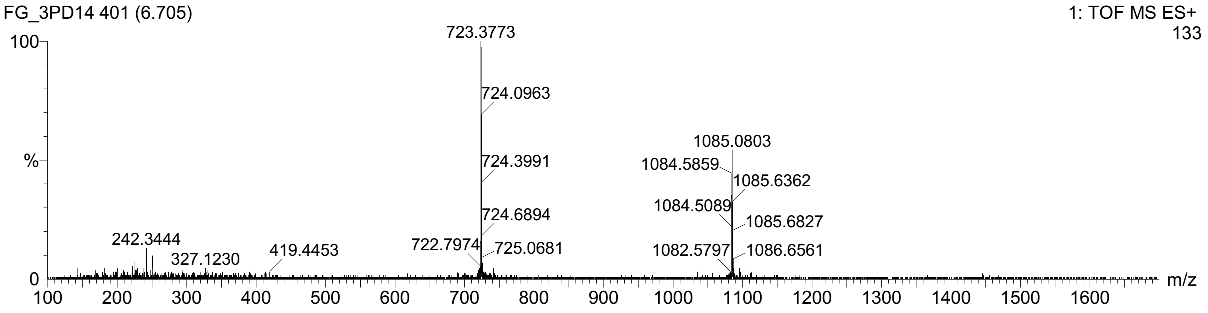

*Figure S62. TIC from TOF MS ES+ analysis of crude conjugate **20**.*

Conjugate 21

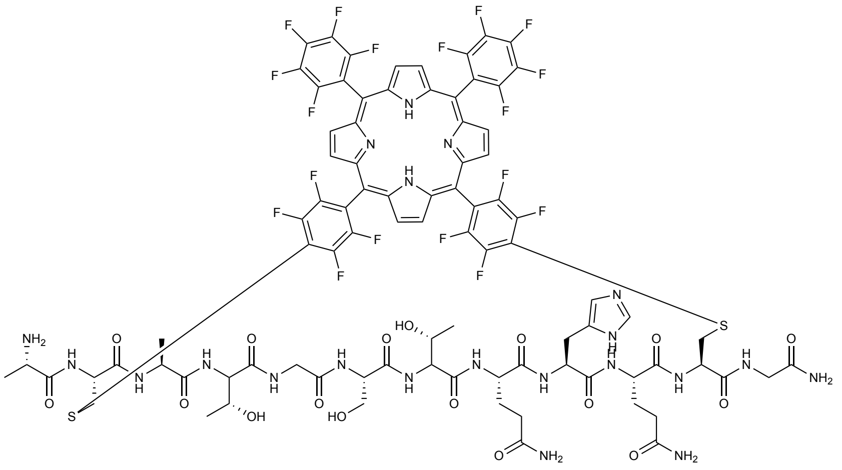

Calculated Exact Mass: 2095.5117 – Molecular Weight: 2096.8103 – RT: 15.5 min – ESI-MS (*m/z*): 1049.3163 $[M+2H]^{2+}$, 714.2386 $[M+H+2Na]^{3+}$, 699.8887 $[M+3H]^{3+}$

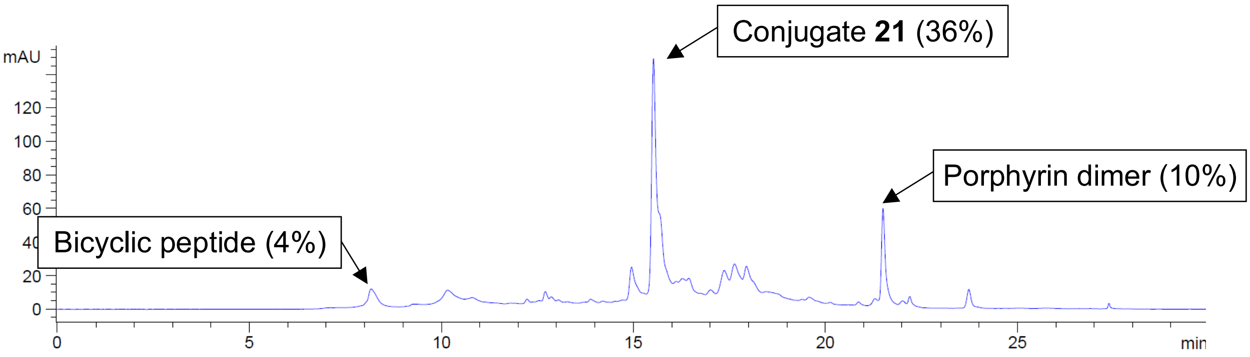

*Figure S63. Analytical HPLC of crude conjugate **21**.*

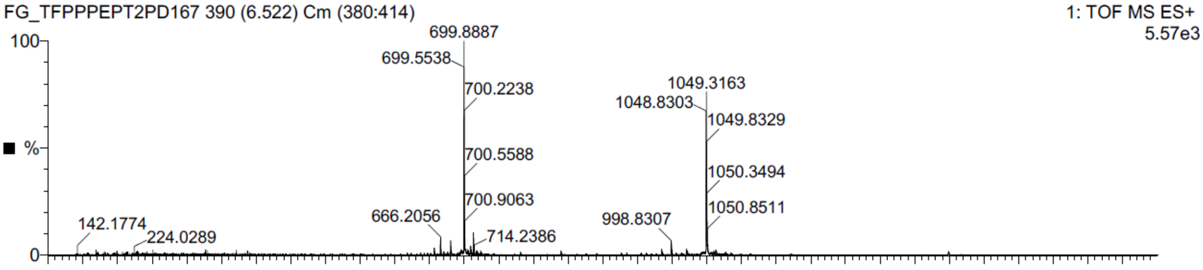

*Figure S64. TIC from TOF MS ES+ analysis of crude conjugate **21**.*

Conjugate 22

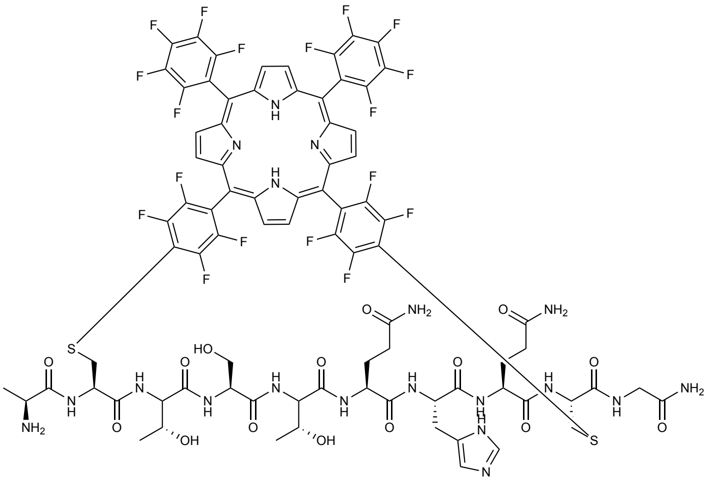

Calculated Exact Mass: 1967.4531 – Molecular Weight: 1968.6793 – RT: 15.8 min – ESI-MS (*m/z*): 985.28886 $[M+2H]^{2+}$, 670.8729 $[M+H+2Na]^{3+}$, 657.1962 $[M+3H]^{3+}$

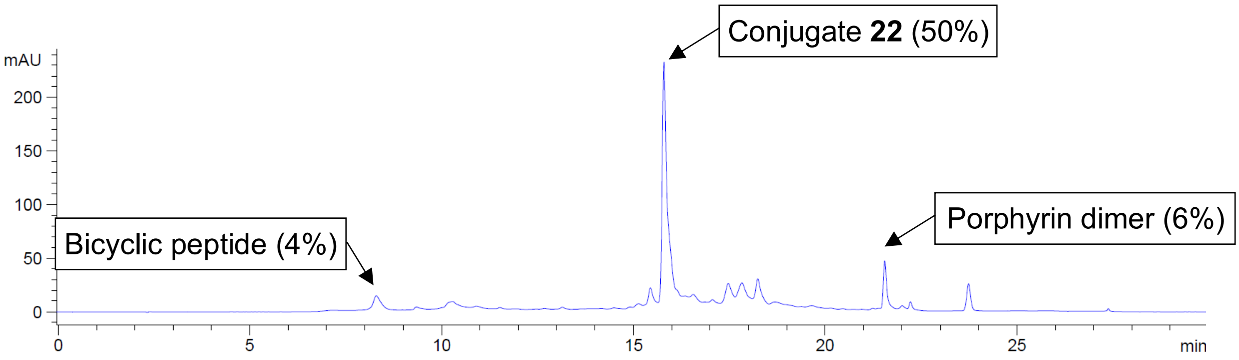

*Figure S65. Analytical HPLC of crude conjugate **22**.*

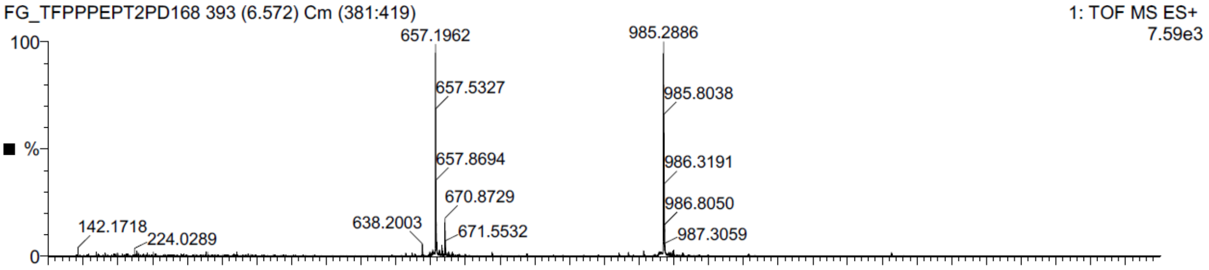

*Figure S66. TIC from TOF MS ES+ analysis of crude conjugate **22**.*

Conjugate 23

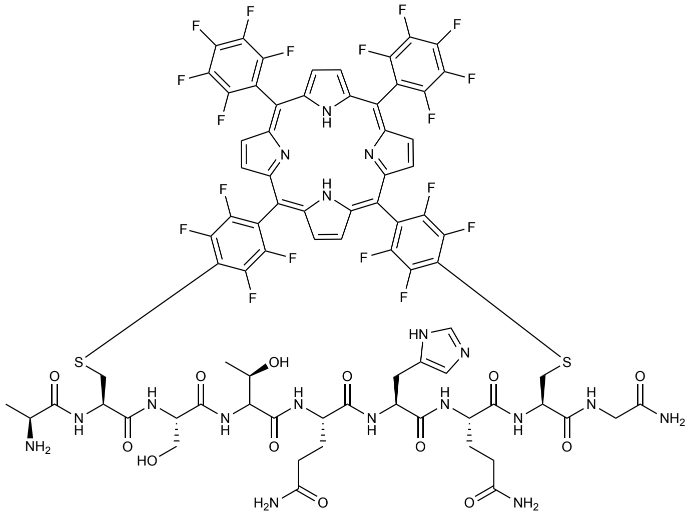

Calculated Exact Mass: 1866.4054 – Molecular Weight: 1867.5743 – RT: 18.1 min – ESI-MS (*m/z*): 934.7472 $[M+2H]^{2+}$, 636.8464 $[M+H+2Na]^{3+}$, 623.4749 $[M+3H]^{3+}$– Isolated yield: 24%

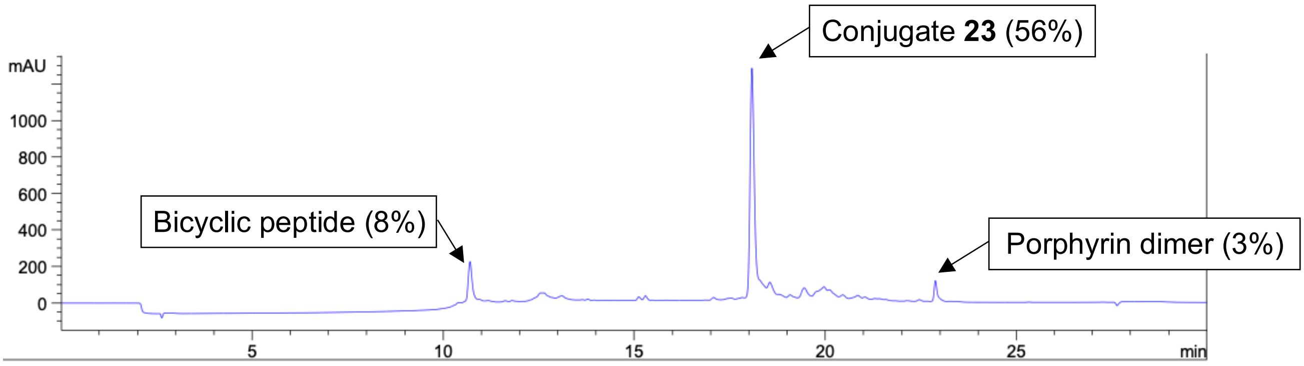

*Figure S67. Analytical HPLC of crude conjugate **23**.*

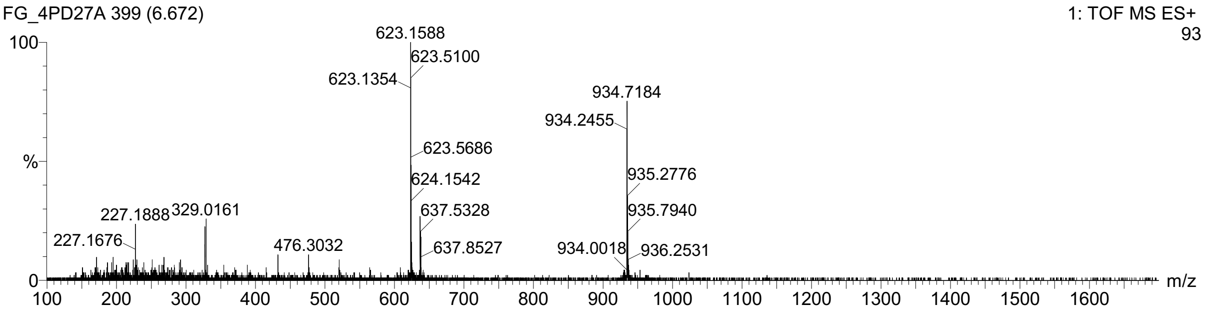

*Figure S68. TIC from TOF MS ES+ analysis of crude conjugate **23**.*

Conjugate 24

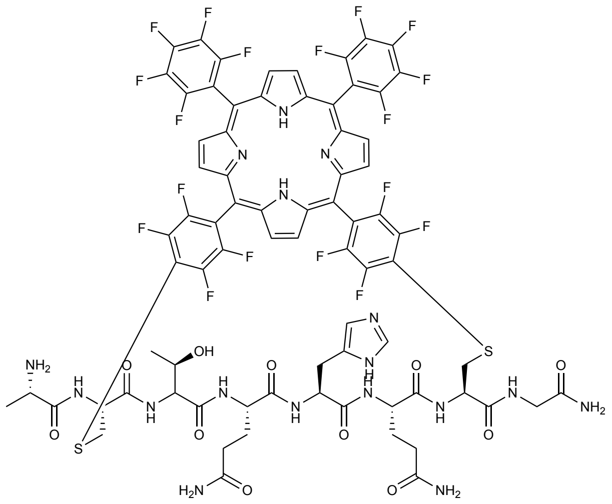

Calculated Exact Mass: 1799.3734 – Molecular Weight: 1780.4963 – RT: 18.3 min – ESI-MS (*m/z*): 891.0790 $[M+2H]^{2+}$, 608.4000 $[M+H+2Na]^{3+}$, 594.3957 $[M+3H]^{3+}$– Isolated yield: 25%

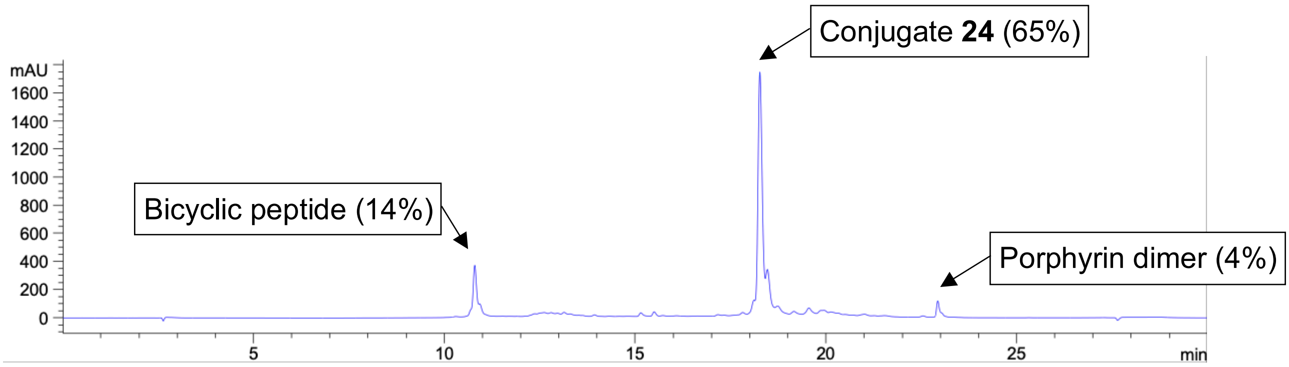

*Figure S69. Analytical HPLC of crude conjugate **24**.*

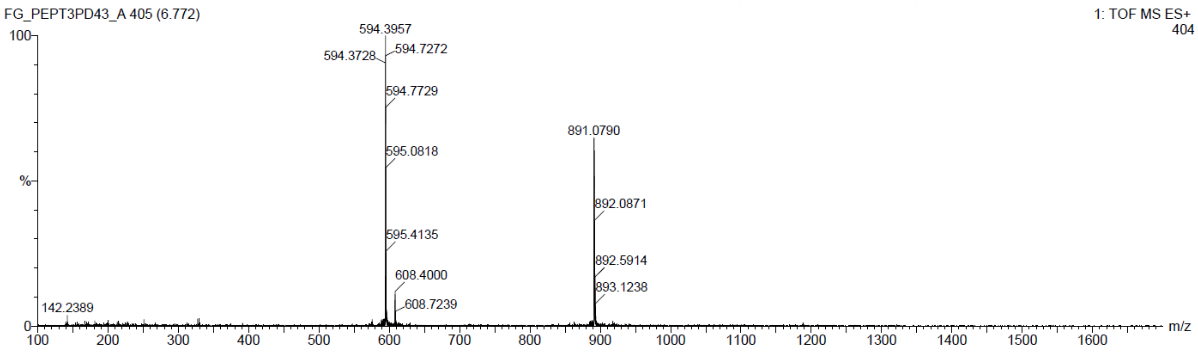

*Figure S70. TIC from TOF MS ES+ analysis of crude conjugate **24**.*

Conjugate 25

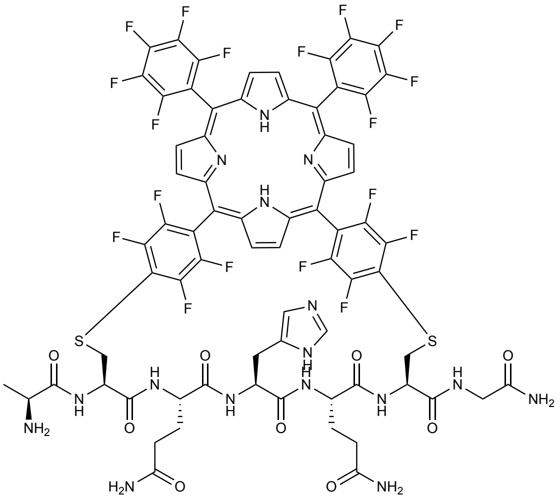

Calculated Exact Mass: 1678.3257 – Molecular Weight: 1679.3913 – RT: 16.4 min – ESI-MS (*m/z*): 1679.8125 $[M+H]^{+}$, 840.3854 $[M+2H]^{2+}$, 574.2904 $[M+H+2Na]^{3+}$– Isolated yield: 39%

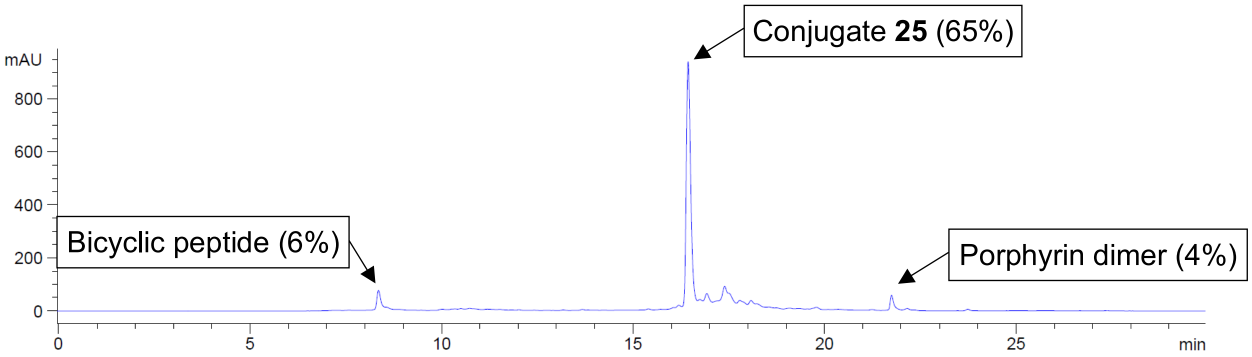

*Figure S71. Analytical HPLC of crude conjugate **25**.*

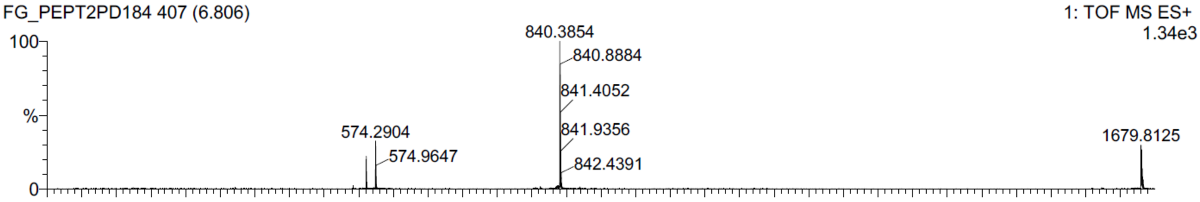

*Figure S72. TIC from TOF MS ES+ analysis of crude conjugate **25**.*

Conjugate 26

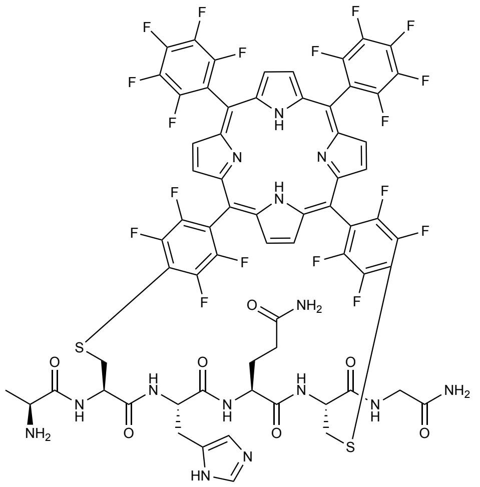

Calculated Exact Mass: 1550.2671 – Molecular Weight: 1551.2603 – RT: 17.1 min – ESI-MS (*m/z*): 1552.0144 $[M+H]^+$, 776.4614 $[M+2H]^{2+}$, 532.0419 $[M+H+2Na]^{3+}$, 518.0013 $[M+3H]^{3+}$

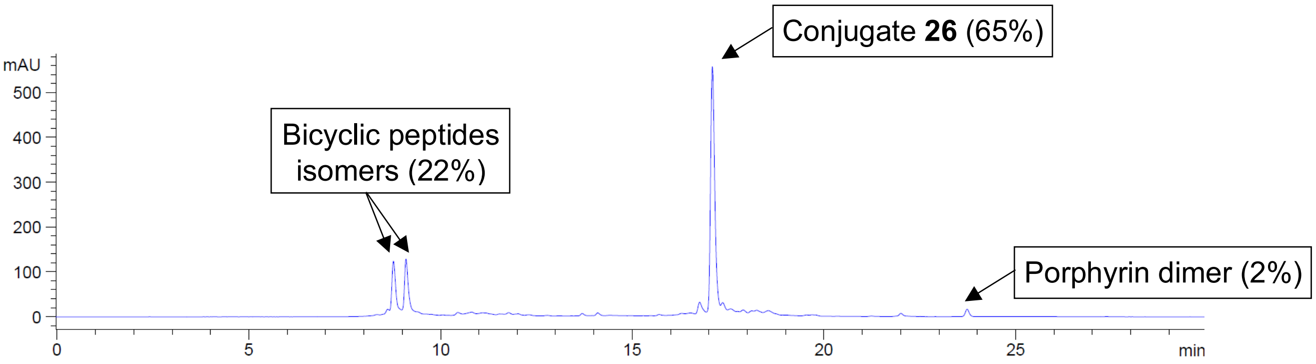

*Figure S73. Analytical HPLC of crude conjugate **26**.*

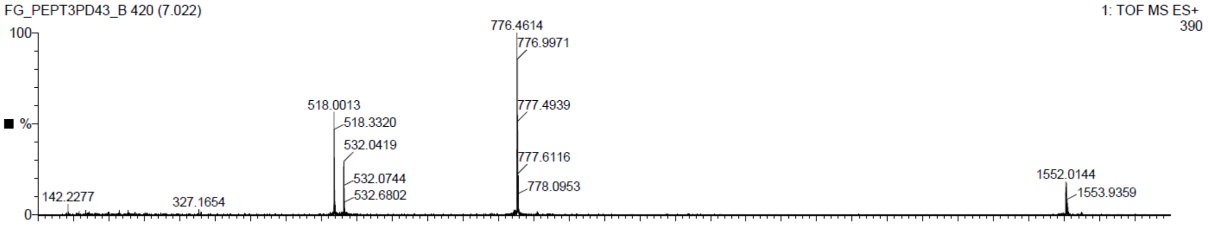

*Figure S74. TIC from TOF MS ES+ analysis of crude conjugate **26**.*

Conjugate 27

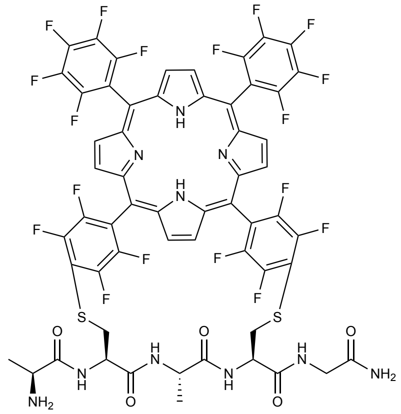

Calculated Exact Mass: 1356.1868 – Molecular Weight: 1357.0663 – RT: 19.9 min – ESI-MS (*m/z*): 1357.5035 $[M+H]^+$, 905.7087 $[2M+3H]^{3+}$, 679.2785 $[M+2H]^{2+}$

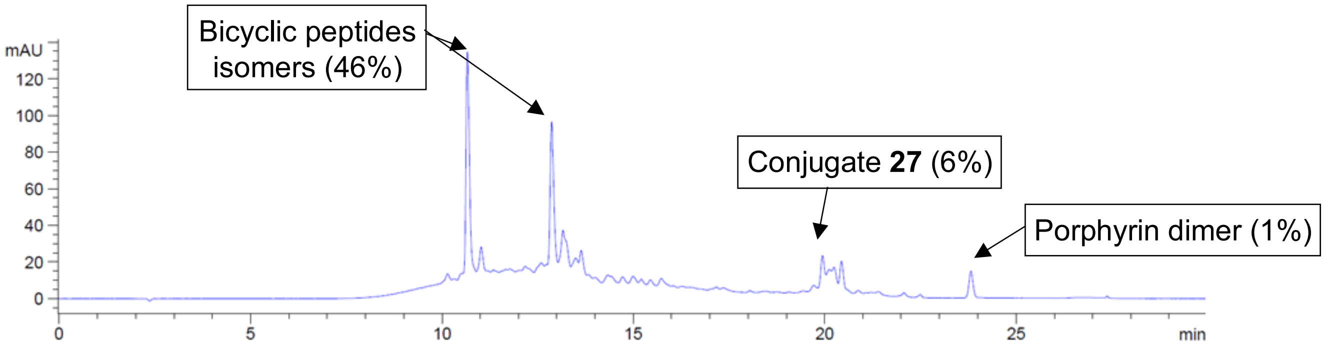

*Figure S75. Analytical HPLC of crude conjugate **27**.*

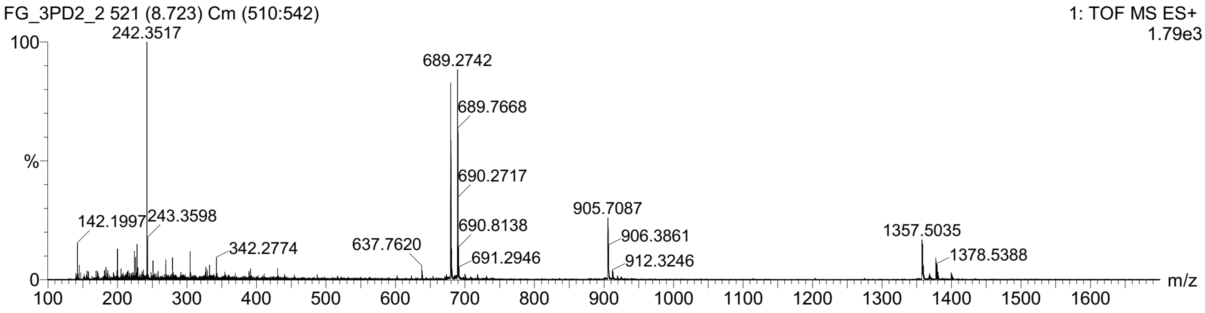

*Figure S76. TIC from TOF MS ES+ analysis of crude conjugate **27**.*

Conjugate 28

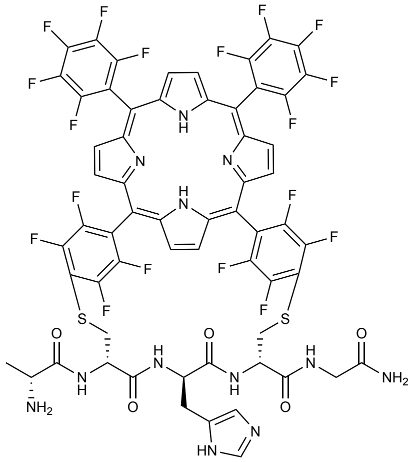

Calculated Exact Mass: 1422.2086 – Molecular Weight: 1423.1293 – RT: 18.5 min – ESI-MS (*m/z*): 1423.8884 $[M+H]^{+}$, 712.4102 $[M+2H]^{2+}$, 488.9788 $[M+H+2Na]^{3+}$, 475.2771 $[M+3H]^{3+}$

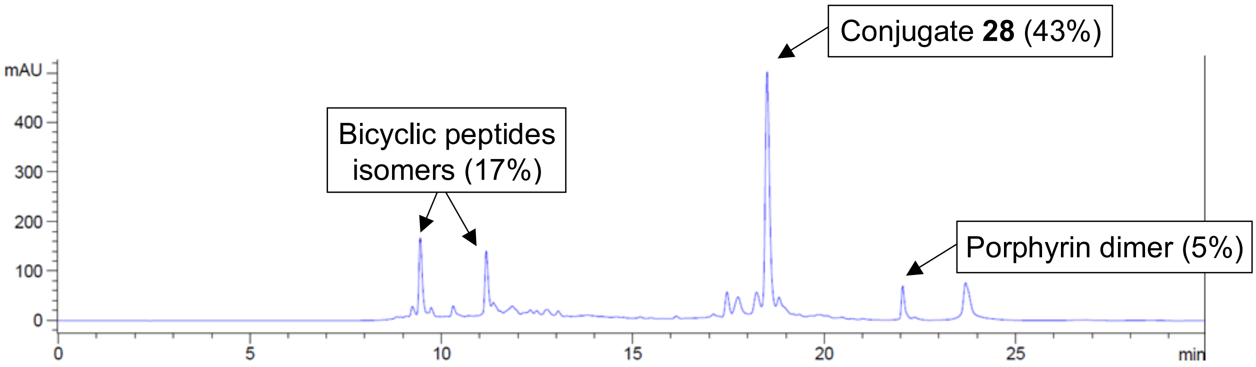

*Figure S77. Analytical HPLC of crude conjugate **28**.*

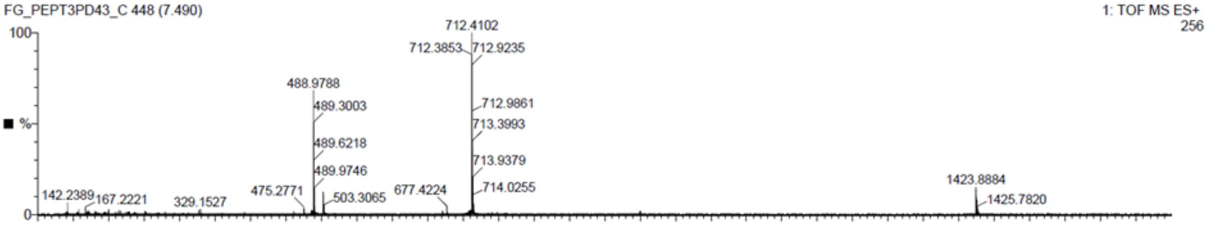

*Figure S78. TIC from TOF MS ES+ analysis of crude conjugate **28**.*

Conjugate 29

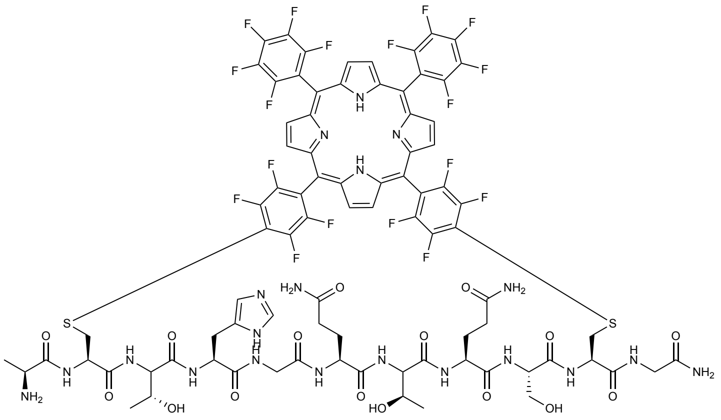

Calculated Exact Mass: 2024.4746 – Molecular Weight: 2025.7313 – RT: 15.6 min – ESI-MS (*m/z*): 1014.0622 $[M+2H]^{2+}$, 690.0623 $[M+H+2Na]^{3+}$, 676.3733 $[M+3H]^{3+}$– Isolated yield: 10%

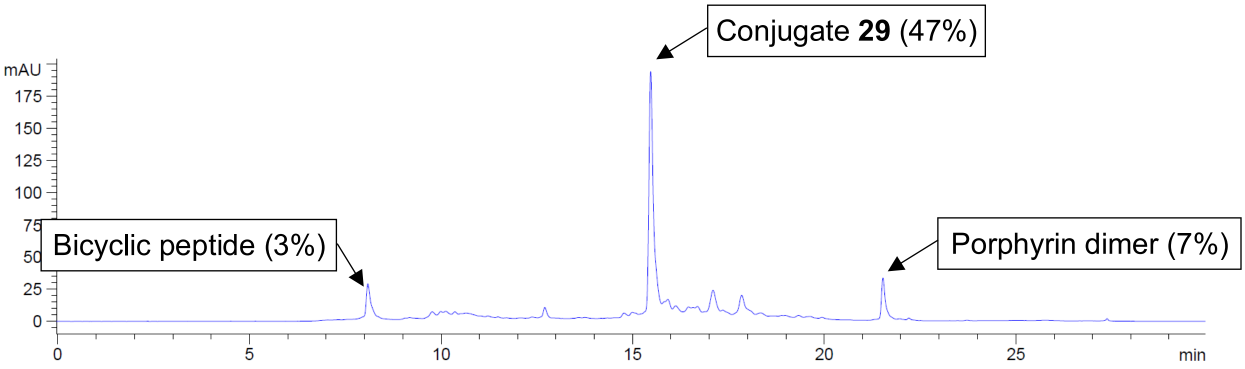

*Figure S79. Analytical HPLC of crude conjugate **29**.*

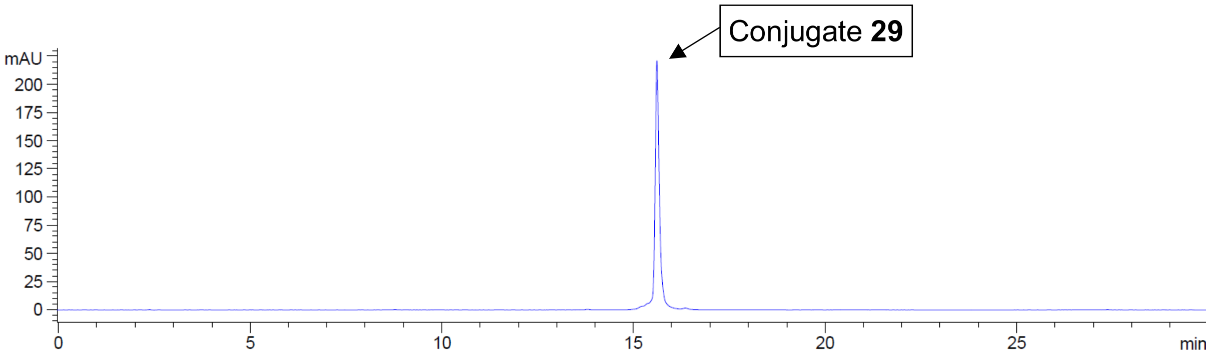

*Figure S80. Analytical HPLC of purified conjugate **29**.*

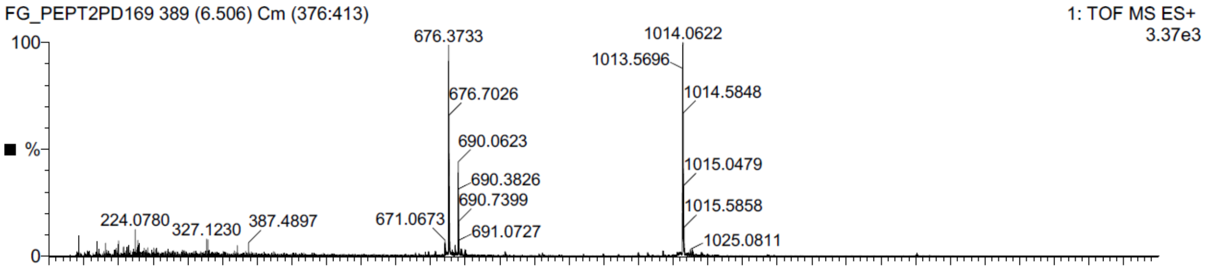

*Figure S81. TIC from TOF MS ES+ analysis of crude conjugate **29**.*

Conjugate 30

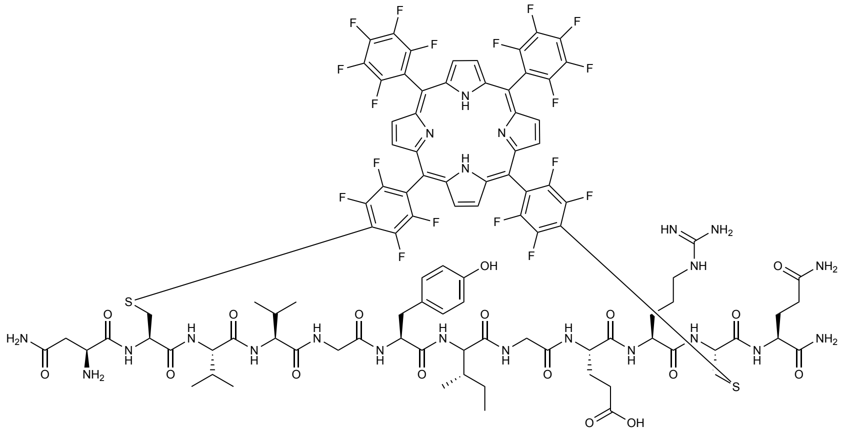

Calculated Exact Mass: 2272.6634 – Molecular Weight: 2274.1013 – RT: 16.1 min – ESI-MS (*m/z*): 1137.8462 $[M+2H]^{2+}$, 758.9305 $[M+3H]^{3+}$

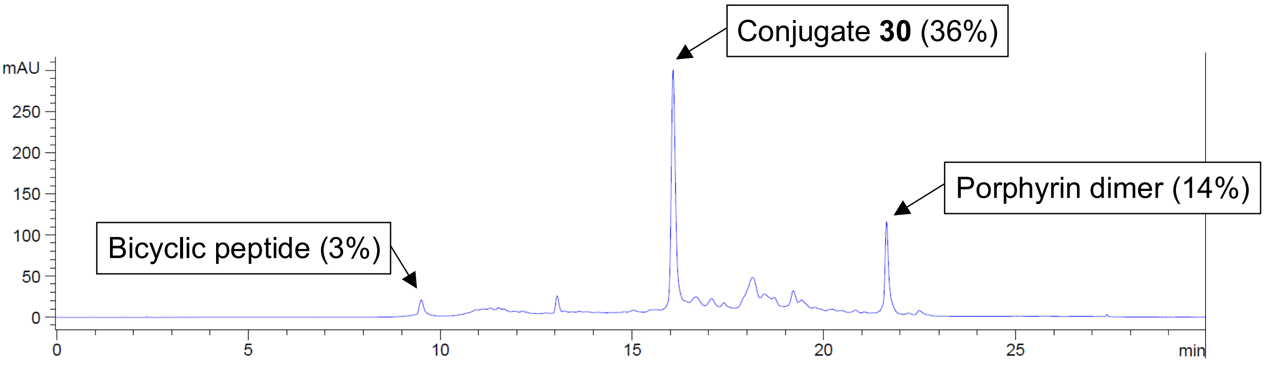

*Figure S82. Analytical HPLC of crude conjugate **30**.*

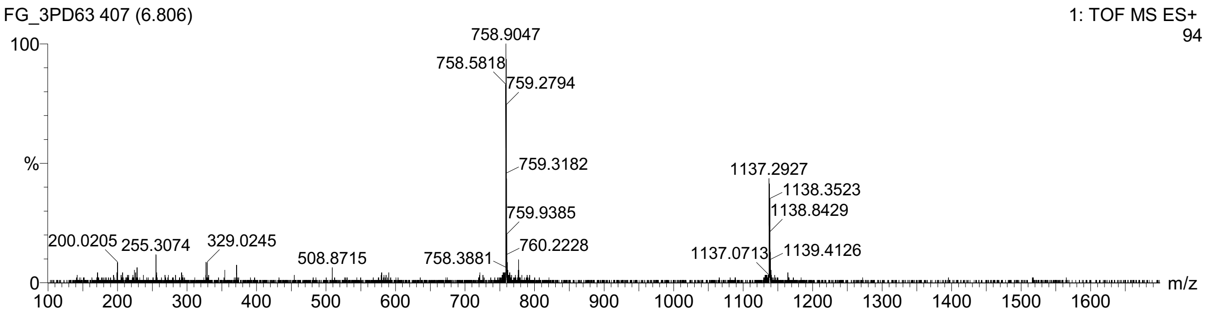

*Figure S83. TIC from TOF MS ES+ analysis of crude conjugate **30**.*

Conjugate 31

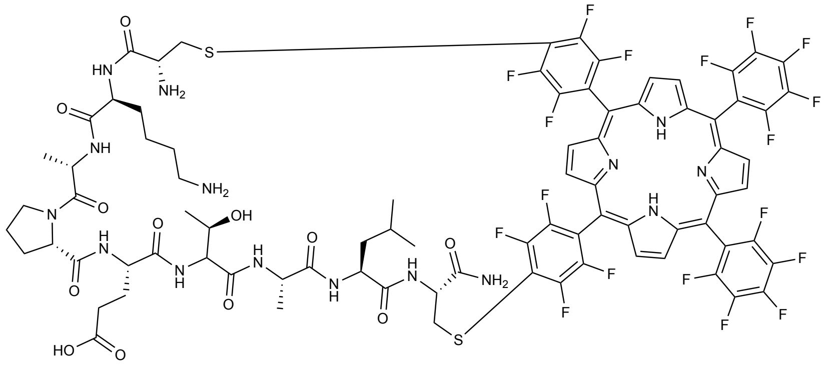

Calculated Exact Mass: 1867.4874 – Molecular Weight: 1868.6863 – RT: 17.1 min – ESI-MS (*m/z*): 935.3064 $[M+2H]^{2+}$, 637.5211 $[M+H+2Na]^{3+}$, 623.5334 $[M+3H]^{3+}$

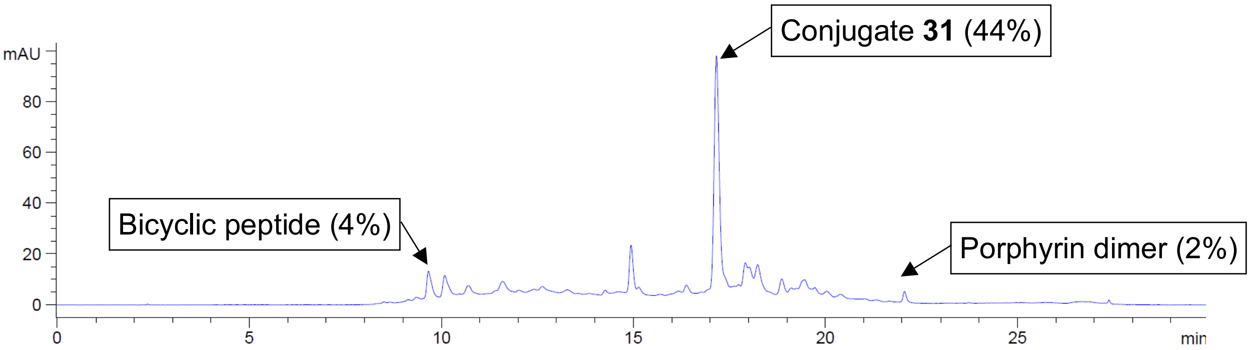

*Figure S84. Analytical HPLC of crude conjugate **31**.*

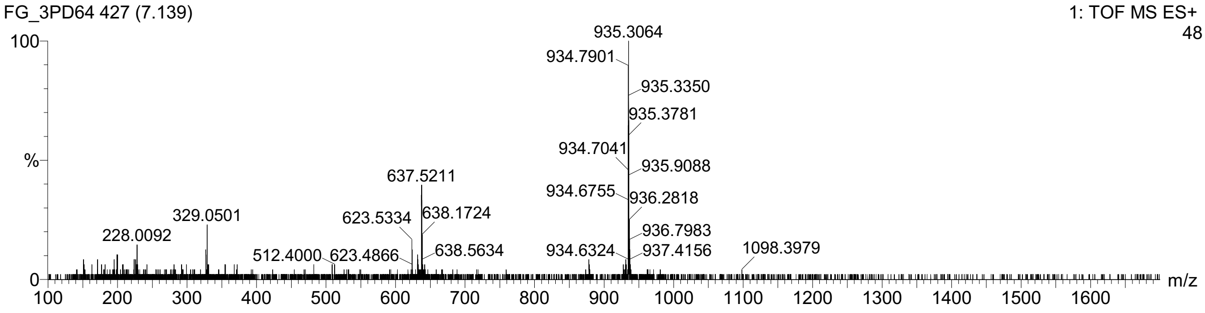

*Figure S85. TIC from TOF MS ES+ analysis of crude conjugate **31**.*

Conjugate 32

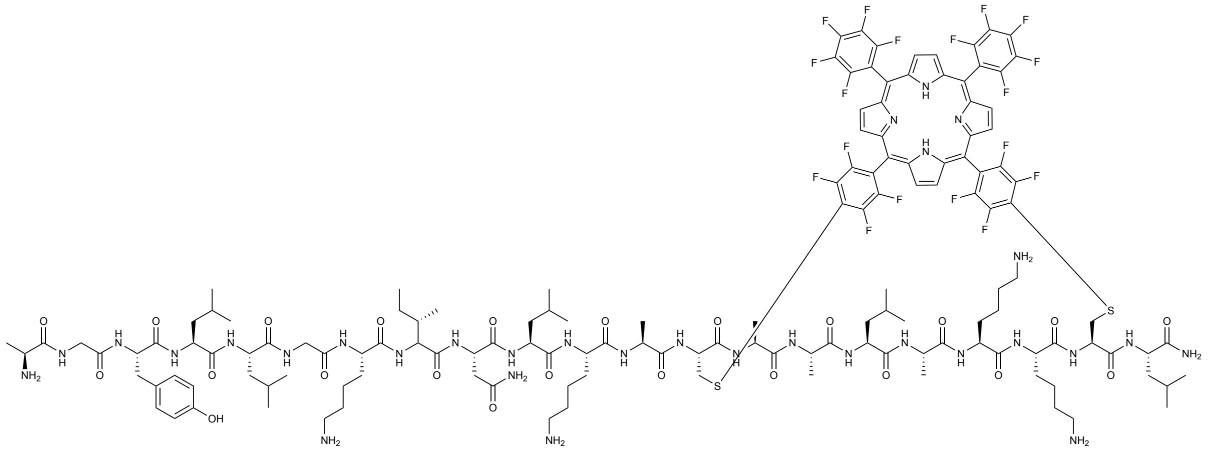

Calculated Exact Mass: 3094.31 – Molecular Weight: 3096.30 – RT: 16.9 min – ESI-MS (*m/z*): 1549.1902 $[M+2H]^{2+}$, 1032.8458 $[M+3H]^{3+}$, 744.8635 $[M+4H]^{4+}$, 617.0869 $[M+5H]^{5+}$

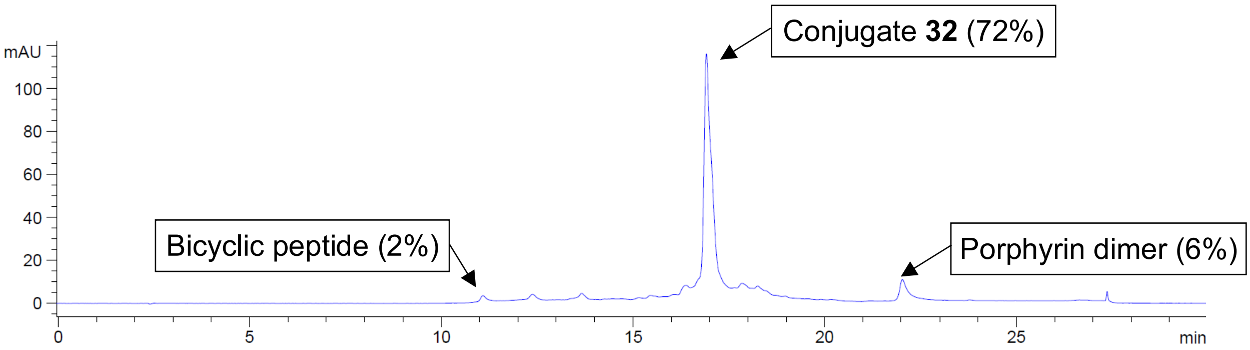

*Figure S86. Analytical HPLC of crude conjugate **32**.*

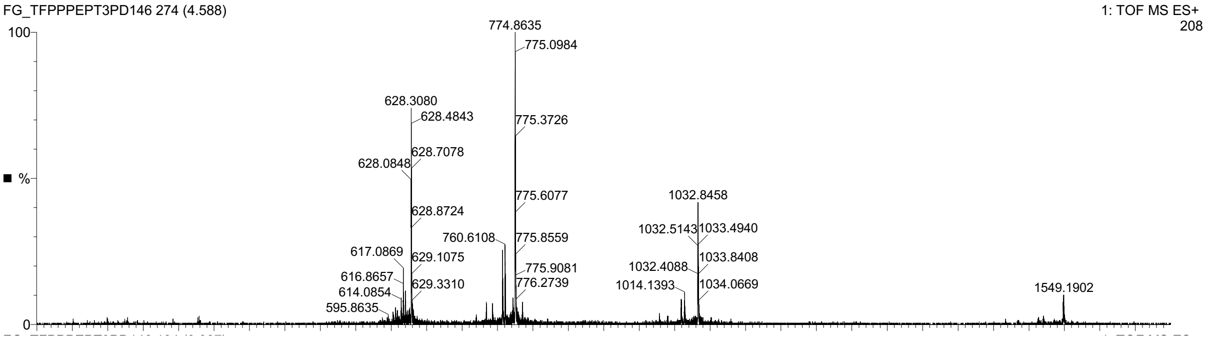

*Figure S87. TIC from TOF MS ES+ analysis of crude conjugate **32**.*

Conjugate 34

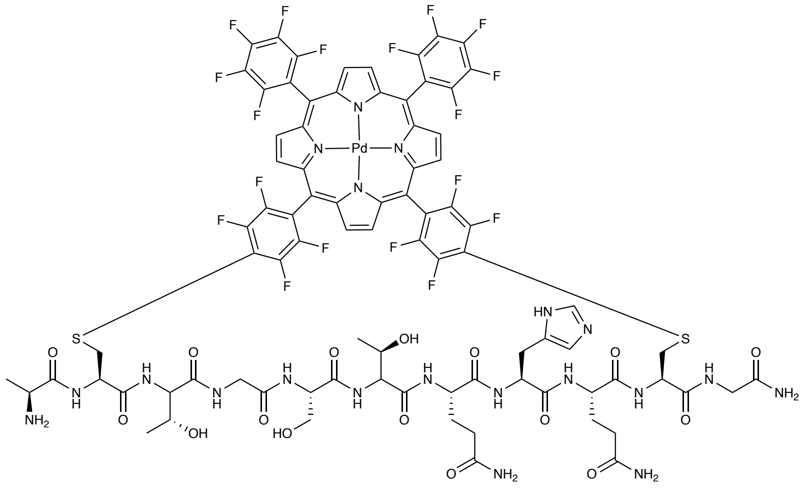

Calculated Exact Mass: 2128.3624 – Molecular Weight: 2130.1353 – RT: 18.3 min – ESI-MS (*m/z*): 1065.2397 $[M+2H]^{2+}$, 725.1728 $[M+H+2Na]^{3+}$, 710.5242 $[M+3H]^{3+}$

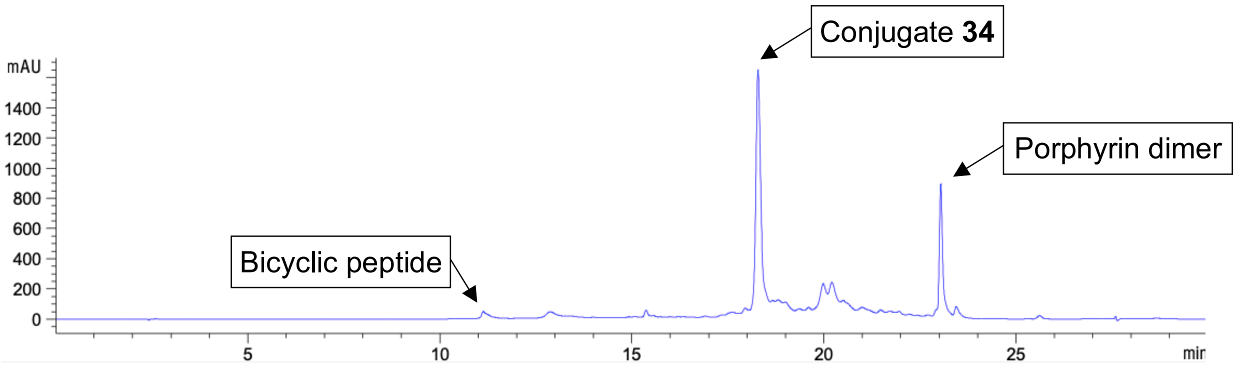

*Figure S88. Analytical HPLC of crude conjugate **34**.*

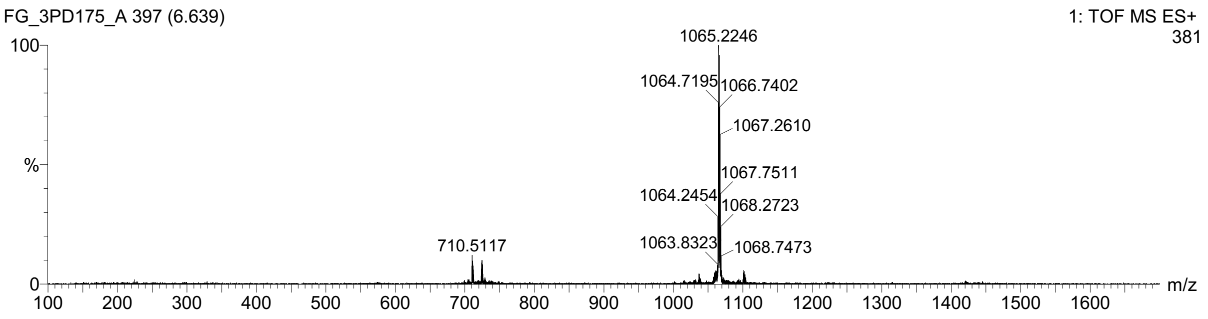

*Figure S89. TIC from TOF MS ES+ analysis of crude conjugate **34**.*

Conjugate 35

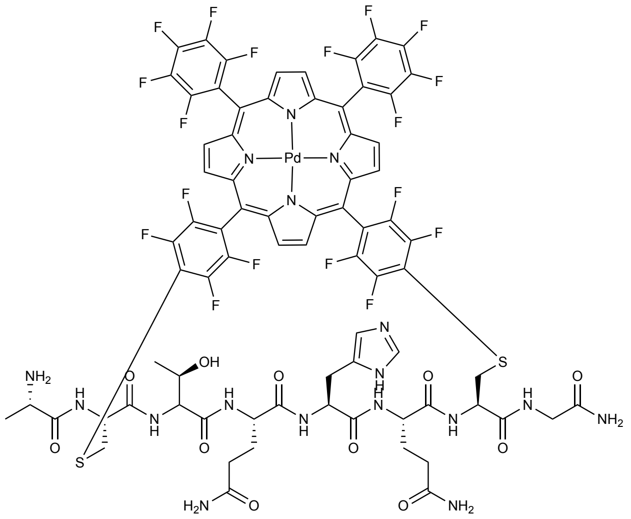

Calculated Exact Mass: 1883.2612 – Molecular Weight: 1884.9003 – RT: 18.8 min – ESI-MS (*m/z*): 942.6487 $[M+2H]^{2+}$

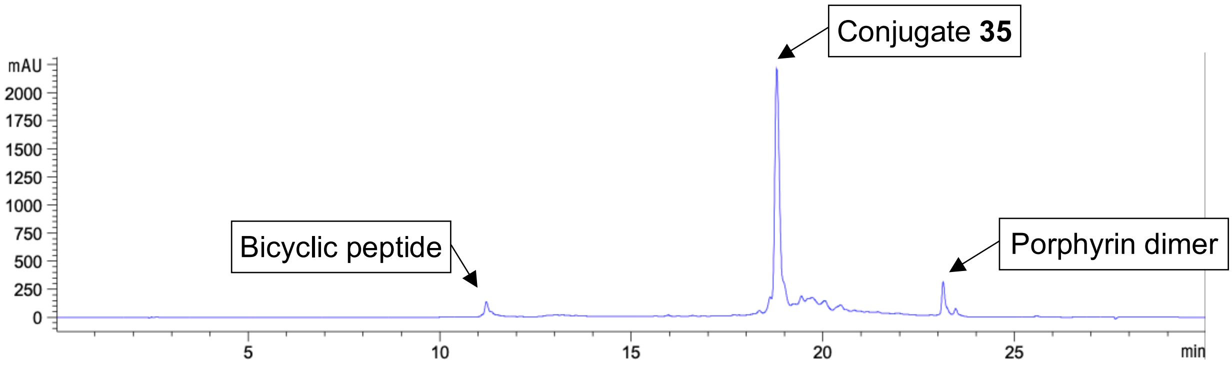

*Figure S90. Analytical HPLC of crude conjugate **35**.*

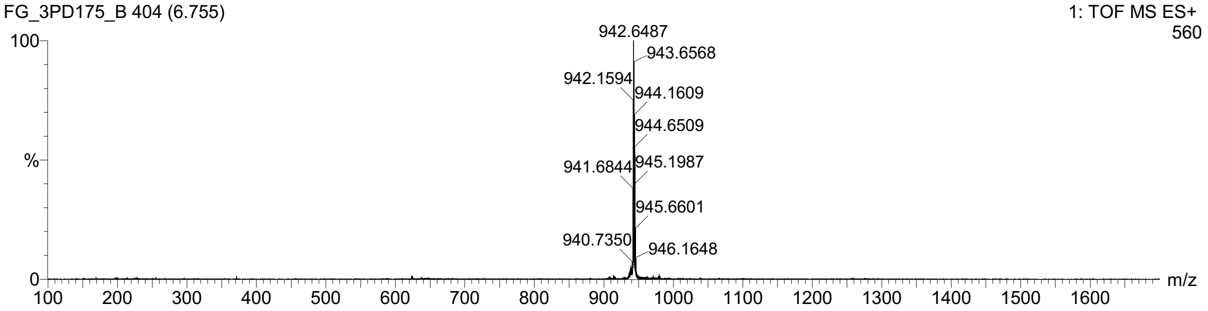

*Figure S91. TIC from TOF MS ES+ analysis of crude conjugate **35**.*

Conjugate 36

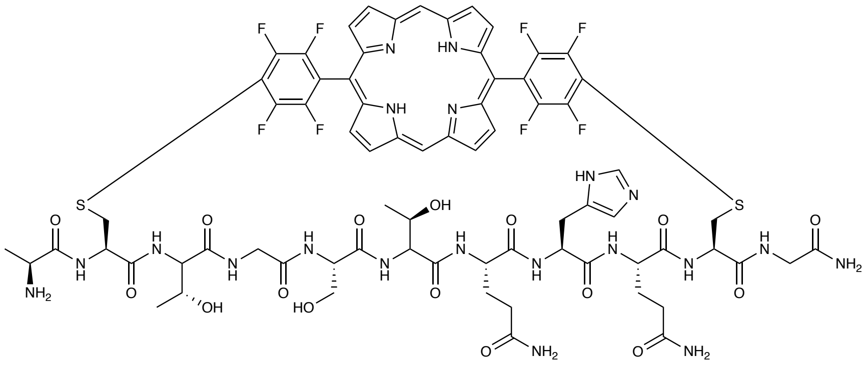

Calculated Exact Mass: 1692.5062 – Molecular Weight: 1693.6312 – RT: 10.3 min – ESI-MS (*m/z*): 847.5228 $[M+2H]^{2+}$, 579.0417 $[M+H+2Na]^{3+}$, 565.3703 $[M+3H]^{3+}$

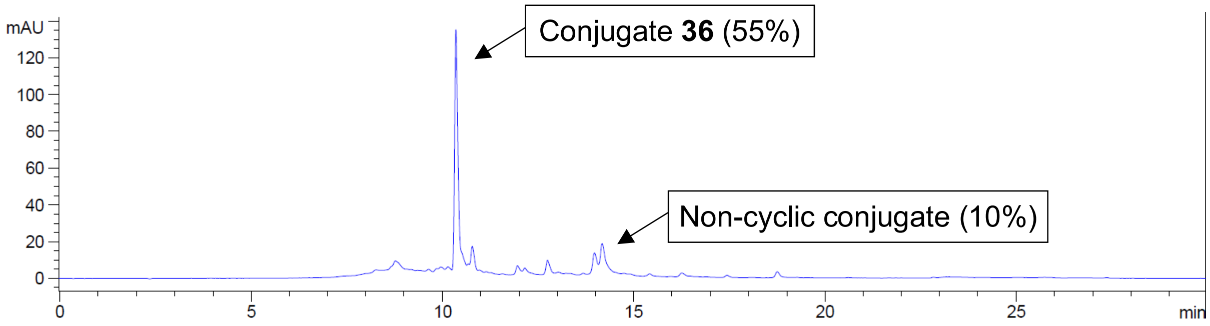

*Figure S92. Analytical HPLC of crude conjugate **36**.*

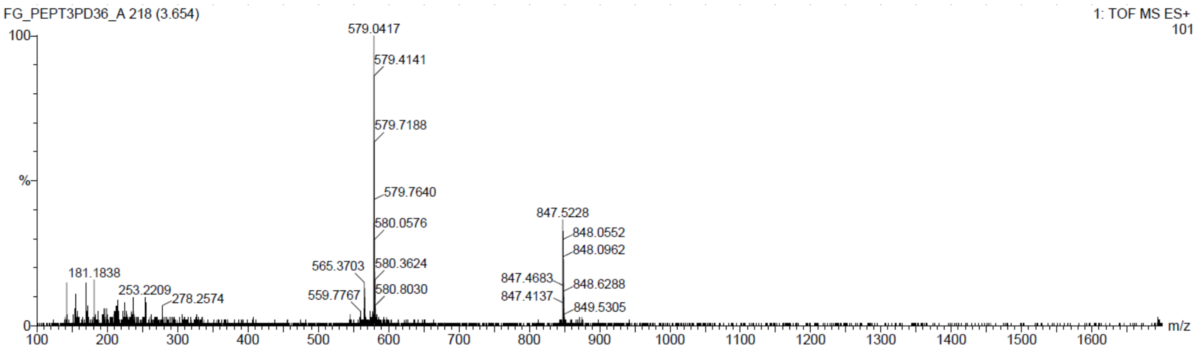

*Figure S93. TIC from TOF MS ES+ analysis of crude conjugate **36**.*

Conjugate 37

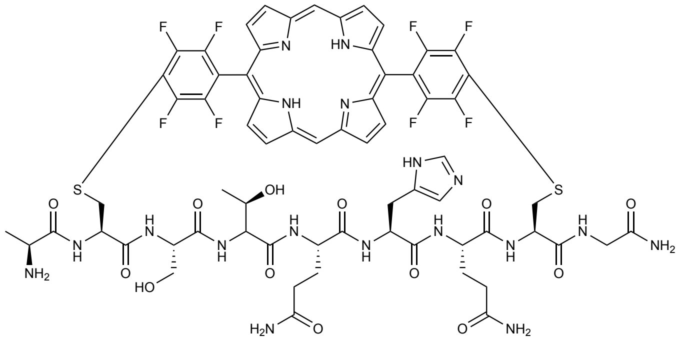

Calculated Exact Mass: 1534.4370 - Molecular Weight: 1535.4742 – RT: 11.1 min – ESI-MS (*m/z*): 1536.3446 $[M+H]^+$, 768.7196 $[M+2H]^{2+}$, 526.4764 $[M+H+2Na]^{3+}$

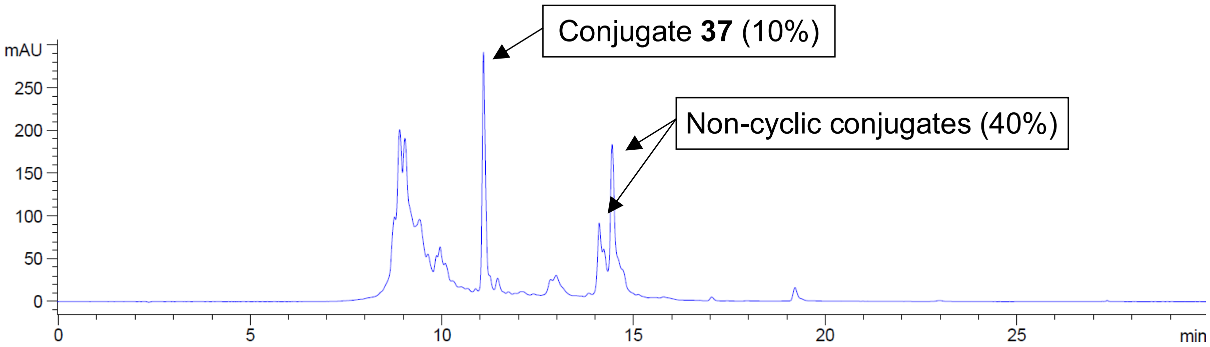

*Figure S94. Analytical HPLC of crude conjugate **37**.*

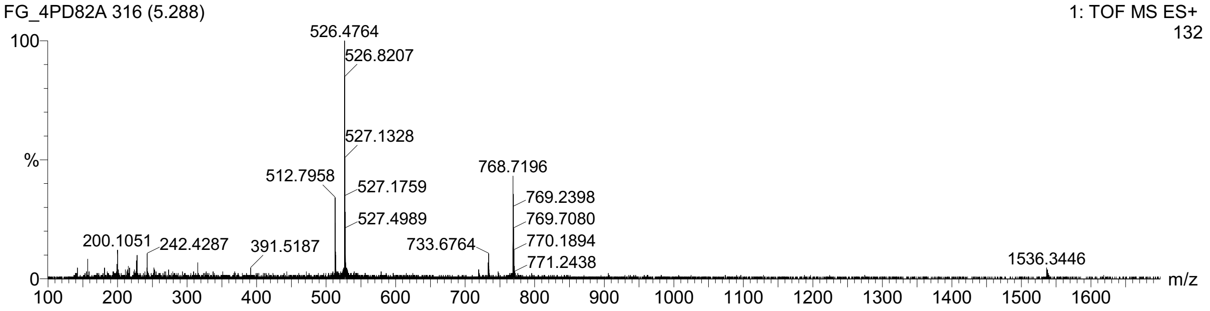

*Figure S95. TIC from TOF MS ES+ analysis of crude conjugate **37**.*

Conjugate 38

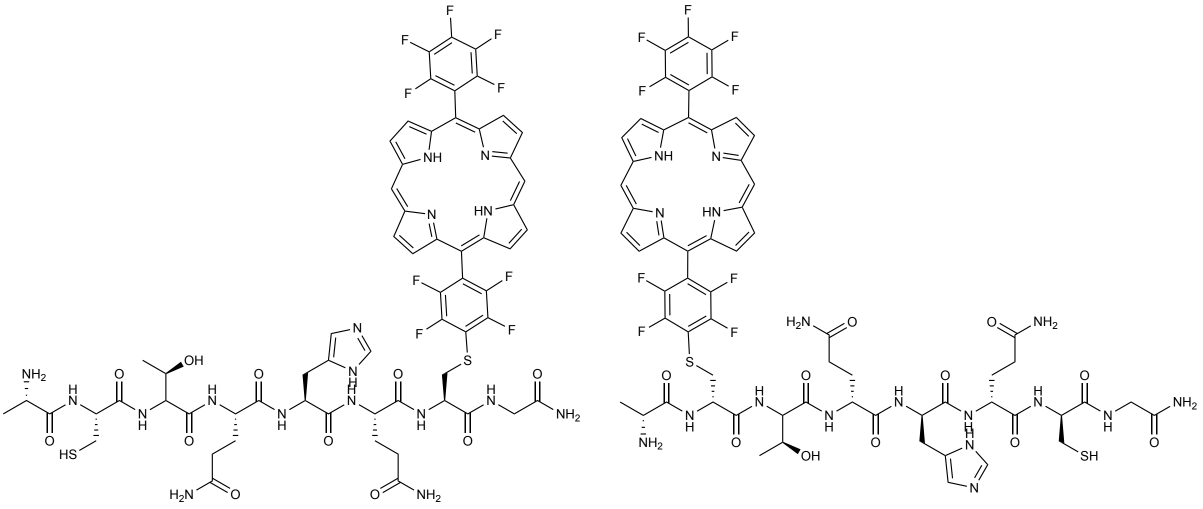

Calculated Exact Mass: 1467.4112 – Molecular Weight: 1468.4026 – RT: 14.0/14.5 min – ESI-MS (*m/z*): 1469.0731 $[M+H]^{+}$, 735.0255 $[M+2H]^{2+}$, 490.6806 $[M+3H]^{3+}$

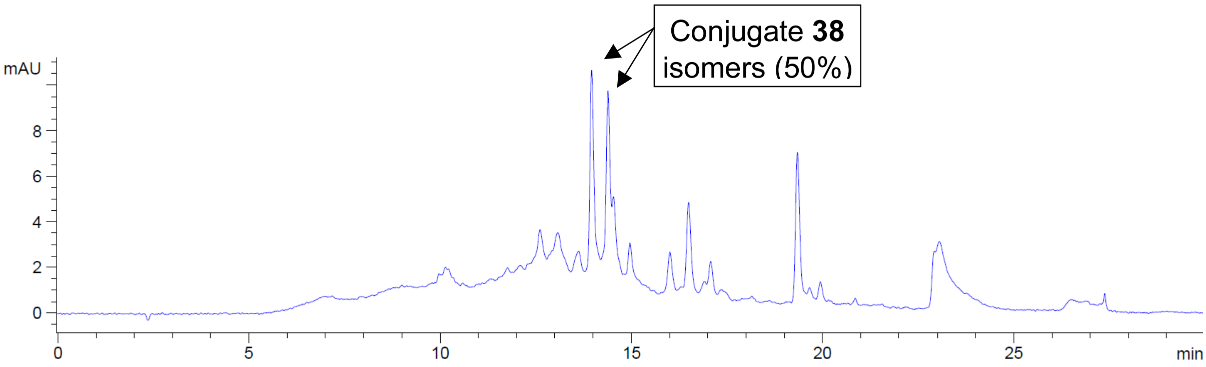

*Figure S96. Analytical HPLC of crude conjugate **38**.*

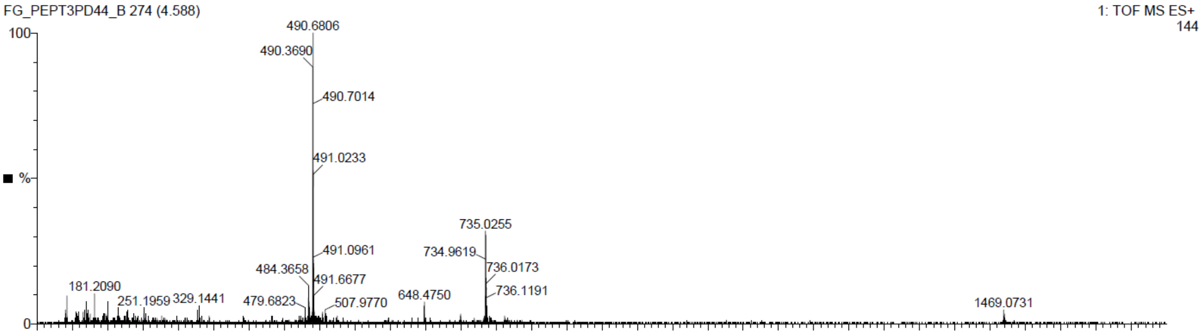

*Figure S97. TIC from TOF MS ES+ analysis of crude conjugate **38**.*

Conjugate 39

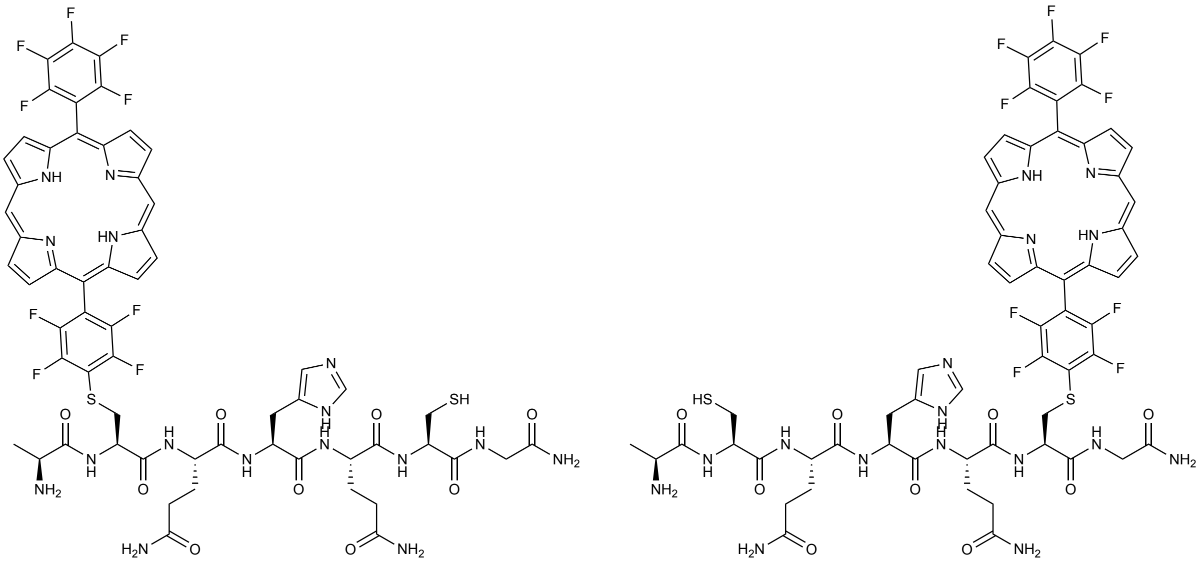

Calculated Exact Mass: 1366.3636 – Molecular Weight: 1367.2976 – RT: 13.9/14.4 min – ESI-MS (*m/z*): 1367.9575 $[M+H]^+$, 684.4943 $[M+2H]^{2+}$, 456.6762 $[M+3H]^{3+}$

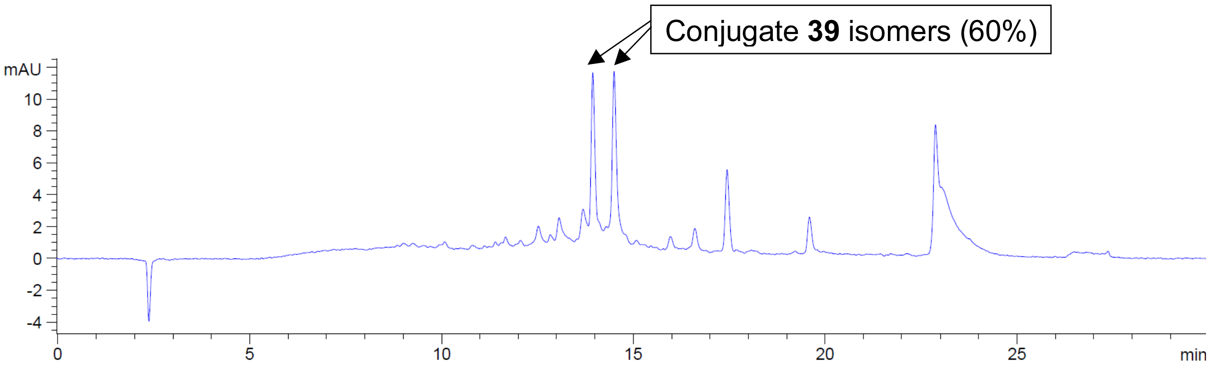

*Figure S98. Analytical HPLC of crude conjugate **39**.*

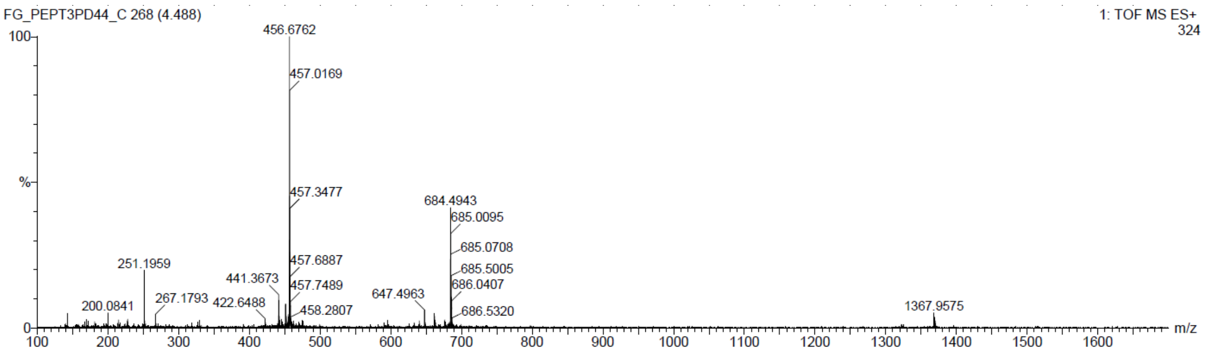

*Figure S99. TIC from TOF MS ES+ analysis of crude conjugate **39**.*

Conjugate 40

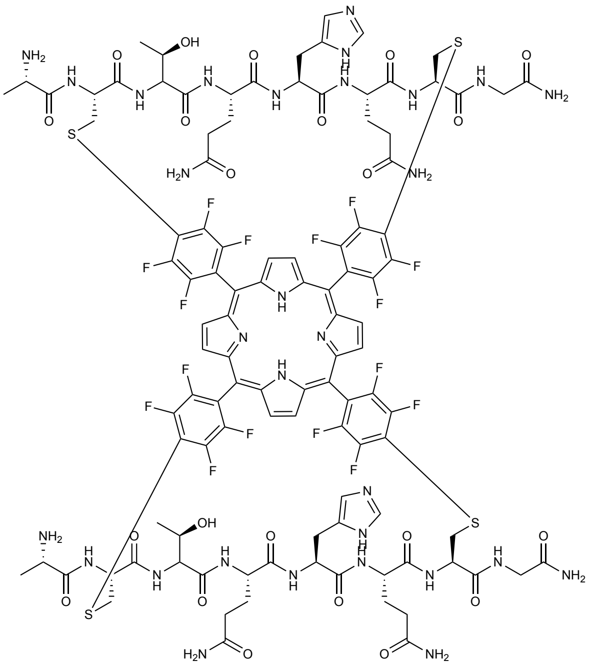

Calculated Exact Mass: 2584.6882 – Molecular Weight: 2586.4325 – RT: 8.4 min – ESI-MS (*m/z*): 1293.8453 $[M+2H]^{2+}$, 863.3235 $[M+3H]^{3+}$, 658.0114 $[M+3H+Na]^{4+}$, 647.7198 $[M+4H]^{4+}$– Isolated yield: 27%

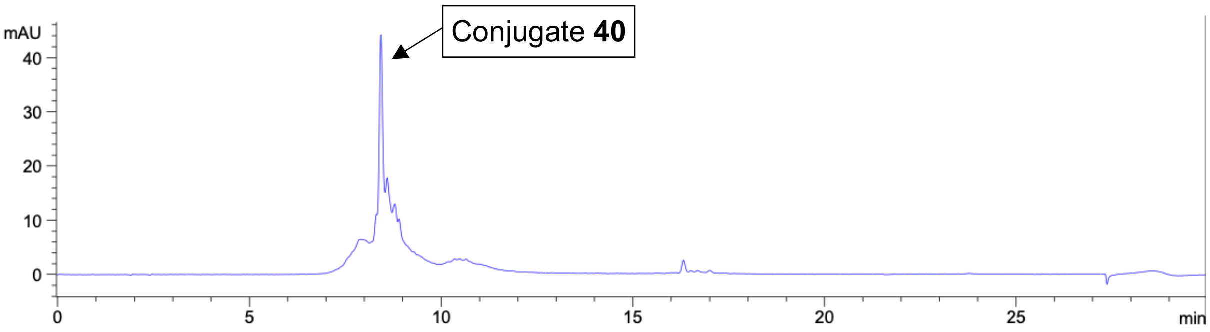

*Figure S100. Analytical HPLC of crude conjugate **40**.*

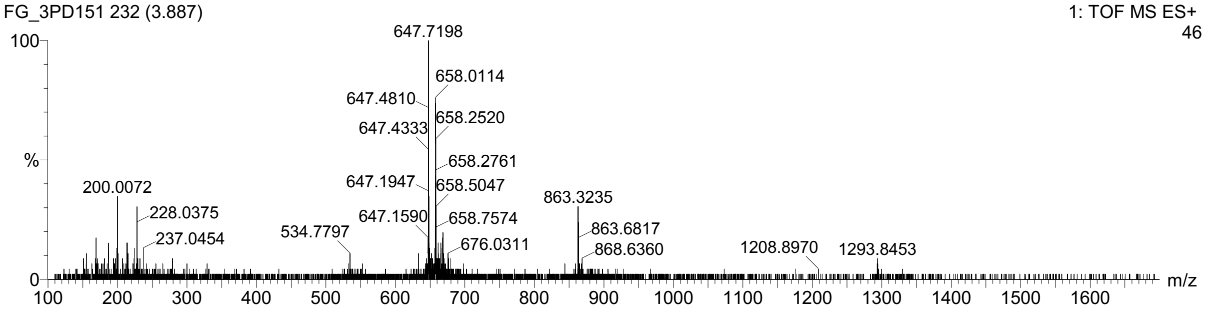

*Figure S101. TIC from TOF MS ES+ analysis of crude conjugate **40**.*

Conjugate 41

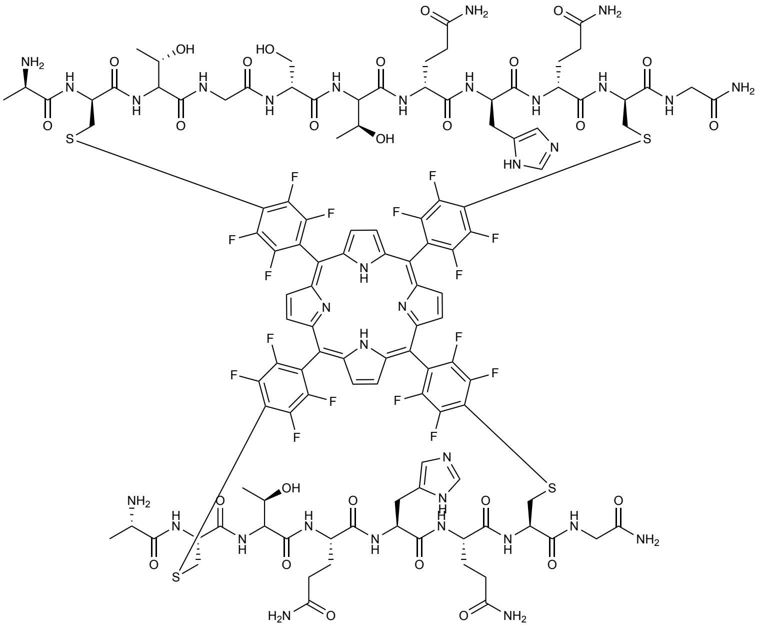

Calculated Exact Mass: 2829.7893 – Molecular Weight: 2831.6675 – RT: 10.6 min – ESI-MS (*m/z*): 944.9680 $[M+3H]^{3+}$, 708.9626 $[M+4H]^{4+}$

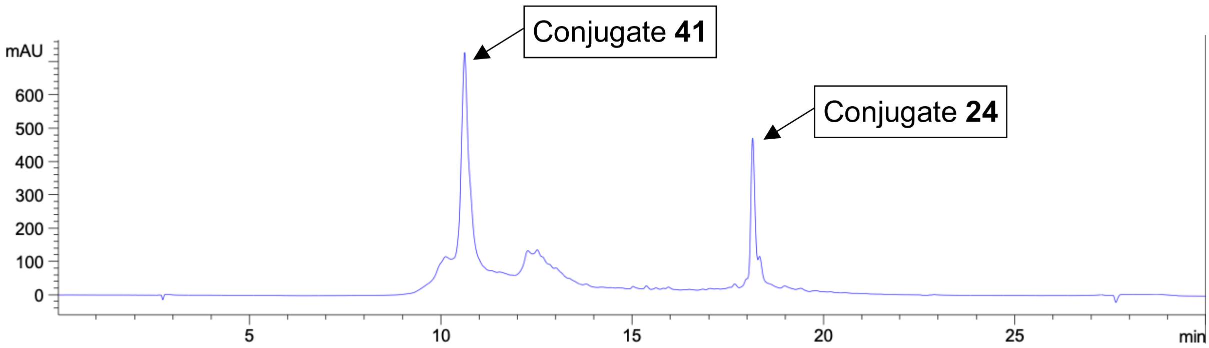

*Figure S102. Analytical HPLC of crude conjugate **41**.*

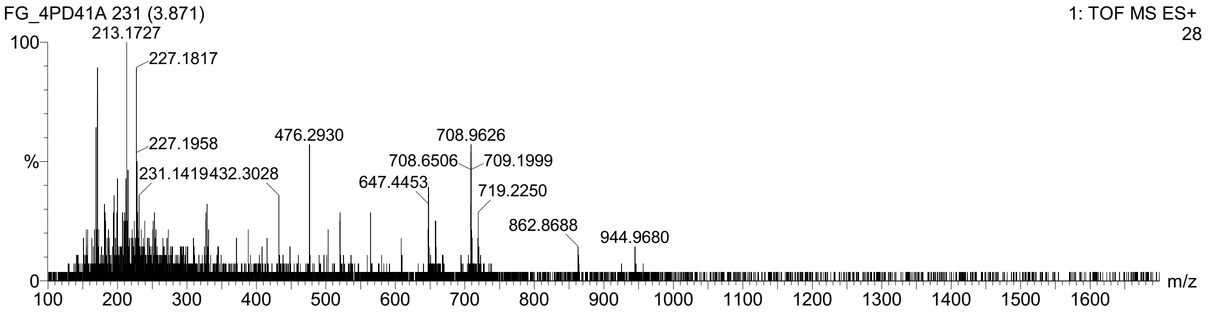

*Figure S103. TIC from TOF MS ES+ analysis of crude conjugate **41**.*

Conjugate 42

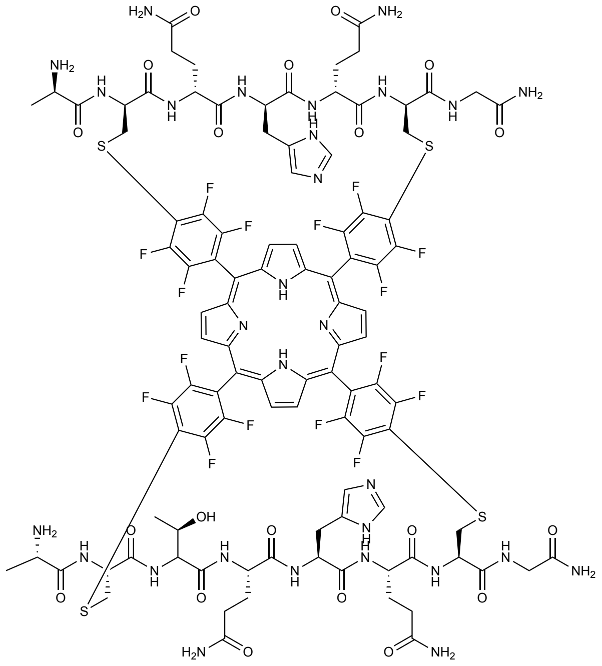

Calculated Exact Mass: 2483.6405 – Molecular Weight: 2485.3275 – RT: 10.6 min – ESI-MS (*m/z*): 829.1957 $[M+3H]^{3+}$

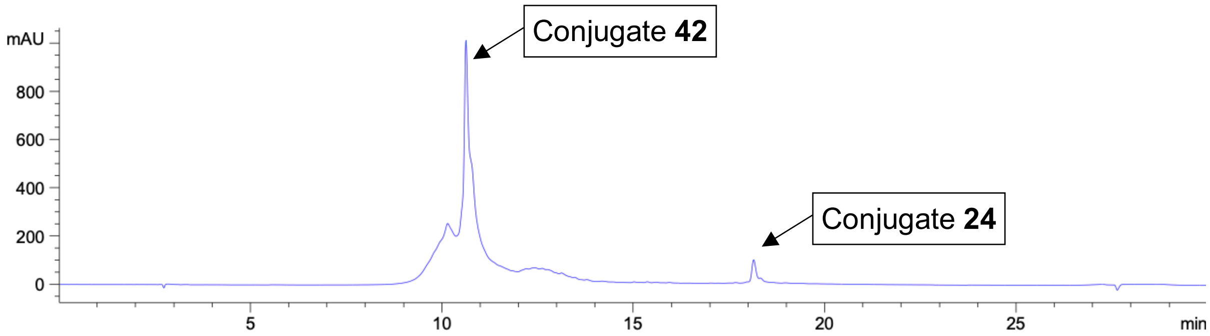

*Figure S104. Analytical HPLC of crude conjugate **42**.*

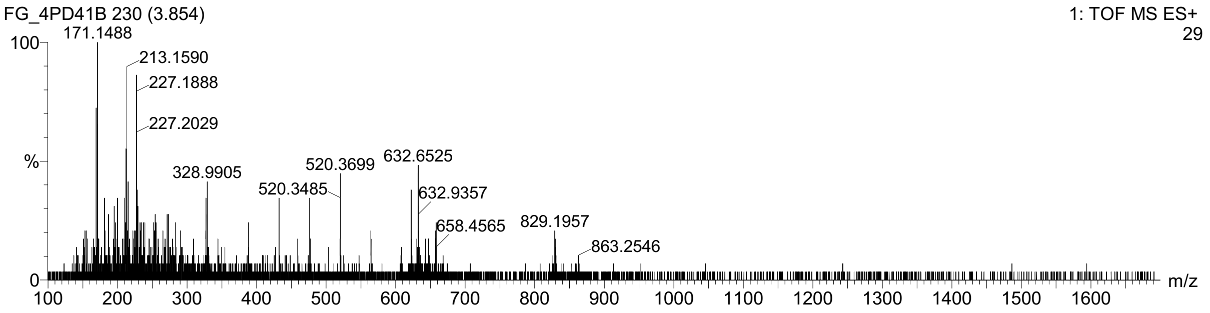

*Figure S105. TIC from TOF MS ES+ analysis of crude conjugate **42**.*

Conjugate 46

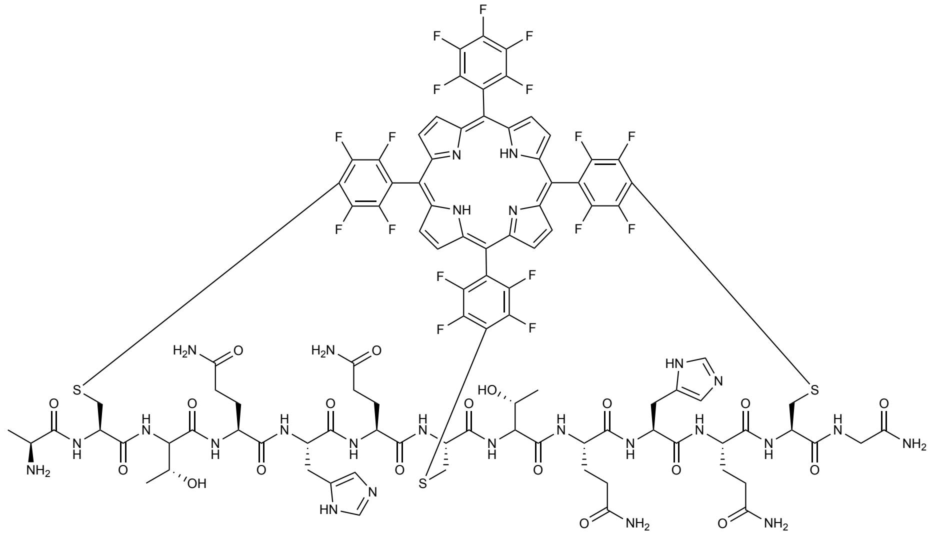

Calculated Exact Mass: 2356.6001 – Molecular Weight: 2358.1379 – RT: 14.1 min – ESI-MS (*m/z*): 1179.8256 $[M+2H]^{2+}$, 786.9054 $[M+3H]^{3+}$, 610.9289 $[M+2H+2Na]^{4+}$, 600.4277 $[M+3H+Na]^{4+}$– Isolated yield: 15%

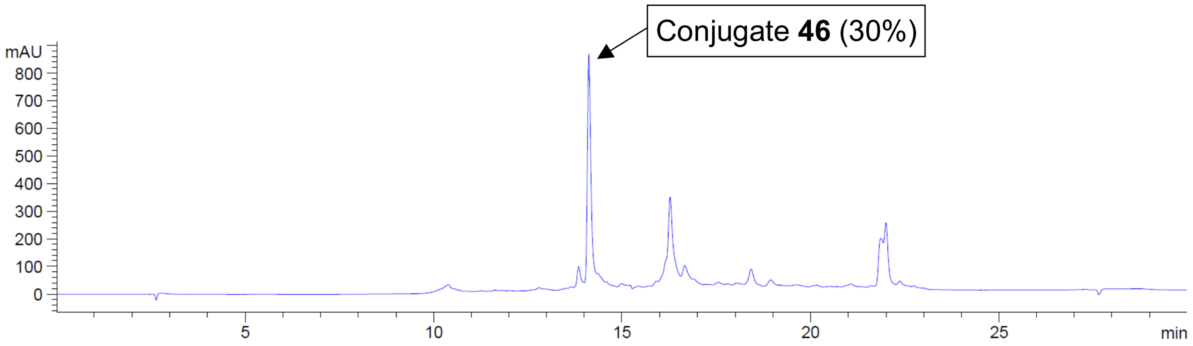

Figure S106. Analytical HPLC of crude conjugate ***46****.*

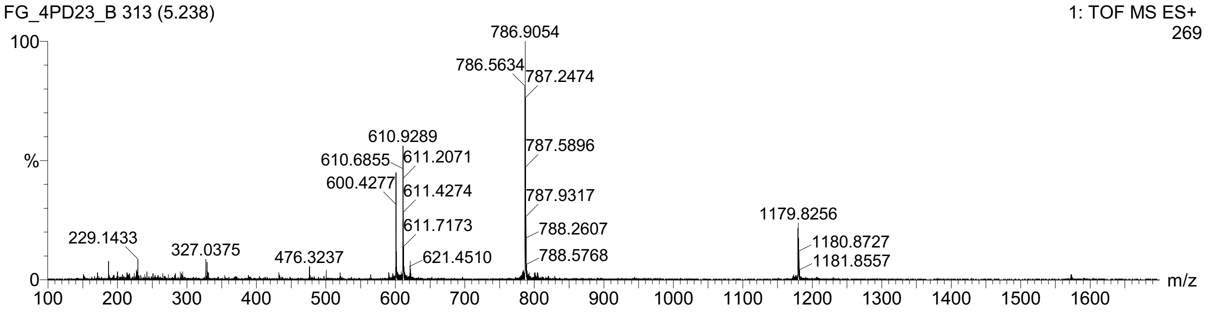

Figure S107. TIC from TOF MS ES+ analysis of crude conjugate ***46****.*

Conjugate 47

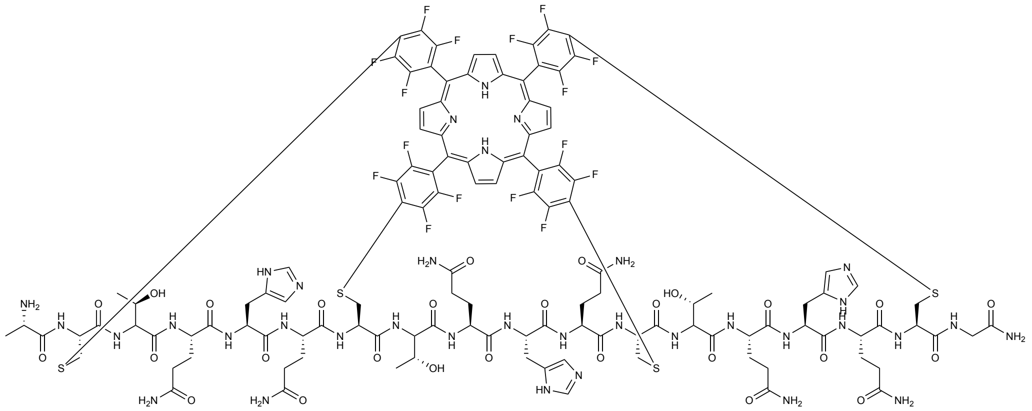

Calculated Exact Mass: 2933.8268 – Molecular Weight: 2935.7795 – RT: 11.2 min – ESI-MS (*m/z*): 1468.8330 $[M+2H]^{2+}$, 979.2777 $[M+3H]^{3+}$, 745.2099 $[M+3H+Na]^{4+}$, 734.9792 $[M+4H]^{4+}$– Isolated yield: 28%

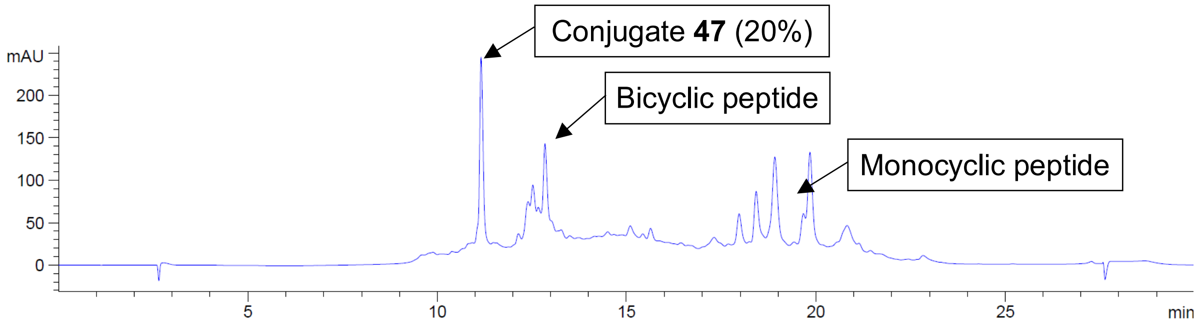

*Figure S108. Analytical HPLC of crude conjugate **47**.*

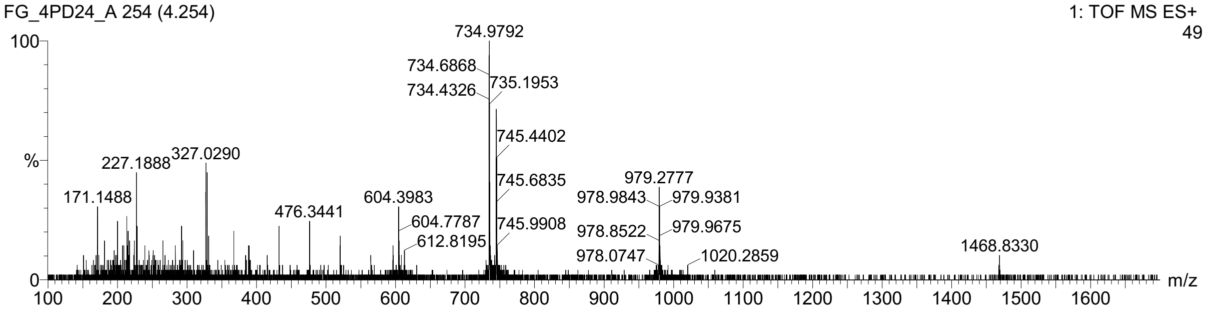

*Figure S109. TIC from TOF MS ES+ analysis of crude conjugate **47**.*

Conjugate 48

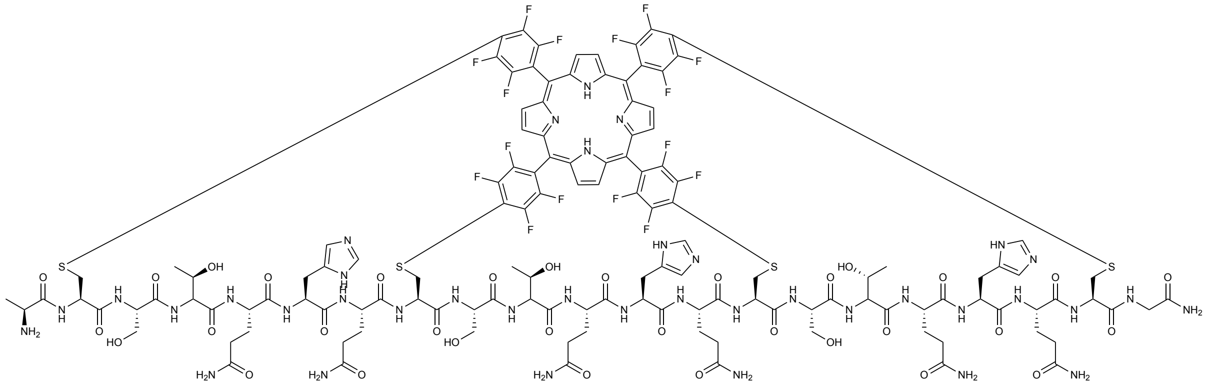

Calculated Exact Mass: 3194.9229 – Molecular Weight: 3197.0135 – RT: 11.0 min – ESI-MS (*m/z*): 1067.2273 $[M+3H]^{3+}$, 800.6884 $[M+4H]^{4+}$, 640.5292 $[M+5H]^{5+}$

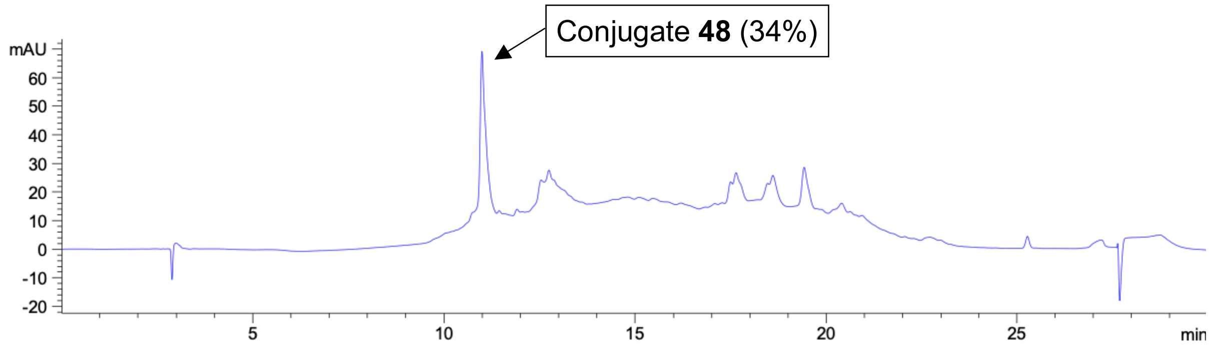

*Figure S110. Analytical HPLC of crude conjugate **48**.*

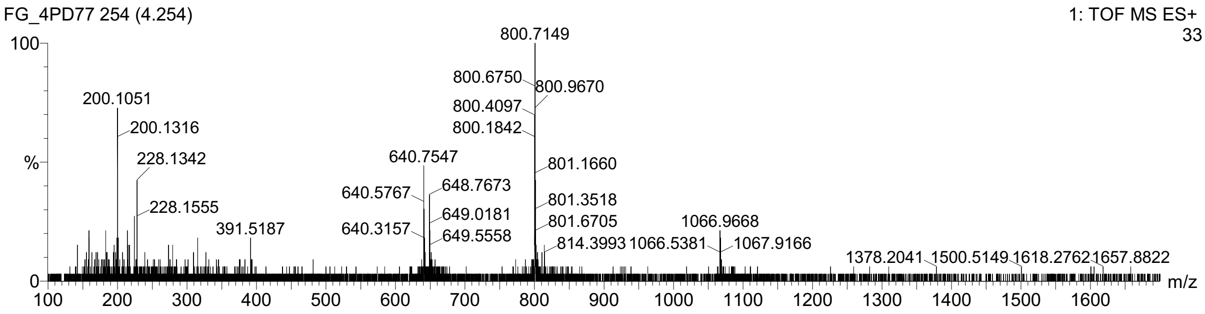

*Figure S111. TIC from TOF MS ES+ analysis of crude conjugate **48**.*

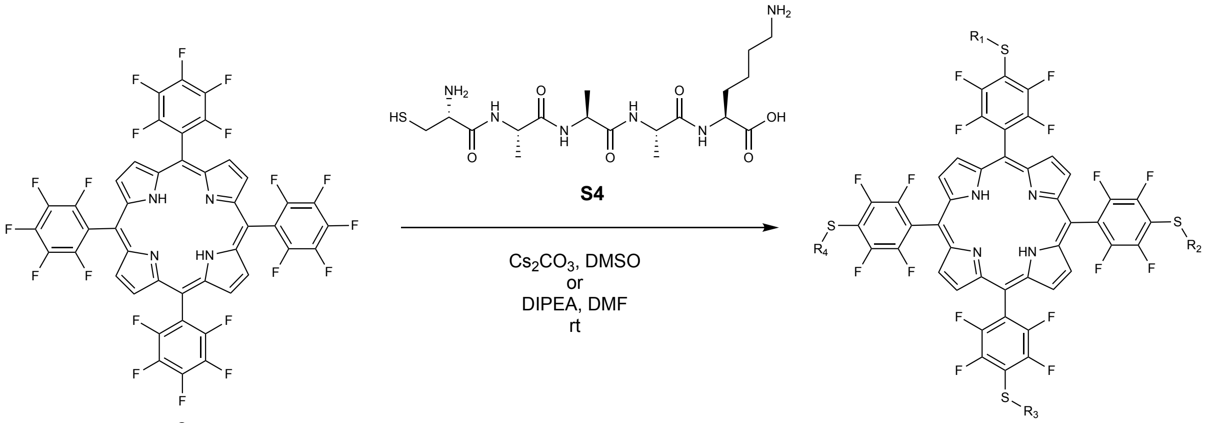

2

S5 R_1= CAAAK, R_2= R_3= R_4= H
S6 R_1= R_2= CAAAK, R_3= R_4= H
S7 R_1= R_3= CAAAK, R_2= R_4= H
S8 R_1= R_2= R_3= CAAAK, R_4= H
S9 R_1= R_2= R_3= R_4= CAAAK

Conjugate S5

Calculated Exact Mass: 1416.2784 – Molecular Weight: 1417.1197 – RT: see Figure S4/5/6 – ESI-MS (*m/z*): 1418.2040 $[M+H]^{+}$, 709.1124 $[M+2H]^{2+}$

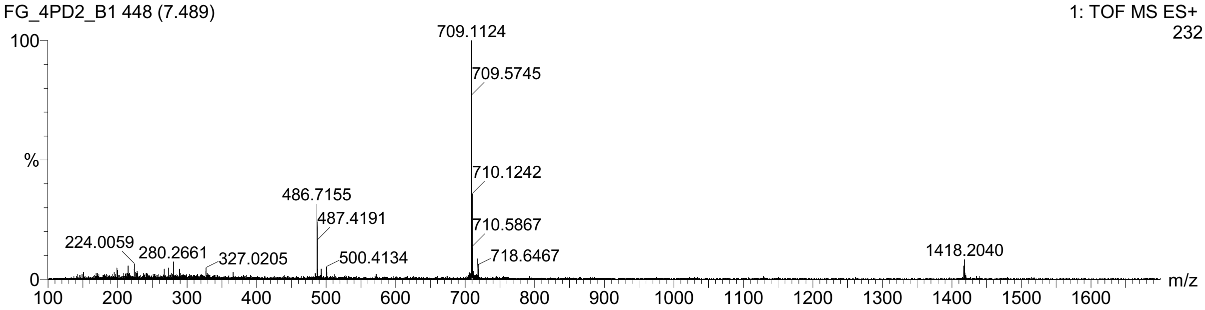

*Figure S112. TIC from TOF MS ES+ analysis of crude conjugate **S5**.*

Conjugate S6/7

Calculated Exact Mass: 1858.4983 – Molecular Weight: 1859.6793 – RT: see Figure S4/5/6 – ESI-MS (*m/z*): 930.1937 $[M+2H]^{2+}$, 620.4578 $[M+3H]^{3+}$

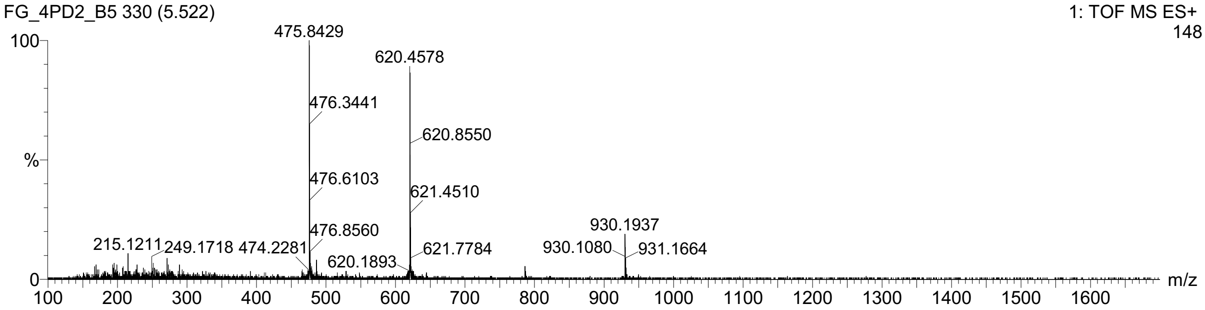

*Figure S113. TIC from TOF MS ES+ analysis of crude conjugate **S6/7**.*

Conjugate S8

Calculated Exact Mass: 2300.7181 – Molecular Weight: 2302.2389 – RT: see Figure S4/5/6 – ESI-MS (*m/z*): 768.5458 $[M+3H]^{3+}$, 576.1252 $[M+4H]^{4+}$

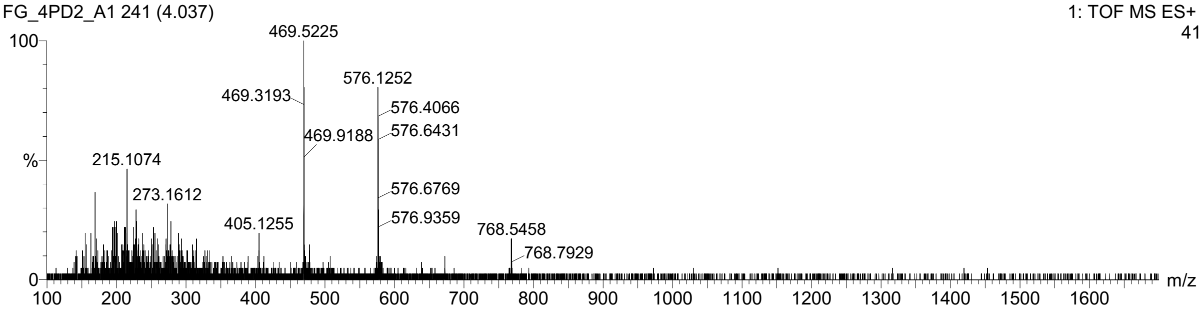

*Figure S114. TIC from TOF MS ES+ analysis of crude conjugate **S8**.*

Conjugate S9

Calculated Exact Mass: 2742.9379 – Molecular Weight: 2744.7985 – RT: see Figure S4/5/6 – ESI-MS (*m/z*): 916.0183 $[M+3H]^{3+}$, 687.1957 $[M+4H]^{4+}$, 549.7603 $[M+5H]^{5+}$

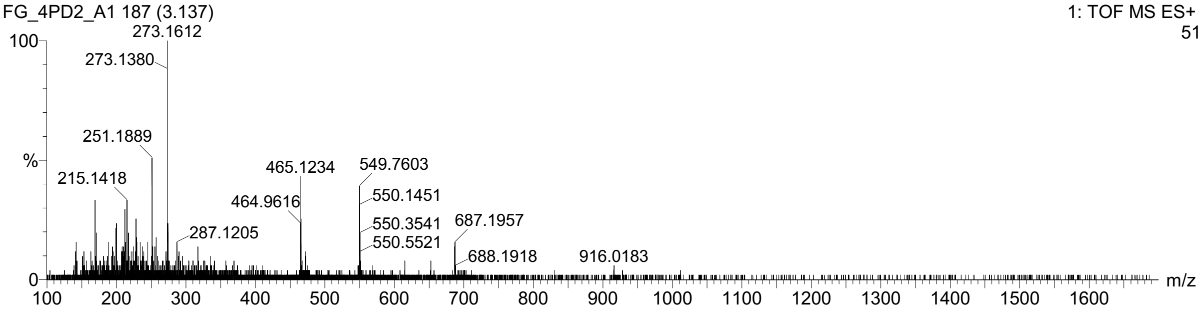

*Figure S115. TIC from TOF MS ES+ analysis of crude conjugate **S9**.*

Conjugates S10

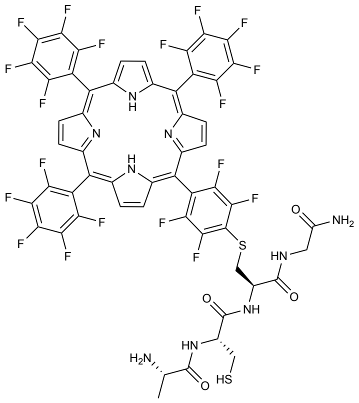

Calculated Exact Mass: 1305.1559 – Molecular Weight: 1305.9937 – RT: see below Figure S118 – ESI-MS (*m/z*): 1306.5295 $[M+H]^{+}$, 653.7870 $[M+2H]^{2+}$

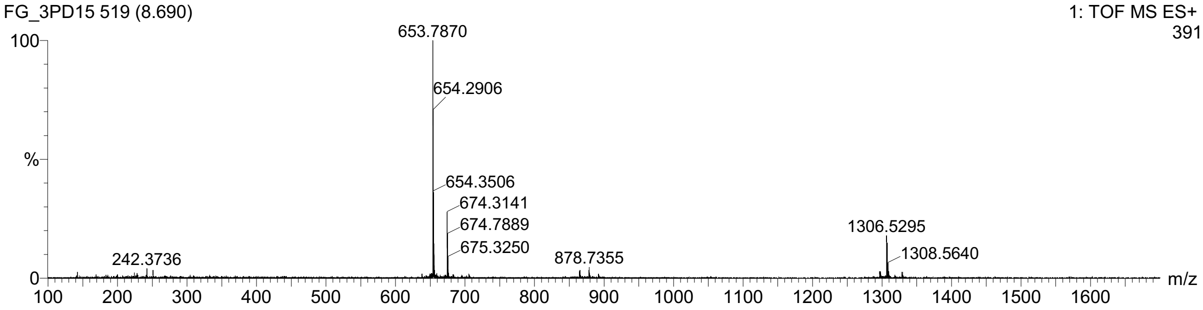

*Figure S116. TIC from TOF MS ES+ analysis of crude conjugate **S10**.*

Conjugates S11

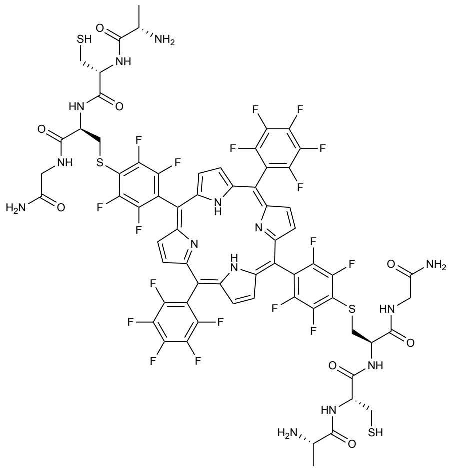

Calculated Exact Mass: 1636.2531 – Molecular Weight: 1637.4273 – RT: see below Figure S118 – ESI-MS (*m/z*): 819.3556 $[M+2H]^{2+}$, 546.5883 $[M+3H]^{3+}$

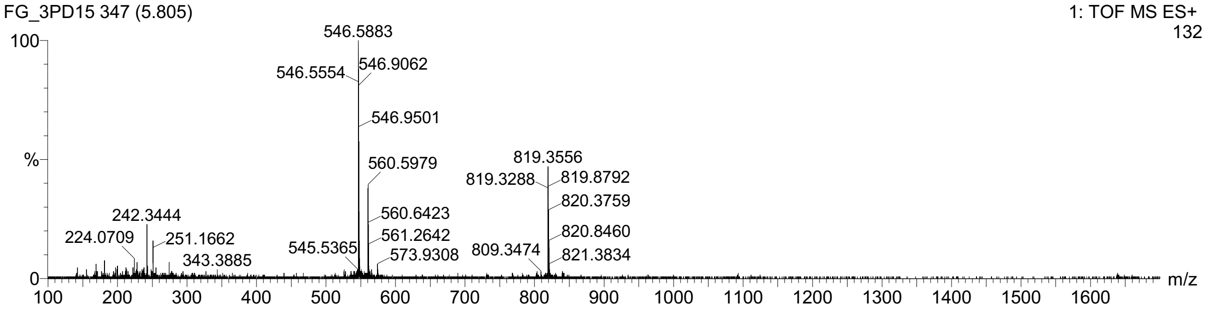

*Figure S117. TIC from TOF MS ES+ analysis of crude conjugate **S11.***

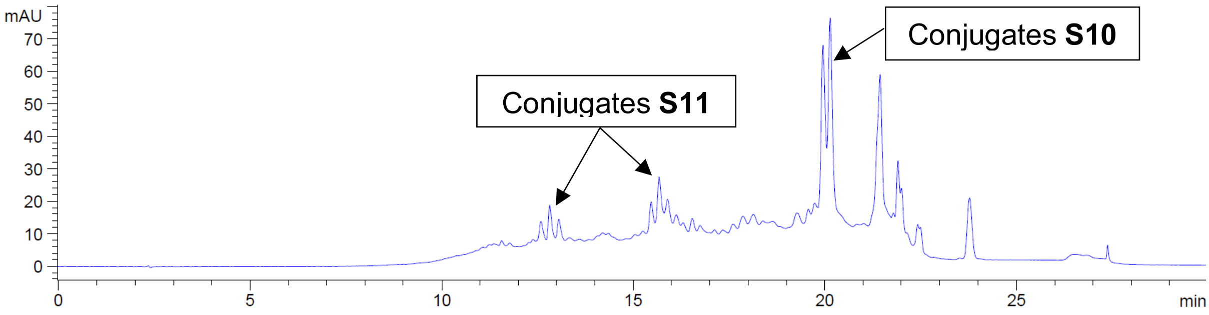

*Figure S118. Analytical HPLC of crude conjugates **S10** and **S11**.*
